# Genome-Wide Identification and Expression Profile of *TPS* Gene Family in *Dendrobium officinale* and the Role of *DoTPS10* in Linalool Biosynthesis

**DOI:** 10.3390/ijms21155419

**Published:** 2020-07-30

**Authors:** Zhenming Yu, Conghui Zhao, Guihua Zhang, Jaime A. Teixeira da Silva, Jun Duan

**Affiliations:** 1 Guangdong Provincial Key Laboratory of Applied Botany & Key Laboratory of South China Agricultural Plant Molecular Analysis and Genetic Improvement, South China Botanical Garden, Chinese Academy of Sciences, Guangzhou 510650, China; zhenming311@scbg.ac.cn (Z.Y.); zhaoconghui@scbg.ac.cn (C.Z.); zhanggh@scbg.ac.cn (G.Z.); 2 Center of Economic Botany, Core Botanical Gardens, Chinese Academy of Sciences, Guangzhou 510650, China; 3 University of Chinese Academy of Sciences, No. 19A Yuquan Road, Beijing 100049, China; 4 Independent Researcher, P.O. Box 7, Miki-cho Post Office, Ikenobe 3011-2, Kagawa-ken 761-0799, Japan; jaimetex@yahoo.com

**Keywords:** terpene synthase, terpenes, methyl jasmonate, abiotic stress, orchids

## Abstract

Terpene synthase (TPS) is a critical enzyme responsible for the biosynthesis of terpenes, which possess diverse roles in plant growth and development. Although many terpenes have been reported in orchids, limited information is available regarding the genome-wide identification and characterization of the TPS family in the orchid, *Dendrobium officinale*. By integrating the *D. officinale* genome and transcriptional data, 34 *TPS* genes were found in *D. officinale*. These were divided into four subfamilies (TPS-a, TPS-b, TPS-c, and TPS-e/f). Distinct tempospatial expression profiles of *DoTPS* genes were observed in 10 organs of *D. officinale*. Most *DoTPS* genes were predominantly expressed in flowers, followed by roots and stems. Expression of the majority of *DoTPS* genes was enhanced following exposure to cold and osmotic stresses. Recombinant DoTPS10 protein, located in chloroplasts, uniquely converted geranyl diphosphate to linalool in vitro. The *DoTPS10* gene, which resulted in linalool formation, was highly expressed during all flower developmental stages. Methyl jasmonate significantly up-regulated *DoTPS10* expression and linalool accumulation. These results simultaneously provide valuable insight into understanding the roles of the TPS family and lay a basis for further studies on the regulation of terpenoid biosynthesis by *DoTPS* in *D. officinale*.

## 1. Introduction

Terpenes, which are derived biosynthetically from two isomeric 5-carbon building blocks, dimethylallyl diphosphate (DMAPP) and isopentenyl diphosphate (IPP), are the largest family of plant secondary metabolites [1]. Plant terpenes play vital roles in attracting insect pollinators [2], plant defense response [1,3], plant–plant interactions [4], and the mediation of interactions with various ecological habitats [5]. The high volatility of terpene compounds promotes the scent in orchids. For instance, geraniol and linalool are major floral scent compounds in *Phalaenopsis bellina* [6,7]. Orchid floral volatiles, as well as flower color, shape, and fragrance are key horticultural ornamental traits in orchids, and also serve to attract pollinators in various ecological habitats [6].

**Figure 1:**
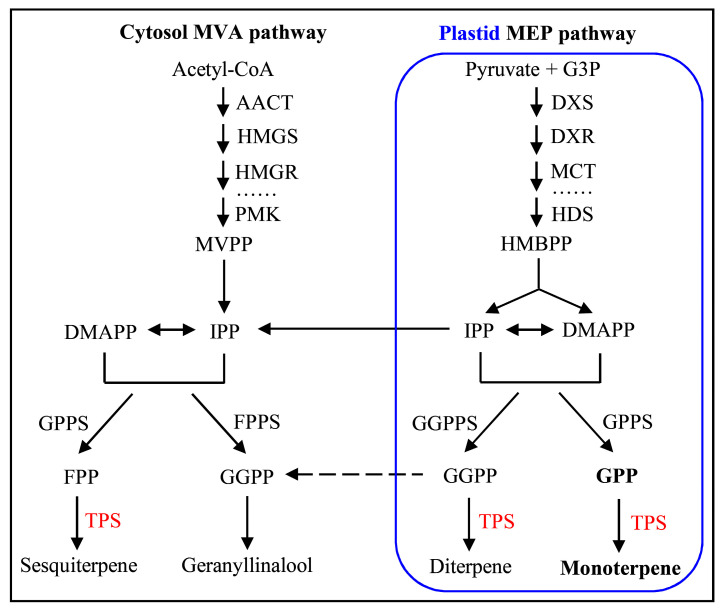
The pathway of terpene synthase genes responsible for the formation of terpenes in planta [3]. Terpenes are biosynthesized by the cytosol mevalonic acid (MVA) and the plastid methylerythritol phosphate (MEP) pathways, the former giving rise to sesquiterpenes and geranyllinalool, and the latter to monoterpenes and diterpenes. AACT, acetyl-CoA acetyltransferase; DMAPP, dimethylallyl pyrophosphate; DXS, 1-deoxy-d-xylulose 5-phosphate synthase; DXR, 1-deoxy-d-xylulose 5-phosphate reductoisomerase; FPP, farnesyl pyrophosphate; FPPS, FPP synthase; G3P, glyceraldehyde 3-phosphate; GGPP, geranylgeranyl pyrophosphate; GGPPS, GGPP synthase; GPP, geranyl pyrophosphate; GPPS, GPP synthase; HDS, 4-hydroxy-3-methylbut-2-en-1-yl diphosphate synthase; HMBPP, (E)-4-hydroxy-3-methylbut-2-en-1-yl diphosphate; HMGR, hydroxymethylglutaryl-CoA reductase; HMGS, hydroxymethylglutaryl-CoA synthase; IPP, isopentenyl pyrophosphate; MCT, 2-*C*-methyl-d-erythritol 4-phosphate cytidylyltransferase; MVPP, mevalonate 5-pyrophosphate; PMK, phosphomevalonate kinase; TPS, terpene synthase.

The biosynthetic pathway of volatile terpenes is well characterized in plants (Figure 1). Generally, the C5 precursors DMAPP and IPP are formed, and the direct precursors farnesyl diphosphate (FPP), geranyl diphosphate (GPP), and geranylgeranyl diphosphate (GGPP) are generated. Subsequently, plant terpenes are biosynthesized by terpene synthase (TPS), which converts FPP to sesquiterpene in the cytosol via the mevalonic acid (MVA) pathway, and GPP and GGPP to monoterpenes and diterpenes, respectively in plastids by the methylerythritol phosphate (MEP) pathway [1,8]. TPS is positioned at the branch point of the isoprenoid pathway, and is a key enzyme for terpenoid synthesis.

Each TPS is characterized by two conserved domains, PF03936 (*C*-terminal) and PF01397 (*N*-terminal) [9], as indicated in the Pfam (http://pfam.xfam.org/) database. The TPS family is phylogenetically classified into seven subfamilies (TPS-a, TPS-b, TPS-c, TPS-d, TPS-e/f, TPS-g, and TPS-h) [1]. Among them, TPS-a encodes sesquiterpene synthase that is found in both dicotyledonous and monocotyledonous plants. The angiosperm-specific TPS-b encodes monoterpene synthase with a R(R)X_8_W motif that catalyzes the isomerization cyclization reaction. TPS-c is deemed to belong to the ancestral clade and catalyzes copalyl diphospate synthase. The gymnosperm-specific TPS-d performs several functions, as diterpene, monoterpene, and sesquiterpene synthases. TPS-e/f encodes copalyl diphosphate/kaurene synthases, which are critical enzymes for the production of gibberellic acid. Another angiosperm-specific TPS-g encodes monoterpene synthase without the R(R)X_8_W motif. TPS-h is only observed in *Selaginella moellendorffii* [1,9,10,11]. In addition, TPS harbors conserved structural features such as DDxxD, NSE/DTE, and R(R)X_8_W motifs [1].

To date, TPS gene families have been identified at the genome-wide level in various plant species, including *Arabidopsis thaliana* [12], *Camellia sinensis* [13], *Daucus carota* [14], *Eucalyptus globulus* and *E. grandis* [15], *Malus domestica* [16], *Solanum lycopersicum* [17], *Selaginella moellendorffii* [18], and *Vitis vinifera* [19]. Orchids form one of the largest families of flowering plants, and their metabolic profile contains various terpenes [7]. Only a few *TPS* genes have been identified thus far in orchids. *PbTPS5* and *PbTPS10* might be involved in monoterpene biosynthesis in *Phalaenopsis bellina* [20]. *FhTPS1* catalyzes the formation of linalool, while *FhTPS4*, *FhTPS6*, and *FhTPS7* are bifunctional enzymes that can simultaneously recognize FPP and GPP as substrates [21]. However, no comprehensive study about *TPS* genes in *Dendrobium officinale* exists.

*D. officinale* is an endangered orchid native to South and Southeast Asia, and is used for medicinal purposes in Chinese culture [22]. Moreover, *D. officinale* is a unique orchid because it grows on rocks, trees, or even cliffs. In order to adapt to harsh growth conditions, terpene compounds are synthesized [6,22,23,24,25]. Therefore, it is necessary to characterize the *TPS* gene family and study the roles of TPS in *D. officinale*. These findings will provide a valuable reference about the terpene biosynthetic pathway in orchids.

## 2. Results

### 2.1. Genome-Wide Identification and Features of TPS Proteins in D. officinale

To systematically identify the *TPS* genes in *D. officinale*, a hidden Markov model (HMM) profile of the conserved *C*-terminal (PF03936) and *N*-terminal (PF01397) domains in the TPS protein was used as a BLAST query against the *D. officinale* genome database [23]. After the removal of redundant sequences, 34 TPS genes were obtained (Table 1). The open reading frame (ORF) of *DoTPS* ranged from 378 (*DoTPS12*) to 2571 bp (*DoTPS4*), the deduced length of the amino acids ranged from 125 (DoTPS12) to 856 aa (DoTPS4), and molecular weight (Mw) ranged from 14.98 (DoTPS12) to 100.05 kDa (DoTPS4). The theoretical isoelectric point (pI) values of DoTPS proteins ranged from 4.94 (DoTPS19) to 7.18 (DoTPS4). The calculated grand average of hydrophobicity (GRAVY) values, ranging from −0.429 (DoTPS4) to 0.013 (DoTPS19), indicated most DoTPS proteins were hydrophilic, except for DoTPS19 with a GRAVY value > 0. In addition, the aliphatic index (AI) of DoTPS proteins ranged from 80.35 (DoTPS4) to 110.46 (DoTPS4), and the instability index (II) of these proteins ranged from 33.82 (DoTPS20) to 51.56 (DoTPS31). According to three widely used predictors (AtSubP [26], Plant-mPLoc [27], and pLoc-mPlant [28], all having good accuracy with greater than 70%), 14/34 DoTPS proteins were targeted to the chloroplast, other 20 DoTPS proteins were targeted to chloroplast or cytoplasm (Table 1, Tables S1–S3), suggesting that different predictors produce different results, and it was better to verify by experimental results. The prediction of secondary structures demonstrated that α-helixes and random coils were dominant in all DoTPS proteins, followed by extended strands and β-turns, accounting for on average 68.68, 23.82, 4.33, and 3.17%, respectively (Table S4).

### 2.2. Analysis of Conserved Motifs and Gene Structure

**Figure 2:**
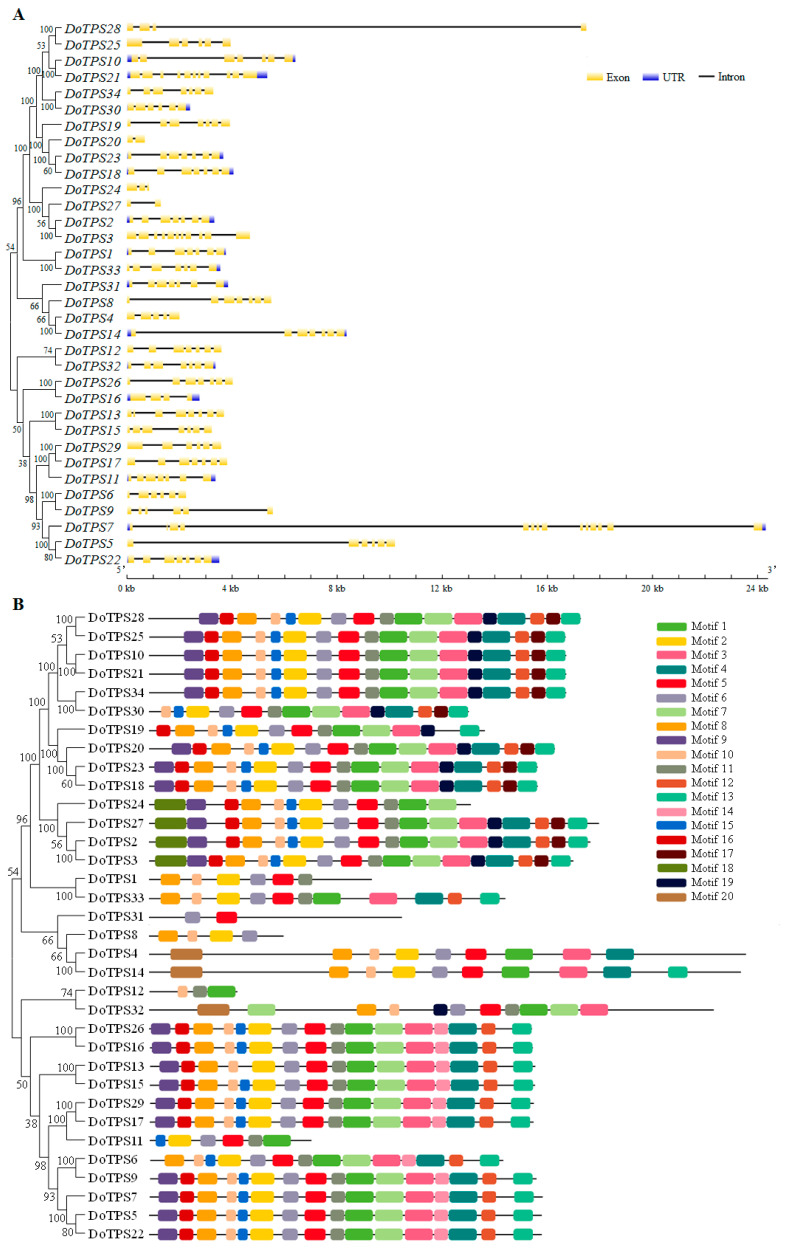
Phylogenetic relationships, exon–intron structure, and protein domain analysis of *DoTPS* genes in *D. officinale*. (**A**) Phylogenetic relationships and exon–intron structure of *DoTPS* genes. Exon–intron distribution was performed using GSDS 2.0 server (http://gsds.cbi.pku.edu.cn/). Yellow boxes indicate exons, black lines indicate introns. Blue boxes represent upstream/downstream-untranslated regions. (**B**) Phylogenetic relationships and motif structures of *DoTPS* genes. Phylogenetic tree was generated with MEGA 7.0 using the NJ method. Twenty classical motifs in DoTPS proteins were analyzed by the MEME tool. The width of each motif ranged from 15 to 47 amino acids. Different color blocks represented different motifs.

Since analysis of gene structure will facilitate an understanding of gene evolution and possible roles, the structure of *DoTPS* genes in *D. officinale* was investigated (Figure 2A). The amount of exons ranged from 2 to 14, with an average of 6.6 for all *DoTPS* genes. *DoTPS32* contained the most exons (14), whereas *DoTPS8* and *DoTPS12* harbored the fewest exons (2). The majority of *DoTPS* genes (52.9%) had seven exons. Apart from *DoTPS7*, *-20*, *-24*, *-27*, and *-28*, most of the genes that clustered in the same group generally possessed a similar exon–intron structure, especially in terms of intron number and exon length (Figure 2A). This conserved exon–intron structure within each cluster was in agreement with the classification of *DoTPS* genes in a neighbor-joining (NJ) phylogenetic tree based on DoTPS sequences.

To further elucidate the structural and functional features of DoTPS, 20 conserved motifs of the DoTPS proteins were identified using MEME software (Figure 2B). The lengths of these motifs ranged from 15 to 47 amino acids (Figure S1; Table S5). DoTPS3 contained the most motifs (18/20) while DoTPS31 had only two motifs. Motif 6 was found in all DoTPS proteins, except DoTPS12. Motifs 5 and 10 were the second most common DoTPS proteins (32/34), followed by motifs 1 and 2 (31/34). DoTPS1, -8, and -31 did not contain the DDxxD motif (motif 1), and DoTPS1, -12, and -31 did not contain the R(R)X_8_W (motif 2) motif. Intriguingly, motif 14 was found in the cluster containing DoTPS5, -6, -7, -9, 13, -15, -16, -17, -22, -26, and -29. Motifs 17 and 20 were particularly abundant in the group containing DoTPS2, -3, -10, -18, -19, -20, -21, -23, -25, -27, -28, -30, -32, and -34. Motif 20 only existed in the cluster that included DoTPS4, -14, and -32. Motif 18 was only observed in a small branch that harbored DoTPS2, -3, -24, and -27 (Figure 2B). Despite the different types of motifs among clusters, DoTPS proteins within the same cluster generally possessed similar motifs. The diversity of DoTPS phylogenetic grouping patterns was likely influenced by the gene structure and the location of motifs.

### 2.3. Phylogenetic Analysis of DoTPS Genes in D. officinale

**Figure 3:**
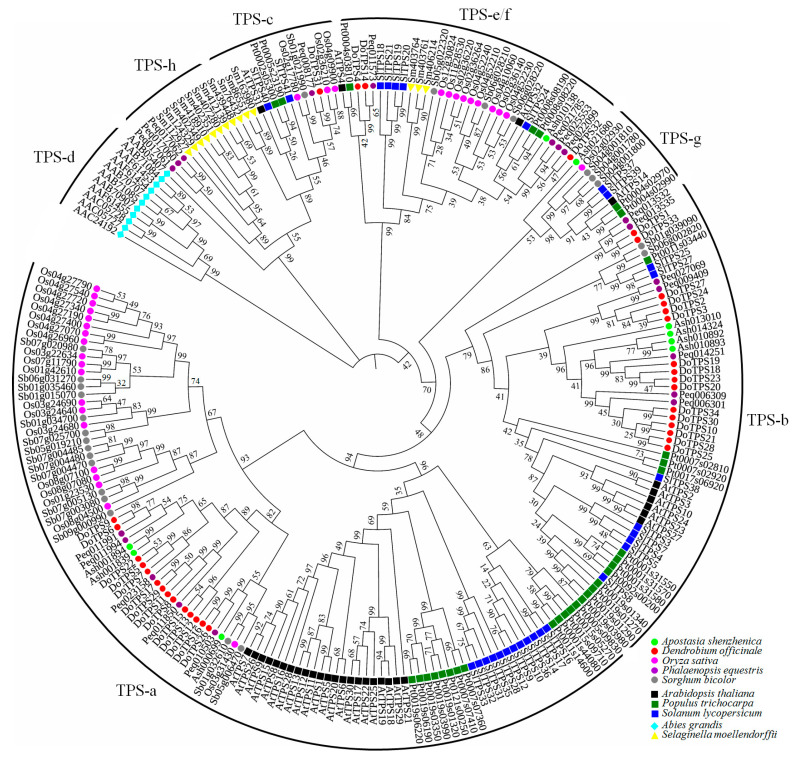
Phylogenetic analysis of TPS proteins in ten higher plant species. A phylogenetic tree was constructed by the neighbor-joining method with the Jones–Taylor–Thornton model and pairwise deletion option using MEGA 7.0 with 1000 bootstrap replicates. Tree visualization and labeling was performed on FigTree v1.4.4 (http://tree.bio.ed.ac.uk/software/figtree/). The TPS family was divided into seven subfamilies as previously reported [1,15]: TPS-a, TPS-b, TPS-c, TPS-d, TPS-e/f, TPS-g, and TPS-h. Circles represented monocotyledonous plants, squares represented dicotyledonous plants, the cyan diamond indicates *Abies grandis*, and yellow triangle indicates *Selaginella moellendorffii*.

To gain further insight into the evolutionary relationships among the TPS subfamilies, an unrooted phylogenetic tree was constructed using the neighbor-joining (NJ) method implemented in MEGA 7.0 with the Jones–Taylor–Thornton (JTT) model based on multiple sequence alignment of TPS members from *Abies grandis*, *A. thaliana*, *Apostasia shenzhenica*, *D. officinale*, *Oryza sativa*, *Phalaenopsis equestris*, *Populus trichocarpa*, *Selaginella moellendorffii*, *Solanum lycopersicum*, and *Sorghum bicolor* (Figure 3, Table S6). The phylogenetic tree demonstrates that TPS proteins were clustered into seven subfamilies (TPS-a, TPS-b, TPS-c, TPS-d, TPS-e/f, TPS-g, and TPS-h) according to the published report [1,15]. Thirty-four DoTPS proteins appeared in only four groups (TPS-a, TPS-b, TPS-c, and TPS-e/f, 14, 16, 1, and 3, respectively), and 88.2% of them belonged to TPS-a or TPS-b subgroups. Similarly, there were 20 and 9 TPS proteins present in *P. equestris* and *A. shenzhenica*, the amount of TPS-a, and TPS-b accounted for 12/20, and 7/9, respectively (Figure 3). This phenomenon was similar to that in *A. thaliana* and *O. sativa* [1,10]. Remarkably, in the TPS-a group, dicotyledonous and monocotyledonous plants formed distinct subgroups, which were observed previously [1,15] and termed them as TPS-a1 (dicots) and TPS-a2 (monocots), suggesting that the *TPS-a* genes evolved independently. Similar to the TPS-a group, the TPS-c group was further divided into dicot and monocot subclades. Consistent with a previous report, the TPS-d subfamily was specific to gymnosperms, and the TPS-h subfamily was only observed in *S. moellendorffii* [1,11,12,17], inferring that they might play a particular role in these species.

**Figure 4:**
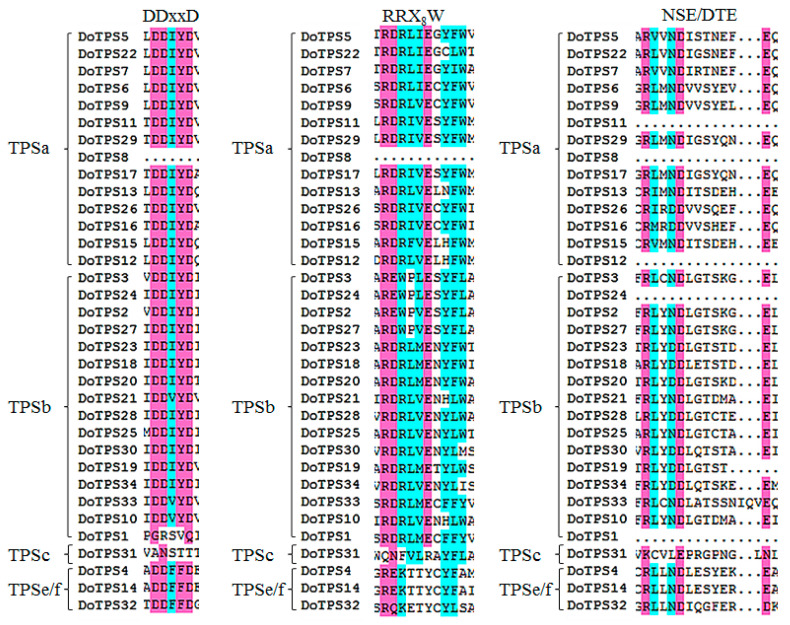
Comparison of DDxxD, R(R)X_8_W, and NSE/DTE motifs in *D. officinale* DoTPS proteins.

Multiple sequence alignment of DoTPS proteins was further analyzed. As illustrated in Figure 4, the arginine-tryptophan motif, R(R)X_8_W, was found in all the DoTPS-a and DoTPS-b proteins, except DoTPS8, at the *N*-terminus. It plays a role in initiation of the isomerization cyclization reaction [1,10]. However, the arginine-tryptophan motif, R(R)X_8_W, varied or was even absent in TPS-c and TPS-e/f proteins. Two aspartate-rich motifs, DDxxD and NSE/DTE, are essential for cleaving prenyl diphosphate substrate by chelating a trio of Mg^2+^ or Mn^2+^ at the *C*-terminus [9,11]. The DDxxD motif was conserved in almost all the DoTPS proteins, except three DoTPS proteins (DoTPS1, DoTPS8 of the TPS-a group, and DoTPS31 of the TPS-c group). DoTPS31 was the only TPS-c member in *D. officinale*. The TPS-c subfamily is mainly found in land plants and its prenyl diphosphate unit is not cleaved [1]. The NSE/DTE motif was absent in DoTPS8, -11, and -12 of the TPS-a group, DoTPS1, -19, and -24 of the TPS-b group, and DoTPS32 of the TPS-e/f group (Figure 4). Taken together, gene structure and amino acid alignment were consistent with the phylogenetic analysis. The functions of DoTPS proteins in the same group could be inferred from known TPS proteins, according to their phylogenetic relationships.

### 2.4. Identification of Cis-Acting Elements in the Promoter Region of DoTPS Genes

**Figure 5:**
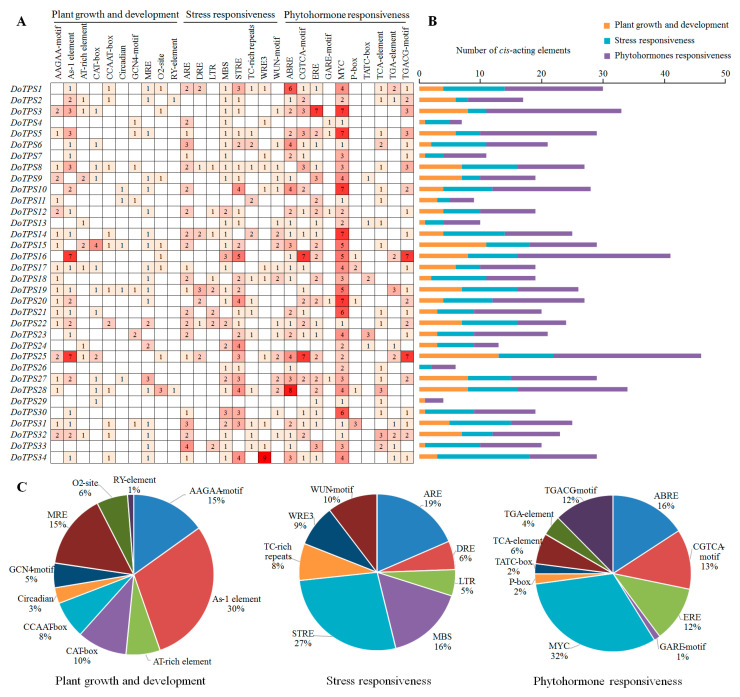
Information of *cis*-acting elements in *DoTPS* genes of *D. officinale*. (**A**) The gradient red colors and numbers in the grid indicate the number of different *cis*-acting elements in *DoTPS* genes. (**B**) The different colored histogram indicates the number of *cis*-acting elements in each category. (**C**) The ratio of different *cis*-acting elements in each category is shown as pie charts.

To ascertain the potential biological roles of *DoTPS* genes in *D. officinale*, a 2000-bp upstream region of the initiation code (ATG) was identified using the PlantCARE tool. The *cis*-acting elements in the promoter regions of *DoTPS* genes were classified into three categories of *cis*-elements linked to plant growth and development, phytohormone responsiveness, and stress responsiveness (Figure 5). In the plant growth and development category (159/759), 10 *cis*-elements involved in circadian rhythms, endosperm expression (AAGAA-motif and GCN4-motif), flowering (AT-rich element, CCAAT-box, and MRE), shoot and root meristem expression (CAT-box), seed expression (RY-element), shoot expression (As-2 element), and zein metabolism (O_2_ site) were found, the highest proportion being the As-2 element (30%). In the stress responsiveness category (221/759), various elements related to anaerobic induction (ARE, 19%), defense and stress (TC-rich repeats, 8%), dehydration (DRE, 6%), drought-inducibility (MBS, 16%), low temperature (LTR, 5%), stress (STRE, 27%), and wounding (WRE3 and WUN-motif, 9% and 10%, respectively) responsiveness were detected. Most of *cis*-elements (379/759) were related to the phytohormone responsiveness category, and were responsive to abscisic acid (ABRE), auxin (TGA-element), ethylene (ERE), gibberellin (GARE-motif, P-box, and TATC-box), MeJA (TGACG-motif and MYC), and salicylic acid (TCA-element). Notably, the largest number of *cis*-elements was the TGACG-motif and MYC associated with MeJA-responsiveness, accounting for 12% and 29% of the hormone-related *cis*-elements, respectively (Figure 5). These results suggest that *DoTPS* genes might be MeJA-induced and/or -repressed genes, and that they respond to multiple abiotic stresses.

### 2.5. Tempospatial Expression Patterns of DoTPS Genes in Different D. officinale Organs

**Figure 6:**
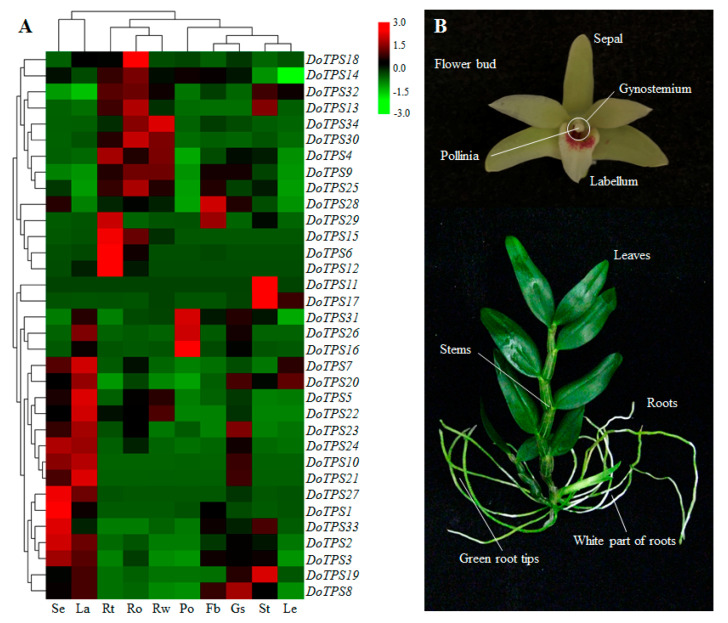
Tissue-specific expression profiles of *DoTPS* genes in different *D. officinale* organs. (**A**) The transcription levels of *DoTPS* genes in different tissues. The different tissues were sepals (Se), labellum (la), green root tips (Rt), roots (Ro), white part of roots (Rw), pollinia (Po), flower buds (Fb), gynostemium (Gs), stems (St), and leaves (Le) in two-year-old *D. officinale* adult plants. Heatmap was generated using the TBtools server (https://github.com/CJ-Chen/TBtools), and gradient color from green to red was expressed as the log2-transformed expression levels of each *DoTPS* gene that was normalized to the internal reference gene *DoEF-1α*, GenBank accession no. JF825419. (**B**) *D. officinale* “Zhongke 5” used in this study. All fragments per kilobase of transcript per million fragments mapped (FPKM) values that were used were downloaded from NCBI under BioProject PRJNA262478 [25], and are listed in Table S7.

To obtain clues about the role of *DoTPS* genes in *D. officinale* development, an RNA-sequencing transcriptome database of flower buds, green root tips, gynostemium (column), labellum (lip), leaves, pollinia, sepals, stems, roots, and white part of roots was established (Figure 6A). Overall, *DoTPS* genes exhibited distinct organ-specific expression profiles, possibly suggesting the functional divergence of *DoTPS* genes in different *D. officinale* tissues during growth and development. *DoTPS11*, *-17*, and *-19* were highly expressed in stems. Thirteen *DoTPS* genes exhibited a high level of expression in root tissues, including five (*DoTPS4*, *-6*, *-12*, *-15*, and *-29*), five (*DoTPS13*, *-14*, *-18*, *-25*, and *-32*), and three (*DoTPS9*, *-30*, and *-34*) genes in green root tips, roots, and white part roots, respectively. Notably, 52.9% of *DoTPS* genes displayed the highest transcript abundance in floral organs. Among them, *DoTPS28* in flower buds, *DoTPS8* in gynostemium, *DoTPS5*, -7, -10, *-20*, *-21*, *-22*, and *-23* in labellum, *DoTPS16*, *-26*, and *-31* in pollinium, and *DoTPS1*, *-2*, *-3*, *-24*, *-27*, and *-33* in sepals, indicating that the preferentially expressed *DoTPS* genes might be indirectly or directly involved in the development of reproductive organs. Our data indicates that the organ-specific expression of *DoTPS* genes might be important in *D. officinale* flower growth and development.

### 2.6. Expression Patterns of DoTPS Genes under Abiotic Stress

**Figure 7:**
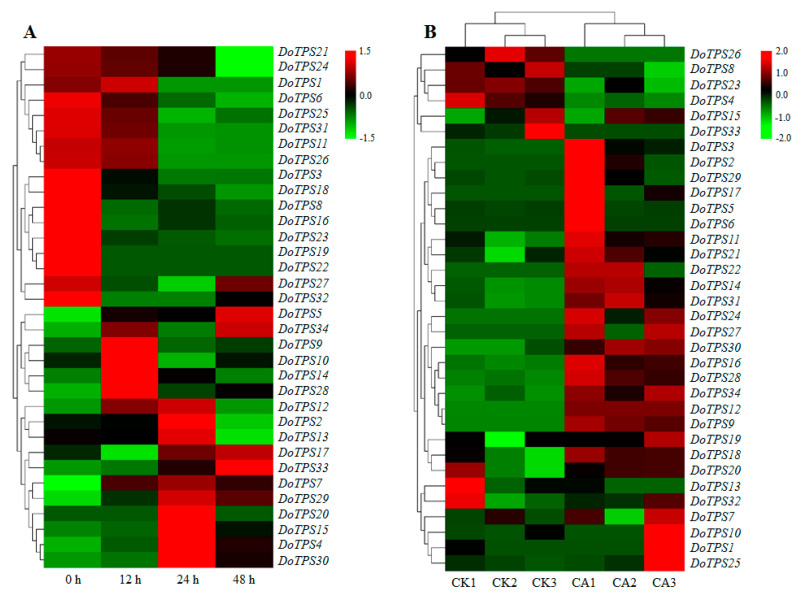
Transcription levels of *DoTPS* genes in *D. officinale* under cold and osmotic stresses. (**A**) Expression profiles of *DoTPS* genes in response to 200 mM mannitol treatment for 48 h. (**B**) Expression profiles of *DoTPS* genes in response to cold treatment (0 °C) for 20 h. Heatmap was drawn using TBtools software (https://github.com/CJ-Chen/TBtools). Color gradient from green to red was expressed as the log2-transformed expression level of each *DoTPS* gene. CA, cold acclimation; CK, control (non-acclimation). The expression values of *DoTPS* genes in response to mannitol treatment are listed in Table S8. The FPKM values of *DoTPS* genes exposed to cold treatment that were downloaded from a transcriptome database [29], are listed in Table S9.

To better understand the role of *DoTPS* genes in response to cold and osmotic stresses, transcriptome data combined with the RT-qPCR assay were employed to investigate the expression levels of *DoTPS* genes in *D. officinale* under cold (0 °C) or osmotic (mannitol) treatment. Results showed that *DoTPS* genes exhibited distinct expression patterns under osmotic treatment, showing two trends, an upward trend and a downward trend (Figure 7A). Half of the *DoTPS* genes, including *DoTPS1*, *-3*, *-6*, *-8*, *-11*, *-16*, *-18*, *-19*, *-21*, *-22*, *-23*, *-24*, *-25*, *-26*, *-27*, *-31*, and *-32*, were obviously suppressed (1.2–48.7-fold) by mannitol-induced osmotic stress. The other half of *DoTPS* genes exhibited an increasing trend, but the highest expression level was either at 12 h (*DoTPS9*, *-10*, *-14*, and *-28*), 24 h (*DoTPS2*, *-4*, *-7*, *-12*, *-13*, *-15*, *-20*, *-29*, and *-30*), or 48 h (*DoTPS5*, *-17*, *-33*, and *-34*) in response to mannitol treatment (Figure 7A).

After cold acclimation (0 °C) for 20 h, 26 *DoTPS* genes were upregulated, more than the number of suppressed genes (8; Figure 7B). Compared to the non-acclimated controls, the transcription levels of *DoTPS4*, *-8*, *-13*, *-15*, *-23*, *-26*, and *-33* were downregulated between 1.6- and 14.3-fold. In contrast, the expression levels of most other *DoTPS* genes (27/34) were clearly upregulated between 1.9- and 103.1-fold, except for the tiny variation of *DoTPS20* and *DoTPS32*. These results suggested that *DoTPS* genes might be involved in cold and osmotic stress responses in *D. officinale*.

### 2.7. Expression Patterns of DoTPS Genes Subjected to MeJA Treatment

**Figure 8:**
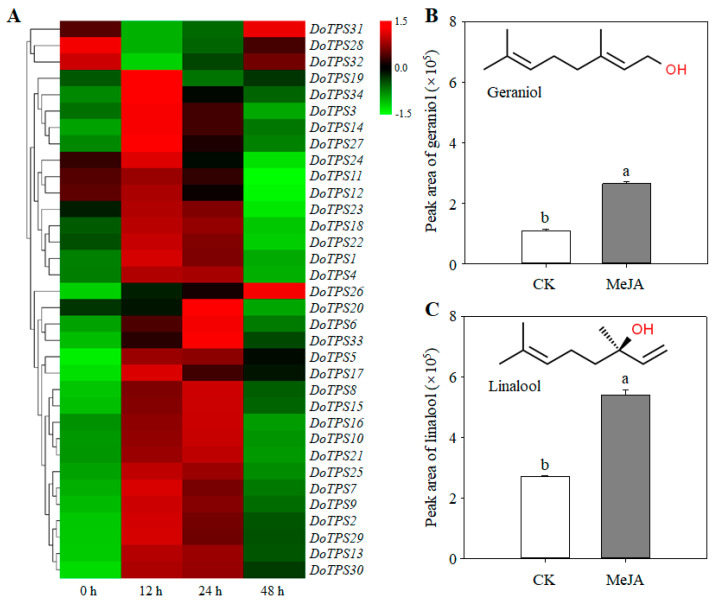
The transcription levels of *DoTPS* genes and synthesis of geraniol and linalool in *D. officinale* after MeJA treatment. (**A**) Effect of MeJA treatment on the expression of *DoTPS* genes. (**B**) Effect of MeJA treatment on the synthesis of geraniol. (**C**) Effect of MeJA treatment on the synthesis of linalool. Ten 10-month-old *D. officinale* seedlings exposed to 1 mM MeJA for 0, 12, 24, and 48 h were harvested. Heatmap was created using the TBtools software (https://github.com/CJ-Chen/TBtools), and gradient color from green to red was expressed as the log2-transformed expression levels of each *DoTPS* gene. Each bar represents the mean (±standard error, *n* = 3) of three independent biological replicates. Different letters above the bars indicated significant differences (*p* < 0.05, Duncan’s multiple range test). CK, control treatment without MeJA. MeJA, methyl jasmonate. The expression values of *DoTPS* genes in response to MeJA treatment are listed in Table S10.

MeJA is a signaling molecule that promotes the formation of secondary metabolic products [26]. We determined the effect of MeJA at the level of transcription of *DoTPS* genes in *D. officinale*. MeJA treatment differentially regulated *DoTPS* gene expression. Compared to the non-treated control, *DoTPS28*, *-31*, and *-32* were suppressed between 1.5- and 2.4-fold at 12 h after MeJA treatment. The suppressed genes returned to their control level at 48 h after MeJA treatment. In contrast, 31 *DoTPS* genes were upregulated between 1.2- and 45.1-fold, with the highest expression at 12 h (21/31), 24 h (8/31), or 48 h (2/31) after MeJA treatment (Figure 8A), but others showed reduced gene expression. Terpenes are dominant floral volatiles of orchids, especially geraniol and linalool [7,24]. Moreover, after treatment with MeJA, the amount of geraniol and linalool was significantly enhanced (Figure 8B). *DoTPS* genes were inducible by MeJA, which might be related to the *cis*-acting elements present in their promoters, resulting in the increased formation of terpenes in *D. officinale*.

### 2.8. Transcription Abundance of DoTPS Genes at Budding and Flowering Stages

**Figure 9:**
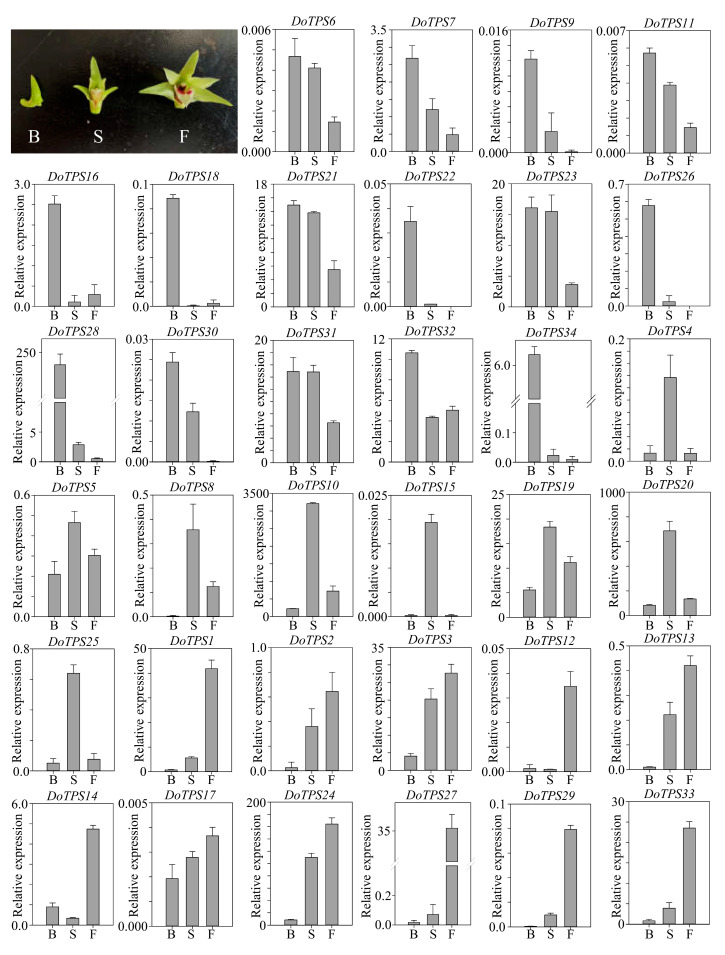
Transcription levels of *DoTPS* genes in *D. officinale* at three flowering stages: budding (B), semi-flowering (S) and full flowering (F). The levels of transcription were calculated by the 2^−ΔΔCT^ method and normalized to the C_T_ value of *DoEF-1α*. Each bar represents the mean (±standard error, *n* = 3) of three independent biological replicates. Different letters above bars indicate significant differences (*p* < 0.05, Duncan’s multiple range test). The expression values of *DoTPS* genes at three flowering stages of *D. officinale* are listed in Table S11.

Since the majority of *DoTPS* genes were highly expressed in floral organs (Figure 6), we further investigated their spatial expression patterns at three floral developmental stages. *DoTPS6*, *-7*, *-9*, *-11*, *-16*, *-18*, *-21*, *-22*, *-23*, *-26*, *-28*, *-30*, *-31*, *-32*, and *-34* were mainly expressed in the floral budding stage. *DoTPS4*, *-5*, *-8*, *-10*, *-15*, *-19*, *-20*, and *-25* were prominently expressed during the semi-flowering stage. The remaining genes (*DoTPS1*, *-2*, *-3*, *-12*, *-13*, *-14*, *-17*, *-24*, *-27*, *-29*, and *-33*) displayed greatest expression levels at the full flowering stage (Figure 9). *DoTPS* genes responsible for floral fragrance showed significant differences among the three floral developmental stages. Notably, DoTPS10 had the highest level of transcription during the floral developmental stages (Figure 9), suggesting that DoTPS10 may be responsible for the biosynthesis of geraniol or linalool. Further functional characterization of the *DoTPS10* gene could be helpful.

### 2.9. Subcellular Localization of DoTPS10 in Heterologous Plants

**Figure 10:**
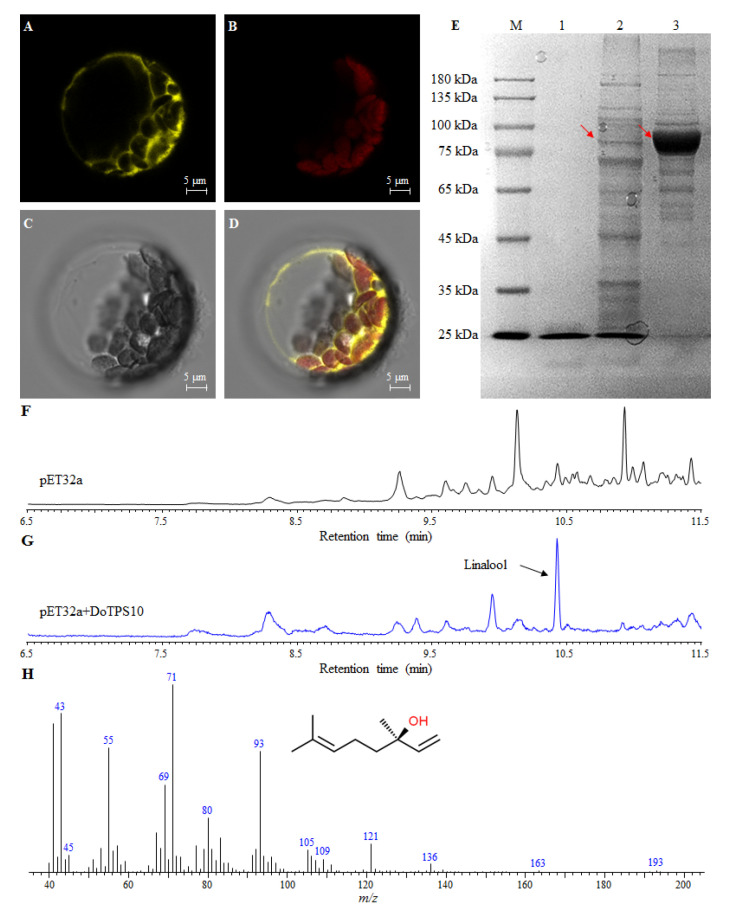
Functional characterization of *DoTPS10*. (**A**–**D**) Subcellular localization of DoTPS10 fused with yellow fluorescent protein (YFP) and transiently expressed in *A. thaliana* protoplasts. Bars = 5 μm. (**E**) SDS-PAGE analysis of DoTPS10 recombinant protein expressed in *Escherichia coli* BL21. Lanes M, 1, 2, and 3 indicate marker, pET32a, crude DoTPS10 protein, and purified DoTPS10 protein, respectively. Red arrow indicates target protein. (**F**–**G**) Gas chromatograms of products yielded by DoTPS10 using GPP as a substrate. (**H**) Mass spectrum of linalool was identical to the mass spectrum of the linalool standard.

DoTPS10 was assigned to the chloroplast with a 46 aa transit peptide. To validate the prediction, YFP-tagged DoTPS10 fusions were transiently expressed in *A. thaliana* protoplasts. In vivo YFP fluorescence signals from DoTPS10 were observed in chloroplasts (Figure 10A–D), which were consistent with a previous report of FhTPS1, FhTPS2, FhTPS4, and FhTPS5 in a *Freesia* hybrid [21].

### 2.10. Functional Characterization of DoTPS10 Involved in the Formation of Linalool

Generally, monoterpene volatiles are the main terpenes in orchids. These are produced by TPS proteins using GPP as a substrate [6,7]. To further confirm the role of DoTPS10 in the synthesis of monoterpenes, a His-tagged vector (pET32a) was used to produce the recombinant DoTPS10 protein. The vector was successfully expressed as a soluble protein in *E. coli* BL21. DoTPS10 protein was purified using a His-trap Ni-sepharose high performance column. His-tagged vector (pET32a) had an Mw of 23.8 kDa, containing 4.8 kDa of six His-tags, and recombinant DoTPS10 protein exhibited an approximate Mw of 88.3 kDa on SDS-PAGE (Figure 10E). After recombinant DoTPS10 protein was incubated with GPP, gas chromatography–mass spectrometry (GC-MS) analysis showed that empty pET32a could not produce linalool while the recombinant DoTPS10 protein singly converted the substrate GPP to the corresponding product linalool (Figure S3, Figure 9G,H).

## 3. Discussion

*D. officinale* is widely grown in subtropical and temperate regions and used as a health food, but also has a high ornamental and medicinal value [22,30]. Epiphytic or lithophytic herbs commonly suffer from adverse environmental conditions such as chilling, drought, and water deficit [22,23,29,30,31,32]. Plant volatile terpenes play critical roles not only in the formation of orchid floral scents, but also in response to environmental stresses [1,2,5,6,7]. TPS is the primary enzyme responsible for catalyzing the formation of monoterpenes (C_10_), sesquiterpenes (C_15_), or diterpenes (C_20_) from the substrates GPP, FPP, or GGPP, respectively (Figure 1). Therefore, studies on floral scents have mainly focused on the identification and analysis of *TPS* genes responsible for the biosynthesis of terpenes [7,24].

Herein, 34 *DoTPS* genes were identified in the *D. officinale* genome according to the conserved *C*-terminal and *N*-terminal domain of TPS, followed by manual verification (Table 1; Figure 3). TPS subfamilies belong to a medium-sized family, with various gene numbers (approximately 20–150) among different plant species [1,10]. For example, 32 *AtTPS* genes were functionally discovered in *A. thaliana* [11]. A total of 14 *SmTPS*, 33 *OsTPS*, and 152 *VvTPS* genes were found in *S. moellendorffii*, *O. sativa*, and *V. vinifera*, respectively [1,10,17,18]. Furthermore, *TPS* occupied 0.26 genes/M in the *A. thaliana* genome (125 M) [33], 0.13 genes/M in *S. moellendorffii* (106 M) [34], 0.08 genes/M in the *O. sativa* genome (389 M) [35], 0.31 genes/M in *V. vinifera* (487 Mb) [36], and 0.02 genes/M in *D. officinale* (1.35 G) [22,23,24]. It is possible that tandem duplication may have occurred during evolution of the *D. officinale* genome, mainly in the TPS-a and TPS-b subfamilies.

Phylogenetic analysis showed that DoTPS proteins fall into four known angiosperm TPS subfamilies (TPS-a, TPS-b, TPS-c, and TPS-e/f), with the exception of the gymnosperm-specific TPS-d and *S. moellendorffii*-specific TPS-h (Figure 3). TPS-b was the largest subfamily among the DoTPS proteins, followed by TPS-a, which was consistent with *Daucus carota* [14], but in contrast to other species such as *A. thaliana* (22 of the 32 *TPS* genes were *TPS-a* genes) [12] and *S. lycopersicum* (12 of the 29 *TPS* genes were *TPS-a* genes) [17]. As illustrated in Table S12, 16 DoTPS proteins were annotated as monoterpene synthase, Fourteen DoTPS proteins were annotated as sesquiterpene synthase, and four DoTPS proteins were annotated as diterpene synthase. These four putative diterpene synthases harbored one TPS-c and three TPS-e/f proteins. TPS-e/f proteins can produce mono-, sesqui-, and di-terpenes [1]. All 14 sesquiterpene synthases and 16 monoterpene synthases were assigned to TPS-a and TPS-b, respectively (Figure 4; Table S12). For mono- and sesquiterpenes, subcellular location and availability of substrate are more important for the characterization of typical products produced in vivo. FhTPS6 was localized in the cytosol, and was deemed to be associated with the formation of a sesquiterpene (nerolidol) and several monoterpenes (myrcene, limonene, *cis*-ocimene, *trans*-ocimene, and terpinolene), suggesting that GPP and FPP might move from plastids to the cytosol [21]. TPS-a can produce monoterpenes in vitro, while TPS-b can produce hemi-, mono-, and sesquiterpenes in vitro [1]. TPS-a is an angiosperm-specific clade that is responsible for sesquiterpene or diterpene synthases, and can be further divided into monocotyledonous- and dicotyledonous-specific subgroups [1,9,10]. In *A. thaliana*, four *TPS-a* genes encode cytosolic sesquiterpene synthases, while the other nine *TPS-a* genes harbored transit peptides presumably encoding diterpene synthases, although their functions have not yet been fully investigated [12]. In *S. lycopersicum*, all *TPS-a* genes only encode sesquiterpene synthases, and 11 of 12 are cytosolic and not chloroplastic [17]. Similarly, most proteins previously functionally identified from the angiosperm-specific TPS-b clade are monoterpene synthases. For example, six *A. thaliana* TPS-b proteins clustered in the same branch that harbored AtTPS10, a monoterpene synthase that produces myrcene or ocimene [12]. These findings indicate that the TPS members share a similar functional feature within the same subfamily, however, further functional characterization is required.

The expression analysis showed that *DoTPS* genes were mainly expressed in floral organs of *D. officinale*, followed by root organs and stems (Figure 6), inferring a strict regulation of terpenoid production. Interestingly, 11 of 18 *DoTPS-b* genes with high transcript levels in floral organs were monoterpene synthase genes (Figure 6). Four monoterpene synthase genes (*DoTPS10*, *-19*, *-20*, and *-25*) were highly expressed at the semi-flowering stage (Figure 9), in agreement with the content of geraniol and linalool (Figure S2). Among them, *DoTPS10* showed the highest transcript level in floral organs (Figure 6 and Figure 9), suggesting that it may be responsible for the biosynthesis of monoterpenes. In the present study, DoTPS10 was shown to be a single-product enzyme that could covert GPP to linalool (Figure 10), the predominant component of floral scents in orchids. TPS proteins that produce the same single product have also been found in a *Freesia* hybrid [21], *Malus domestica* [16] and *Vitis vinifera* [19]. Furthermore, the majority of *DoTPS* genes could be induced by MeJA treatment, resulting in the increased production of monoterpene volatiles such as geraniol and linalool (Figure 8). The reason why exogenous MeJA resulted in the upregulation of these *DoTPS* genes may be due to the presence of G-boxes in their promoters (Table S13), which can interact with the existing CGTCA or MYC motif of the jasmonic acid signaling pathway [37], but it needs to be further explored. Therefore, activated expression of *DoTPS10* by MeJA treatment offers a critical cue for further exploring the mechanism of linalool biosynthesis in *D. officinale*.

RT-qPCR data showed that distinct tempospatial expression profiles of *DoTPS* genes could be affected when *D. officinale* was exposed to cold or osmotic treatment (Figure 7). Similarly, previous work has emphasized the importance of terpenes in defensive response to biotic attack and abiotic stresses [1,2,3,4,5,7,9,10]. It is possible that the crassulacean acid metabolism plant *D. officinale*, which adheres tightly to the surface of tree bark or rocks in locations with limited soil, thus requires TPS proteins to quickly biosynthesize terpenes to circumvent adverse environments [23,25]. To better understand the mechanisms of differential terpenoid production in *D. officinale*, more efforts should be made to integrate studies on *DoTPS* expression patterns with those on profiling of terpenes.

## 4. Materials and Methods

### 4.1. Plant Materials

*D. officinale* “Zhongke 5” (http://www.cas.cn/syky/201811/t20181109_4669776.shtml, Figure 6B) with better adaptability to adverse habitats was cultivated in a greenhouse and in the open air at the South China Botanical Garden, Chinese Academy of Sciences (Guangzhou, Guangdong Province, China) under natural light and controlled temperatures, between 25 and 30 °C. Flowers, leaves, roots, and stems from 14-month-old adult plants of full-sib *D. officinale* were sampled at the flowering stage. For osmotic treatment, 10 independent 10-month-old *D. officinale* seedlings were transferred to fresh half-strength Murashige and Skoog (MS) medium [38] supplemented with 200 mM mannitol. Control seedlings were transferred in same way without additional mannitol. For the MeJA treatment, the same seedlings were transferred to fresh half-strength MS medium supplemented with 1 mM MeJA, and MeJA-free medium was used as the control. The leaves from osmotic and MeJA treatments were collected after treatment at 0, 12, 24, and 48 h. All samples were frozen immediately in liquid nitrogen, and stored at −80 °C until use.

### 4.2. Identification of TPS Family Members in D. officinale

The recently released *D. officinale* genome [23] was used in this study. Two specific TPS domains, PF03936 and PF01397, which respectively indicate the *C*-terminal and *N*-terminal domain of TPS from the Pfam database (http://pfam.xfam.org/), were used to build the corresponding Hidden Markov Model (HMM) file. HMMER v3.3 (http://www.hmmer.org/) was used to search the *D. officinale* protein database with the PF03936 and PF01397 domains model data as queries. Significant hits (e-value < 10^−3^) were retrieved as candidate *D. officinale* TPS proteins. To verify the sequences, BLASTp (http://blast.ncbi.nlm.nih.gov) and SMART (http://smart.embl-heidelberg.de/) searches of the retrieved TPS proteins were carried out. Non-redundant sequences that did not contain the terpene synthase *C*-terminal domain and terpene synthase *N*-terminal domain were removed. The grand average of hydrophobicity (GRAVY), molecular weight (Mw), and isoelectric points (pI) of the TPS proteins were predicted from the ExPASy database (http://expasy.org/). The aliphatic index (AI), and instability index (II) were calculated by EMBOSS Pepstats tool (https://www.ebi.ac.uk/Tools/). Plant-mPLoc (www.csbio.sjtu.edu.cn/bioinf/plant-multi/), AtSubP (http://bioinfo3.noble.org/AtSubP/), and pLoc-mPlant (www.jci-bioinfo.cn/pLoc-mPlant/) were used to predict the subcellular localization of TPS proteins. The secondary structure of TPS proteins was determined using the SOPMA program (http://npsa-pbil.ibcp.fr/). In addition, TPS proteins from *P. equestris* and *A. shenzhenica* were obtained from their reported genome database [25]. The other TPS proteins from *A. grandis*, *A. thaliana*, *O. sativa*, *P. trichocarpa*, *S. moellendorffii*, *S. lycopersicum*, and *S. bicolor* were downloaded from the Phytozome version 12.1 database (https://www.phytozome.net).

### 4.3. Conserved Motifs, Gene Structure, and Phylogenetic Analysis

Conserved motifs of TPS proteins were analyzed with MEME software (http://meme-suite.org/) with default parameters. The exon–intron structure of TPS proteins was aligned with the Gene Structure Display Server (GSDS 2.0, http://gsds.cbi.pku.edu.cn/). Multiple sequence alignment was performed using TPS proteins from *A. grandis*, *A. shenzhenica*, *A. thaliana*, *D. officinale*, *O. sativa*, *P. equestris*, *P. trichocarpa*, *S. moellendorffii*, *S. lycopersicum*, and *S. bicolor* with ClustalX 2.1 software (www.clustal.org/). The alignments were manually adjusted and truncated with a focus on diagnostically conserved regions such as the DDxxD, NSE/DTE, and RRX_8_W motifs, based on a reported protocol [15]. A phylogenetic tree was constructed using the neighbor-joining (NJ) method [39] under the Jones–Taylor–Thornton (JTT) model with 1000 bootstrap replicates in MEGA version 7.0 [40]. The generated graph was redrawn and annotated by Figtree version 1.4.4 (http://tree.bio.ed.ac.uk/software/figtree/). The sequences of TPS proteins used in this study can be found in Table S6.

### 4.4. Total RNA Isolation, cDNA Reverse Transcription, and RT-qPCR Analysis

Total RNA from the flowers and leaves of 14-month-old *D. officinale* “Zhongke 5” at the flowering stage were extracted using the Quick RNA Isolation Kit (Huayueyang, Beijing, China) according to the instruction manual. Genomic DNA contamination was eliminated with RNase-free DNase I (TaKaRa, Dalian, China). First-strand cDNA was synthesized by reverse transcription with the help of the PrimeScript™ RT Reagent Kit (Takara) according to the manufacturer’s protocol. SYBR^®^ Premix Ex Taq™ (TaKaRa) was applied for RT-qPCR analysis on a LightCycler 480 instrument (Roche Diagnostics, Mannheim, Germany) as described previously [41]. *D. officinale* elongation factor 1-α (*DoEF-1α*, GenBank accession no. JF825419) was selected as the internal reference gene [42]. At least three biological replicates were carried out, and relative mRNA expression data were quantified by the 2^−ΔΔCT^ method [43]. The RT-qPCR primers of *TPS* genes listed in Table S14 were acquired by the PrimerQuest tool (http://www.idtdna.com/Primerquest/Home/Index).

### 4.5. Cis-Acting Elements Analysis of TPS Genes in D. officinale

The promoter sequences, 2000 bp upstream of the translational start site (ATG), of TPS genes in *D. officinale* were obtained from the *D. officinale* genome [23]. Afterwards, the online software PlantCARE (http://bioinformatics.psb.ugent.be/webtools/plantcare/html/) was employed to investigate putative *cis*-regulatory elements in the promoter region of *DoTPS* genes in *D. officinale*.

### 4.6. Gene Expression Analysis Based on Transcriptome Data

To gain insight into the tissue-specific transcription levels of *DoTPS* family genes, raw data from the RNA-sequencing of 10 different tissues (i.e., flower buds, green root tips, gynostemium (column), labellum (lip), leaves, pollinia, sepals, stems, roots, and the white part of roots) in two-year-old *D. officinale* adult plants was downloaded from NCBI under BioProject PRJNA262478 [25]. To study the effects of cold acclimation (0 °C for 20 h, CA) and non-acclimation (20 °C for 20 h, CK) on *DoTPS* gene expression, the raw RNA-sequencing reads were retrieved from a reported transcriptome database [29]. Fragments per kilobase of transcript per million mapped reads (FPKM) values of *DoTPS* genes in tested samples were used to evaluate transcription abundance. *DoEF-1α* was selected as the internal reference gene for normalizing each expression value. The heat maps of the *DoTPS* genes’ expression patterns were illustrated using the TBtools software with default settings (https://github.com/CJ-Chen/TBtools), and the gradient color from green to red is expressed as the log2-transformed expression levels of each *DoTPS* gene.

### 4.7. Gas Chromatography–Mass Spectrometry Analysis of Geraniol and Linalool in Flowers of D. officinale

The frozen flowers of *D. officinale* (500 mg) were ground to a fine powder in liquid nitrogen, and then blended with precooled dichloromethane (3 mL) by vortexing for 2 min, followed by shaking at 25 °C for 8 h in the dark. The supernatant was collected by 13,000× *g* centrifugation, and concentrated to 200 μL using a stream of N_2_ before analysis by gas chromatography–mass spectrometry (GC-MS, Shimadzu Co., Kyoto, Japan) equipped with a 30-m Supelcowax-10 column (0.25 mm diameter × 0.25 μm film thickness). The temperature program was isothermal at 60 °C for 3 min, then increased at a rate of 4 °C min^−1^ to 240 °C, and maintained at 240 °C for 20 min. MS analyses were performed in full-scan mode with a mass range from *m/z* 40 to 200. Geraniol and linalool were identified against the NIST 2008 mass spectra library (https://chemdata.nist.gov/) as described previously [44].

### 4.8. Prokaryotic Expression and DoTPS10 Enzyme Assay in Escherichia coli

The full-length *DoTPS10* was amplified from first-strand cDNA, as published previously [41]. The obtained PCR product was purified and inserted into the pMD-18T vector (TaKaRa) for sequencing. The gene-specific primers used for *DoTPS10* are indicated in Table S14.

A 1797-bp ORF without a stop codon (TAA) of *DoTPS10* was cloned into the pET32a vector with *Sal*I and *Xho*I restriction sites. *DoTPS10* expression in *E. coli* BL21 (DE3) cells and purification using a His-trap Ni-sepharose high performance column (GE Healthcare, Fairfield, CT, USA) were described in our previous study [45]. The purified pET32a-DoTPS10 protein was fractionated by 10% sodium dodecyl sulfate polyacrylamide gel electrophoresis (SDS-PAGE).

In vitro DoTPS10 enzyme assays were performed in screw-cap 5-mL glass vials containing 1 mL of 2-hydroxy-3-morpholinopropanesulfonic acid (MOPSO) buffer (10 mM, pH 7.0, containing 5 mM dithiothreitol, 10 mM MgCl_2_, and 10 mM GPP as substrate) and 100 µg of DoTPS10 protein. The reactions were overlaid with 200 µL of *n*-hexane and incubated at 30 °C for 1 h. The mixtures were mixed vigorously for 1 min to obtain the enzymatic products. The organic phase was removed, and 1 μL was detected using GC-MS as described above. For comparison, His-tagged protein (empty pET32a) was used as the blank control.

### 4.9. Subcellular Localization of DoTPS10 in A. thaliana Mesophyll Protoplasts

To determine the localization of DoTPS10, a 1797-bp ORF without a stop codon (TAA) of *DoTPS10* was introduced into the pSAT6-EYFP-N1 vector with an *Nco*I restriction site, and was transiently transformed into mesophyll protoplasts from four-week-old *A. thaliana* leaves. After transformation in darkness at 22 °C for 20 h, YFP signals were evaluated using a Zeiss LSM 510 Meta confocal microscope (Carl Zeiss, Jena, Germany) with an excitation wavelength of 514 nm.

### 4.10. Statistical Analysis

IBM SPSS statistics software version 22.0 for Windows (IBM Corp., Armonk, NY, USA) was used to carry out one-way analysis of variance (ANOVA) among different samples using three replications. Duncan’s multiple range test (DMRT) was used to determine significant differences (*p* < 0.05).

## 5. Conclusions

In this study, we reported on the identification of 34 *DoTPS* genes in *D. officinale*. Their conserved motifs, exon–intron distribution, and phylogenetic analysis was assessed. Differential expression patterns of *DoTPS* genes exposed to ten different organs and three flowering stages, highlights their involvement in regulating the biosynthesis of floral monoterpenes, as well as the responses of plants to exogenous MeJA treatment, cold, and osmotic stress. One monoterpene synthase (DoTPS10), which was targeted to chloroplasts, could specifically convert GPP into linalool in vitro. Our findings show that transcript accumulation of multiple *TPS* genes is mainly responsible for the formation of floral terpenes, and provides a foundation for further studies on orchid floral scent research through the regulation of *DoTPS* genes.

## Figures and Tables

**Table 1:** Information of the plant TPS gene family in *D. officinale*.

Name	Gene ID ^1^	ORF ^2^ (bp)	AA ^3^ (aa)	pI ^4^	Mw ^5^ (kDa)	AI ^6^	II ^7^	GRAVY ^8^	Localization ^9^
*DoTPS1*	Dca014928	960	319	6.31	36.82	90.78	48.81	−0.342	Chloroplast ^a,b,c^
*DoTPS2*	Dca000724	1902	633	6.47	74.02	88.44	43.11	−0.260	Chloroplast ^a,b,c^
*DoTPS3*	Dca000725	1827	608	5.46	70.52	89.67	41.24	−0.231	Chloroplast ^a,b,c^
*DoTPS4*	Dca022838	2571	856	7.18	100.05	80.35	47.78	−0.429	Chloroplast ^a,b,c^
*DoTPS5*	Dca003141	1692	563	5.11	65.84	98.86	42.13	−0.228	Chloroplast ^a,b^/Cytoplasm ^c^
*DoTPS6*	Dca019411	1521	506	5.13	59.48	93.68	43.71	−0.133	Chloroplast ^a,b^/Cytoplasm ^c^
*DoTPS7*	Dca003139	1692	563	5.67	65.72	92.49	39.06	−0.266	Chloroplast ^a,b^/Cytoplasm ^c^
*DoTPS8*	Dca028160	579	192	6.83	22.63	100.62	45.31	−0.121	Chloroplast ^a^/Unknown ^b^/Cytoplasm ^c^
*DoTPS9*	Dca019412	1665	554	5.03	64.93	92.94	39.76	−0.189	Chloroplast ^a,b^/Cytoplasm ^c^
*DoTPS10*	Dca007746	1797	598	5.73	69.73	93.61	47.41	−0.242	Chloroplast ^a,b^/Cytoplasm ^c^
*DoTPS11*	Dca022749	696	231	5.13	27.40	104.20	49.98	−0.045	Chloroplast ^a,b^/Cytoplasm ^c^
*DoTPS12*	Dca024936	378	125	5.64	14.98	98.32	40.18	−0.326	Chloroplast ^a^/Cytoplasm ^b,c^
*DoTPS13*	Dca026570	1659	552	5.59	64.94	96.97	38.16	−0.266	Chloroplast ^a,b,c^
*DoTPS14*	Dca005188	2550	849	6.71	98.69	86.21	46.66	−0.352	Chloroplast ^a,b,c^
*DoTPS15*	Dca025698	1659	552	5.31	64.82	89.60	44.73	−0.284	Chloroplast ^a,b,c^
*DoTPS16*	Dca016979	1650	549	5.62	64.23	91.62	36.94	−0.374	Chloroplast ^a,b^/Cytoplasm ^c^
*DoTPS17*	Dca008309	1653	550	5.42	64.89	97.13	47.01	−0.233	Chloroplast ^a,b,c^
*DoTPS18*	Dca011215	1674	557	5.36	64.80	95.10	44.24	−0.302	Chloroplast ^a,b,c^
*DoTPS19*	Dca010855	1446	481	4.94	55.95	110.46	34.63	0.013	Chloroplast ^a,b,c^
*DoTPS20*	Dca026890	1749	582	5.20	68.07	92.84	33.82	−0.295	Chloroplast ^a,b,c^
*DoTPS21*	Dca007747	1797	598	5.62	69.60	94.92	48.15	−0.224	Chloroplast ^a,b^/Cytoplasm ^c^
*DoTPS22*	Dca003142	1692	563	5.24	65.61	95.26	43.73	−0.245	Chloroplast ^a,b^/Cytoplasm ^c^
*DoTPS23*	Dca011214	1674	557	5.22	65.03	90.36	37.30	−0.331	Chloroplast ^a,b^/Cytoplasm ^c^
*DoTPS24*	Dca000728	1386	461	6.38	53.78	93.08	38.16	−0.180	Chloroplast ^a,b^/Cytoplasm ^c^
*DoTPS25*	Dca013782	1794	597	5.31	69.67	94.61	44.76	−0.274	Chloroplast ^a,b^/Cytoplasm ^c^
*DoTPS26*	Dca026369	1650	549	5.42	64.61	90.73	42.39	−0.438	Chloroplast ^a,b^/Cytoplasm ^c^
*DoTPS27*	Dca000723	1938	645	5.89	74.89	91.74	48.68	−0.240	Chloroplast ^a,b^/Cytoplasm ^c^
*DoTPS28*	Dca003295	1863	620	5.91	72.57	92.35	47.89	−0.262	Chloroplast ^a,b^/Cytoplasm ^c^
*DoTPS29*	Dca018407	1653	550	5.57	64.68	95.89	50.10	−0.251	Chloroplast ^a,b^/Cytoplasm ^c^
*DoTPS30*	Dca013784	1377	458	5.07	53.35	93.52	38.64	−0.254	Chloroplast ^a,b^
*DoTPS31*	Dca016966	1089	362	7.07	41.65	99.70	51.56	−0.193	Chloroplast ^a,b,c^
*DoTPS32*	Dca018946	2433	810	5.75	91.13	88.99	46.05	−0.169	Chloroplast ^a,b,c^
*DoTPS33*	Dca017971	1536	511	5.57	58.70	97.18	42.66	−0.109	Chloroplast ^a,b^/Cytoplasm ^c^
*DoTPS34*	Dca020940	1797	598	5.19	69.59	91.52	45.72	−0.280	Chloroplast ^a,b,c^

^1^ Gene ID, it is annotated in *D. officinale* genome [23]; ^2^ ORF, open reading frame; ^3^ AA, amino acid; ^4^ pI, theoretical isoelectric point; ^5^ Mw, molecular weight; ^6^ AI, aliphatic index; ^7^ II, instability index; ^8^ GRAVY, grand average of hydrophobicity; ^9^ Localization is predicted by Plant-mPLoc [27] (Table S1, http://www.csbio.sjtu.edu.cn/bioinf/plant-multi/), AtSubP [26] (Table S2, http://bioinfo3.noble.org/AtSubP/), and pLoc-mPlant [28] (Table S3, http://www.jci-bioinfo.cn/pLoc-mPlant/) tools. ^a, b, c^ indicates the result of Plant-mPLoc, AtSubP, and pLoc-mPlant, respectively.

## Supplementary Materials

Supplementary materials can be found at https://www.mdpi.com/1422-0067/21/15/5419/s1.

## Abbreviations

AI	Aliphatic index
bp	Base pair
CA	Cold acclimation
DMAPP	Dimethylallyl diphosphate
FPP	Farnesyl diphosphate
FPKM	Fragments per kilobase of transcript per million fragments mapped
GC–MS	Gas chromatography-mass spectrometry
GGPP	Geranylgeranyl diphosphate
GPP	Geranyl diphosphate
GRAVY	Grand average of hydrophobicity
II	Instability index
IPP	Isopentenyl diphosphate
MeJA	Methyl jasmonate
MEP	Methylerythritol phosphate
MS	Murashige and Skoog medium
Mw	Molecular weight
MVA	Mevalonic acid
NCBI	National Center for Biotechnology Information
NJ	Neighbor-joining
ORF	Open reading frame
pI	Isoelectric point
RT-qPCR	Real-time reverse transcription quantitative polymerase chain reaction
SDS-PAGE	Sodium dodecyl sulfate polyacrylamide gel electrophoresis
TPS	Terpene synthase
YFP	Yellow fluorescent protein
