# Supplementary material for: Genome-Wide Identification and Expression Profile of TPS Gene Family in Dendrobium officinale and the Role of DoTPS10 in Linalool Biosynthesis
Source: Int J Mol Sci. 2020 Jul 30;21(15):5419. doi: 10.3390/ijms21155419 (PMC7432446; doi:10.3390/ijms21155419)
Supplement: Supplementary file 1 [file ijms-21-05419-s001.pdf]

SUPPLEMENTARY MATERIALS

Genome-wide identification and expression profile of *TPS* gene family in *Dendrobium officinale* and the role of *DoTPS10* in linalool biosynthesis

Zhenming Yu [1,2,†], Conghui Zhao [1,3,†], Guihua Zhang [1], Jaime A. Teixeira da Silva [4], Jun Duan [1,2,*]

1 Key Laboratory of South China Agricultural Plant Molecular Analysis and Genetic Improvement & Guangdong Provincial Key Laboratory of Applied Botany, South China Botanical Garden, Chinese Academy of Sciences, Guangzhou 510650, China; zhenming311@scbg.ac.cn (Z.Y.); zhaoconghui@scbg.ac.cn (C.Z.) ; zhanggh@scbg.ac.cn (G.Z.)

2 Center of Economic Botany, Core Botanical Gardens, Chinese Academy of Sciences, Guangzhou 510650, China

3 University of Chinese Academy of Sciences, No. 19A Yuquan Road, Beijing 100049, China

4 Independent researcher, P. O. Box 7, Miki-cho post office, Ikenobe 3011-2, Kagawa-ken 761-0799, Japan; jaimetex@yahoo.com

* Correspondence: duanj@scib.ac.cn; Tel.: +86-020-37252978

† These authors contributed equally to this work.

Supplementary table legends

Table S1 Localization was predicted by Plant-mPLoc (http://www.csbio.sjtu.edu.cn/bioinf/plant-multi/).

Table S2 Localization was predicted by AtSubP tool (http://bioinfo3.noble.org/AtSubP/index.php).

Table S3 Localization was predicted by pLoc-mPlant (http://www.jci-bioinfo.cn/pLoc-mPlant/).

Table S4 Secondary structure of DoTPS proteins.

Table S5 Distribution of conserved motifs in *D. officinale* TPS proteins based on the results of MEME analysis.

Table S6 TPS proteins represented in phylogenetic analysis. TPS proteins from *P. equestris* and *A. shenzhenica* were obtained from their genome databases [25]. The other TPS proteins were downloaded from Phytozome version 12.1 database (http://phytozome.jgi.doe.gov/pz/portal.html).

Table S7 The FPKM values of *DoTPS* genes in different *D. officinale* organs.

Table S8 The expression values of *DoTPS* genes in response to 200 mM mannitol treatment for 48 h.

Table S9 The FPKM values of *DoTPS* genes exposed to cold treatment (0°C) for 20 h.

Table S10 The expression values of *DoTPS* genes in response to 200 mM mannitol treatment for 48 h.

Table S11 The expression values of *DoTPS* genes in at three flowering stages of *D. officinale*.

Table S12 Functional annotation of DoTPS with Terzyme (http://www.nipgr.ac.in/terzyme.html).

Table S13 CGTCA-motif, G-box and MYC motif in the promoter region of *DoTPS* genes.

Table S14 Primers used for RT-qPCR analysis, DoTPS10 cloning and its pET32a and YFP vector construction. Gene-specific primers for real-time reverse transcription quantitative PCR (RT-qPCR) were designed by the PrimerQuest tool (http://www.idtdna.com/Primerquest/Home/Index). The *D. officinale* actin gene (*DoEF-1α*) was obtained from NCBI (GenBank accession no. JF825419). F, forward; R, reverse.

Supplementary figure legends

Figure S1 Distribution of conserved motifs in *D. officinale* TPS proteins based on the results of MEME analysis.

Figure S2 Content of geraniol and linalool in *D. officinale* at three flowering stages: budding (B), semi-flowering (S) and full flowering (F). Each bar represents the mean (± standard error, n = 3) of three independent biological replicates. Different letters above the bars indicate significant differences ($p < 0.05$, Duncan's multiple range test).

Figure S3 Gas chromatograms of products yielded by DoTPS10 using GPP as substrate. Each bar represents the mean (± standard error, n = 3) of three independent biological replicates. Different letters above the bars indicate significant differences ($p < 0.05$, Duncan's multiple range test).

Table S1 Localization was predicted by Plant-mPLoc (http://www.csbio.sjtu.edu.cn/bioinf/plant-multi/).

Protein	Subcellular localization
DoTPS1	Chloroplast
DoTPS2	Chloroplast
DoTPS3	Chloroplast
DoTPS4	Chloroplast
DoTPS5	Chloroplast
DoTPS6	Chloroplast
DoTPS7	Chloroplast
DoTPS8	Chloroplast
DoTPS9	Chloroplast
DoTPS10	Chloroplast
DoTPS11	Chloroplast
DoTPS12	Chloroplast
DoTPS13	Chloroplast
DoTPS14	Chloroplast
DoTPS15	Chloroplast
DoTPS16	Chloroplast
DoTPS17	Chloroplast
DoTPS18	Chloroplast
DoTPS19	Chloroplast
DoTPS20	Chloroplast
DoTPS21	Chloroplast
DoTPS22	Chloroplast
DoTPS23	Chloroplast
DoTPS24	Chloroplast
DoTPS25	Chloroplast
DoTPS26	Chloroplast
DoTPS27	Chloroplast
DoTPS28	Chloroplast
DoTPS29	Chloroplast
DoTPS30	Chloroplast
DoTPS31	Chloroplast
DoTPS32	Chloroplast
DoTPS33	Chloroplast
DoTPS34	Chloroplast

Table S2 Localization was predicted by AtSubP tool (http://bioinfo3.noble.org/AtSubP/index.php).

Protein	Chloro	Cyto	Golgi	Mito	Extracl	Nucl	Celmemb	Prediction
DOTPS1	0.41	-1.25	-1.29	-0.43	-1.04	-1.03	-1.25	Chloroplast
DOTPS2	0.86	-1.14	-1.29	-0.56	-1.06	-1.28	-1.27	Chloroplast
DOTPS3	1.22	-1.29	-1.29	-1.05	-1.06	-1.22	-1.19	Chloroplast
DOTPS4	1.06	-1.25	-1.29	-0.84	-1.08	-1.17	-1.35	Chloroplast
DOTPS5	1.40	-0.99	-1.29	-1.59	-1.04	-1.00	-1.36	Chloroplast
DOTPS6	0.70	-1.11	-1.29	-0.51	-1.04	-0.96	-1.38	Chloroplast
DOTPS7	1.36	-0.75	-1.29	-1.47	-1.06	-1.01	-1.40	Chloroplast
DOTPS8	-1.13	-0.92	-1.29	-0.76	-0.73	-0.48	-0.42	Unknown
DOTPS9	1.82	-1.18	-1.29	-1.62	-1.08	-1.21	-1.37	Chloroplast
DOTPS10	1.20	-1.42	-1.29	-0.93	-1.06	-1.19	-1.35	Chloroplast
DOTPS11	1.35	-0.80	-1.29	-1.21	-1.07	-1.34	-1.08	Chloroplast
DOTPS12	-1.24	1.09	-1.29	-1.74	-0.95	-1.08	-1.22	Cytoplasm
DOTPS13	1.29	-0.68	-1.29	-1.68	-1.06	-1.04	-1.14	Chloroplast
DOTPS14	1.01	-0.98	-1.29	-1.37	-1.05	-1.08	-1.07	Chloroplast
DOTPS15	1.85	-1.01	-1.29	-1.83	-1.10	-1.33	-1.17	Chloroplast
DOTPS16	1.41	-0.96	-1.29	-1.43	-1.06	-1.11	-1.10	Chloroplast
DOTPS17	1.03	-0.70	-1.29	-1.12	-1.06	-0.97	-1.26	Chloroplast
DOTPS18	1.32	-0.99	-1.29	-1.52	-1.05	-1.15	-1.19	Chloroplast
DOTPS19	0.90	-1.04	-1.29	-0.87	-1.02	-0.99	-1.22	Chloroplast
DOTPS20	0.40	-1.32	-1.29	0.00	-1.04	-1.12	-1.40	Chloroplast
DOTPS21	1.19	-1.38	-1.29	-0.97	-1.05	-1.19	-1.28	Chloroplast
DOTPS22	1.54	-0.98	-1.29	-1.52	-1.06	-1.14	-1.44	Chloroplast
DOTPS23	1.50	-1.03	-1.29	-1.59	-1.06	-1.21	-1.21	Chloroplast
DOTPS24	0.54	-0.96	-1.29	-0.73	-1.00	-1.04	-1.07	Chloroplast
DOTPS25	1.13	-1.39	-1.29	-1.23	-1.00	-1.06	-1.27	Chloroplast
DOTPS26	1.21	-0.65	-1.29	-1.47	-1.08	-0.96	-1.21	Chloroplast
DOTPS27	1.44	-1.40	-1.29	-1.33	-1.07	-1.27	-1.15	Chloroplast
DOTPS28	1.28	-1.44	-1.29	-1.19	-1.02	-1.11	-1.28	Chloroplast
DOTPS29	1.33	-1.04	-1.29	-1.06	-1.10	-1.17	-1.33	Chloroplast
DOTPS30	0.87	-1.25	-1.29	-1.08	-1.03	-1.18	-1.28	Chloroplast
DOTPS31	1.49	-1.34	-1.29	-1.32	-1.09	-1.32	-1.28	Chloroplast
DOTPS32	1.15	-1.28	-1.29	-1.05	-1.09	-1.20	-1.24	Chloroplast
DOTPS33	0.77	-1.15	-1.29	-0.72	-1.04	-1.15	-1.15	Chloroplast
DOTPS34	1.50	-1.56	-1.29	-1.30	-1.06	-1.22	-1.35	Chloroplast

Prediction approach followed the best hybrid-based classifier (AA+PSSM+N-Center-C+PSI-BLAST). Chloro, chloroplast; Cyto, cytoplasm; Mito, mitochondrion; Extracl, extracellular; Nucl, nucleus; Celmemb, cell membrane.

Table S3 Localization was predicted by pLoc-mPlant (http://www.jci-bioinfo.cn/pLoc-mPlant/).

Protein	Subcellular localization
DoTPS1	Cytoplasm
DoTPS2	Chloroplast
DoTPS3	Chloroplast
DoTPS4	Chloroplast
DoTPS5	Cytoplasm
DoTPS6	Cytoplasm
DoTPS7	Cytoplasm
DoTPS8	Cytoplasm
DoTPS9	Cytoplasm
DoTPS10	Cytoplasm
DoTPS11	Cytoplasm
DoTPS12	Cytoplasm
DoTPS13	Chloroplast
DoTPS14	Chloroplast
DoTPS15	Chloroplast
DoTPS16	Cytoplasm
DoTPS17	Chloroplast
DoTPS18	Chloroplast
DoTPS19	Chloroplast
DoTPS20	Chloroplast
DoTPS21	Cytoplasm
DoTPS22	Cytoplasm
DoTPS23	Cytoplasm
DoTPS24	Cytoplasm
DoTPS25	Cytoplasm
DoTPS26	Cytoplasm
DoTPS27	Cytoplasm
DoTPS28	Cytoplasm
DoTPS29	Cytoplasm
DoTPS30	Chloroplast
DoTPS31	Chloroplast
DoTPS32	Chloroplast
DoTPS33	Cytoplasm
DoTPS34	Chloroplast

Table S4 Secondary structure of DoTPS proteins.

	Alpha helix (%)	Extended strand (%)	Beta turn (%)	Random coil (%)
DoTPS1	61.44	5.02	4.39	29.15
DoTPS2	64.45	5.21	3.48	26.86
DoTPS3	68.26	3.29	3.29	25.16
DoTPS4	57.71	6.19	3.50	32.59
DoTPS5	69.09	4.80	3.02	23.09
DoTPS6	74.51	2.96	3.75	18.77
DoTPS7	69.98	4.44	3.73	21.85
DoTPS8	76.56	3.65	0.00	19.79
DoTPS9	70.04	3.61	2.89	23.47
DoTPS10	66.72	3.18	3.18	26.92
DoTPS11	80.95	1.30	1.73	16.02
DoTPS12	79.20	12.80	1.60	6.40
DoTPS13	69.20	3.99	2.36	24.46
DoTPS14	57.71	7.42	3.30	31.57
DoTPS15	70.65	3.80	3.08	22.46
DoTPS16	71.77	3.10	3.10	22.04
DoTPS17	69.82	3.27	3.09	23.82
DoTPS18	70.38	2.33	3.23	24.06
DoTPS19	74.84	2.29	3.95	18.92
DoTPS20	69.24	3.61	3.78	23.37
DoTPS21	63.88	4.01	3.01	29.10
DoTPS22	69.27	4.09	3.37	23.27
DoTPS23	71.10	1.97	3.59	23.34
DoTPS24	64.64	5.86	2.17	27.33
DoTPS25	64.99	5.36	3.02	26.63
DoTPS26	71.04	4.01	3.46	21.49
DoTPS27	66.98	4.19	2.95	25.89
DoTPS28	64.35	5.16	3.06	27.42
DoTPS29	69.09	4.73	3.64	22.55
DoTPS30	72.49	2.84	3.49	21.18
DoTPS31	62.15	5.52	3.59	28.73
DoTPS32	64.07	5.80	4.07	26.05
DoTPS33	72.80	2.74	4.31	20.16
DoTPS34	65.72	4.68	3.68	25.92

Table S5 Distribution of conserved motifs in *D. officinale* TPS proteins based on the results of MEME analysis.

Name	Sequences	E-value	Sites	Width
Motif 1	EPHYSRARKEITKVISLITTJDDIYDVYGTLEELZLFTBAI	2.3×e^{-657}	31	41
Motif 2	BVKGLLSLYEASYLAMPGEDILDEAKEFAKKHLK	2.7×e^{-503}	31	34
Motif 3	KREWKDLCKAYLVEAKWYNNGYIPTLDEYLENAWISIAYPL	2.8×e^{-468}	28	41
Motif 4	FYPPIIKYSCIIFRLYBDJGTSEFEQKRGDVAKSIQCYMKE	1.0×e^{-474}	23	41
Motif 5	NPILLEFAKLDFNMVQSIHQEELKEJSRWWR	6.4×e^{-463}	31	31
Motif 6	EISHALELPLHRRMDRLHARWFI	9.1×e^{-331}	33	23
Motif 7	WKIDAVQQLPEYMKICLLALFNTMNDIANELSKEKGLDILP	2.4×e^{-369}	26	41
Motif 8	EQLELIDSJQRLGVAYHFEEEIKBLLSKJ	6.1×e^{-354}	30	29
Motif 9	SSKPARRSANYQPSVWDDSFIQSLPLPSM	5.9×e^{-240}	22	29
Motif 10	ALLFRLLRZHGYHIS	5.5×e^{-212}	32	15
Motif 11	ELPFARDRLVENYFWALGVYF	1.7×e^{-262}	29	21
Motif 12	EARDKJREMIEKYWKILNREY	9.8×e^{-179}	24	21
Motif 13	FYQYGDGYTEPDHETKDQIISVLIEPIPL	5.1×e^{-199}	21	29
Motif 14	TCASYIGMQEVISKEVFDWVT	1.3×e^{-101}	11	21
Motif 15	LFSSFKDEKGSFKPC	3.6×e^{-93}	24	15
Motif 16	EKQKKRREKLKEEVRKLJDDT	3.1×e^{-89}	23	21
Motif 17	NSNYEESFKKVLIBJPRTAQC	8.0×e^{-71}	12	21
Motif 18	SNLYLSTDFVHNYGFFLPLKIQHSAQSFSSTPYLRLRKKCYSVASN	8.4×e^{-73}	4	46
Motif 19	VLTAAYCLSEDLTKEALNSLE	1.2×e^{-69}	13	21
Motif 20	PVSAYETAWVAMVPDPDDPTSPMFPEYLDWILRNQNBLGFWFDQQHG	8.3×e^{-39}	3	47

Table S6 TPS proteins represented in phylogenetic analysis

Species	Sequence
Abies grandis	>AAF61453
	MALVSSAPKSCLHKSLIRSTHHELKPLRRTIPTLGMCRRGKSFTPSVSMSLTTAVSDDGLQRRIGDYHSNLWDDDFIQSLSTPYGEPSYR
	ERAEKLIGEVKEMFNSMPSEDGESMSPLNDLIERLWMVDSVERLGIDRHFKKEIKSALDYVYSYWNEKGIGCGRDSVFPDVNSTASGF
	RTLRLHGYSVSSEVLKVFQDQNGQFAFSPSTKERDIRTVLNLYRASFIAFPGEKVMEEAEIFSSRYLKEAVQKIPVSSLSQEIDYTLEYGW
	HTNMPRLETRNYLDVFGHPTSPWLKKKRTQYLDSEKLLELAKLEFNIFHSLQQKELQYLSRWWIHSGLPELTFGRHRHVEYYTLSSCI
	ATEPKHSAFRLGFAKTCHLITVLDDIYDTFGTMDEIELFNEAVRRWNPSEKERLPEYMKEIYMALYEALTDMAREAEKTQGRDTLNY
	ARKAWEVYLDSYTQEAKWIASGYLPTFEEYLENAKVSSGHRAAALTPLLTLDVPLPDDVLKGIDFPSRFNDLASSFLRLRGDTRCYKA
	DRDRGEEASSISCYMKDNPGLTEEDALNHINAMINDIIKELNWELLKPDSNIPMTARKHAYEITRAFHQLYKYRDGFSVATQETKSLV
	RRTVLEPVPL
	>AAB71084
	MALVSISPLASKSCLRKSLISSIHEHKPPYRTIPNLGMRRRGKSVTPSMSISLATAAPDDGVQRRIGDYHSNIWDDDFIQSLSTPYGEPSY
	QERAERLIVEVKKIFNSMYLDDGRLMSSFNDLMQRLWIVDSVERLGIARHFKNEITSALDYVFRYWEENGIGCGRDSIVTDLNSTALGF
	RTLRLHGYTVSPEVLKAFQDQNGQFVCSPGQTEGEIRSVLNLYRASLIAFPGEKVMEEAEIFSTRYLKEALQKIPVSALSQEIKFVMEYG
	WHTNLPRLEARNYIDTLEKDTSAWLNKNAGKKLLELAKLEFNIFNSLQQKELQYLLRWWKESDLPKLTFARHRHVEFYTLASCIAID
	PKHSAFRLGFAKMCHLVTVLDDIYDTFGTIDELELFTSAIKRWNSSEIEHLPEYMKCVYMVVFETVNELTREAEKTQGRNTLNYVRKA
	WEAYFDSYMEEAKWISNGYLPMFEEYHENGKVSSAYRVATLQPILTLNAWLPDYILKGIDFPSRFNDLASSFLRLRGDTRCYKADRDR
	GEEASCISCYMKDNPGSTEEDALNHINAMVNDIIKELNWELLRSNDNIPMLAKKHAFDITRALHHLYIYRDGFSVANKETKKLVMET
	LLESMLF
	>AAF61455
	MALLSIVSLQVPKSCGLKSLISSSNVQKALCISTAVPTLRMRRRQKALVINMKLTTVSHRDDNGGGVLQRRIADHHPNLWEDDFIQSL
	SSPYGGSSYSERAVTVVEEVKEMFNSIPNNRELFGSQNDLLTRLWMVDSIERLGIDRHFQNEIRVALDYVYSYWKEKEGIGCGRDSTFP
	DLNSTALALRTLRLHGYNVSSDVLEYFKDQKGHFACPAILTEGQITRSVLNLYRASLVAFPGEKVMEEAEIFSASYLKEVLQKIPVSSFS
	REIEYVLEYGWHTNLPRLEARNYIDVYGQDSYESSNEMPYVNTQKLLKLAKLEFNIFHSLQQKELQYISRWWKDSCSSHLTFTRHRHV
	EYYTMASCISMEPKHSAFRLGFVKTCHLLTVLDDMYDTFGTLDELQLFTTAFKRWDLSETKCLPEYMKAVYMDLYQCLNELAQEAE

KTQGRDTLNYIRNAYESHFDSFMHEAKWISSGYLPTFEEYLKNGKVSSGSRTATLQPILTLDVPLPNYILQEIDYPSRFNDLASSLLRLR
GDTRCYKADRARGEEASAISCYMKDHPGSTEEDALNHINVMISDAIRELNWELLRPDSKSPISSKKHAFDITRAFHHLYKYRDGYTVA
SSETKNLVMKTVLEPVAL
>AAF61454
MALVSILPLSSKSVLHKSWIVSTYEHKAISRTIPNLGLRGRGKSVTHSLRMSLSTAVSDDHGVQRRIVEFHSNLWDDDFIQSLSTPYGAP
SYRERADRLIVEVKGIFTSISAEDGELITPLNDLIQRLLMVDNVERLGIDRHFKNEIKAALDYVYSYWNEKGIGSGSDSGVADLNSTALG
FRILRLHGYSVSSDVLEHFKEEKEKGQFVCSAIQTEEEIKSVLNLFRASLIAFPGEKVMEEAEIFSKIYLKEALQNIAVSSLSREIEYVLEDG
WQTNMPRLETRNYIDVLGENDRDETLYMNMEKLLEIAKLEFNIFHSLQQRELKDLSRWWKDSGFSHLTFSRHRHVEFYALASCIETD
RKHSGFRLGFAKMCHLITVLDDIYDTFGTMEELELFTAAFKRWDPSATDLLPEYMKGLYMVVYETVNEIAREADKSQGRETLNDARR
AWEAYLDSYMKEAEWISSGYLPTFEEYMETSKVSFGYRIFALQPILTMDVPLTHHILQEIDFPLRFNDLICSILRLKNDTRCYKADRARG
EEASCISCYMKENPGSTEEDAINHINAMVNNLIKEVNWELLRQDGTAHIACKKHAFDILKGSLHGYKYRDGFSVANKETKNWVRRT
VLESVPL
>AAB70907
MALLSIVSLQVPKSCGLKSLISSSNVQKALCISTAVPTLRMRRRQKALVINMKLTTVSHRDDNGGGVLQRRIADHHPNLWEDDFIQSL
SSPYGGSSYSERAETVVEEVKEMFNSIPNNRELFGSQNDLLTRLWMVDSIERLGIDRHFQNEIRVALDYVYSYWKEKEGIGCGRDSTFP
DLNSTALALRTLRLHGYNVSSDVLEYFKDEKGHFACPAILTEGQITRSVLNLYRASLVAFPGEKVMEEAEIFSASYLKKVLQKIPVSNLS
GEIEYVLEYGWHTNLPRLEARNYIEVYEQSGYESLNEMPYMNMKKLLQLAKLEFNIFHSLQLRELQSISRWWKESGSSQLTFTRHRHV
EYYTMASCISMLPKHSAFRMEFVKVCHLVTVLDDIYDTFGTMNELQLFTDAIKRWDLSTTRWLPEYMKGVYMDLYQCINEMVEEAE
KTQGRDMLNYIQNAWEALFDTFMQEAKWISSSYLPTFEEYLKNAKVSSGSRIATLQPILTLDVPLPDYILQEIDYPSRFNELASSILRLRG
DTRCYKADRARGEEASAISCYMKDHPGSIEEDALNHINAMISDAIRELNWELLRPDSKSPISSKKHAFDITRAFHHVYKYRDGYTVSN
NETKNLVMKTVLEPLAL
>AAB71085
MALVSTAPLASKSCLHKSLISSTHELKALSRTIPALGMSRRGKSITPSISMSSTTVVTDDGVRRRMGDFHSNLWDDDVIQSLPTAYEEKS
YLERAEKLIGEVKNMFNSMSLEDGELMSPLNDLIQRLWIVDSLERLGIHRHFKDEIKSALDYVYSYWGENGIGCGRESVVTDLNSTAL
GLRTLRLHGYPVSSDVFKAFKGQNGQFSCSENIQTDEEIRGVLNLFRASLIAFPGEKIMDEAEIFSTKYLKEALQKIPVSSLSREIGDVLEY
GWHTYLPRLEARNYIQVFGQDTENTKSYVKSKKLLELAKLEFNIFQSLQKRELESLVRWWKESGFPEMTFCRHRHVEYYTLASCIAFE

PQHSGFRLGFAKTCHLITVLDDMYDTFGTVDELELFTATMKRWDPSSIDCLPEYMKGVYIAVYDTVNEMAREAEEAQGRDTLTYARE
AWEAYIDSYMQEARWIATGYLPSFDEYYENGKVSCGHRISALQPILTMDIPFPDHILKEVDFPSKLNDLACAILRLRGDTRCYKADRA
RGEEASSISCYMKDNPGVSEEDALDHINAMISDVIKGLNWELLKPDINVPISAKKHAFDIARAFHYGYKYRDGYSVANVETKSLVTRT
LLESVPL
>AAB70707
MALLSITPLVSRSCLSSSHEIKALRRTIPTLGICRPGKSVAHSINMCLTSVASTDSVQRRVGNYHSNLWDDDFIQSLISTPYGAPDYRERA
DRLIGEVKDIMFNFKSLEDGGNDLLQRLLLVDDVERLGIDRHFKKEIKTALDYVNSYWNEKGIGCGRESVVTDLNSTALGLRTLRLHG
YTVSSDVLNVFKDKNGQFSSTANIQIEGEIRGVLNLFRASLVAFPGEKVMDEAETFSTKYLREALQKIPASSILSLEIRDVLEYGWHTNL
PRLEARNYMDVFGQHTKNKNAAEKLLELAKLEFNIFHSLQERELKHVSRWWKDSGSPEMTFCRHRHVEYYALASCIAFEPQHSGFR
LGFTKMSHLITVLDDMYDVFGTVDELELFTATIKRWDPSAMECLPEYMKGVYMMVYHTVNEMARVAEKAQGRDTLNYARQAWE
ACFDSYMQEAKWIATGYLPTFEEYLENGKVSSAHRPCALQPILTLDIPFPDHILKEVDFPSKLNDLICIILRLRGDTRCYKADRARGEEA
SSISCYMKDNPGLTEEDALNHINFMIRDAIRELNWELLKPDNSVPITSKKHAFDISRVWHHGYRYRDGYSFANVETKSLVMRTVIEPV
PL
>AAC05727
MAEISESSIPRRTGNHHGNVWDDDLIHSLNSPYGAPAYYELLQKLIQEIKHLLLTEMEMDDGDHDLIKRLQIVDTLECLGIDRHFEHEI
QTAALDYVYRWWNEKGIGEGSRDSFSKDLNATALGFRALRLHRYNVSSGVLKNFKDENGKFFCNFTGEEGRGDKQVRSMLSLLRAS
EISFPGEKVMEEAKAFTREYLNQVLAGHGDVTDVDQSLLREVKYALEFPWHCSVPRWEARSFLEIYGHNHSWLKSNINQKMLKLAK
LDFNILQCKHHKEIQFITRWWRDSGISQLNFYRKRHVEYYSWVVMCIFEPEFSESRIAFAKTAILCTVLDDLYDTHATLHEIKIMTEGVR
RWDLSLTDDLPDYIKIAFQFFFNTVNELIVEIVKRQGRDMTTIVKDCWKRYIESYLQEAEWIATGHIPTFNEYIKNGMASSGMCILNLN
PLLLLDKLLPDNILEQIHSPSKILDLLELTGRIADDLKDFEDEKERGEMASSLQCYMKENPESTVENALNHIKGILNRSLEEFNWEFMK
QDSVPMCCKKFTFNIGRGLQFIYKYRDGLYISDKEVKDQIFKILVHQVPMEE
>AAC05728
MAQISESVSPSTDLKSTESSITSNRHGNMWEDDRIQSLNSPYGAPAYQERSEKLIEEIKLLFLSDMDDSCNDSDRDLIKRLEIVDTVECLG
IDRHFQPEIKLALDYVYRCWNERGIGEGSRDSLKKDLNATALGFRALRLHRYNVSSGVLENFRDDNGQFFCGSTVEEEGAEAYNKHV
RCMLSLSRASNILFPGEKVMEEAKAFTTNYLKKVLAGREATHVDESLLGEVKYALEFPWHCSVQRWEARSFIEIFGQIDSELKSNLSKK
MLELAKLDFNILQCTHQKELQIISRWFADSSIASLNFYRKCYVEFYFWMAAAISEPEFSGSRVAFTKIAILMTMLDDLYDTHGTLDQLK

	IFTEGVRRWDVSLVEGLPDFMKIAFEFWLKTSNELIAEAVKAQGQDMAAYIRKNAWERYLEAYLQDAEWIATGHVPTFDEYLNNGT<br>PNTGMCVLNLIPLLLMGEHLPIDILEQIFLPSRFHHLIELASRLVDDARDFQAEKDHGDLSCIECYLKDHPESTVEDALNHVNGLLGN<br>CLLEMNWKFLKKQDSVPLSCKKYSFHVLARSIQFMYNQGDGFSISNKVIKDQVQKVLIVPVPI<br>>AAC24192<br>MAGVSAVSKVSSLVCDLSSTSGLIRRTANPHPNVWGYDLVHSLKSPYIDSSYRERAEVLVSEIKAMLNPAITGDGESMITPSAYDTAWV<br>ARVPAIDGSARPQFPQTVDWILKNQLKDGSWGIQSHFLLSDRLLATLSCVLVLLKWNVGDLQVEQGIEFIKSNLELVKDETDQDSLVT<br>DFEIIFPSLLREAQSLRLGLPYDLPYIHLLQTKRQERLAKLSREEIYAVPSPLLYSLEGIQDIVEWERIMEVQSQDGSFLSSPASTACVFMH<br>TGDAKCLEFLNSVMIKFGNFVPCLYPVDLLERLLIVDNIVRLGIYRHFEKEIKEALDYVYRHWNERGIGWGRLNPIADLETTALGFRLL<br>RLHRYNVSPAIFDNFKDANGKFICSTGQFNKDVASMLNLYRASQLAFPGENILDEAKSFATKYLREALEKSETSSAWNNKQNLSQEIK<br>YALKTSWHASVPRVEAKRYCQVYRPDYARIAKCVYKLPYVNNEKFLELGKLDFNIIQSIHQEEMKNVTSWFRDSGLPLFTFARERPLE<br>FYFLVAAGTYEPQYAKCRFLFTKVACLQTVLDDMYDTYGTLDELKLFTEAVRRWDLSFTENLPDYMKLCYQIYYDIVHEVAWEAEKE<br>QGRELVSFFRKGWEDYLLGYYEEAEWLAAEYVPTLDEYIKNGITSIGQRILLLSGVLIMDGQLLSQEALEKVDYPGRRVLTELNSLISRL<br>ADDTKTYKAEKARGELASSIECYMKDHPECTEEEALDHIYSILEPAVKELTREFLKPDDVPFACKKMLFEETRVTMVIFKDGDGFGVSK<br>LEVKDHIKECLIEPLPL<br>>AAB05407<br>MAMPSSSLSSQIPTAAHHLTANAQSIPHFSTTLNAGSSASKRRSLYLRWGKGSNKIIACVGEGGATSVPYQSAEKNDSLSSSTLVKREFP<br>PGFWKDDLIDSLTSSHKVAASDEKRIETLISEIKNMFRCMGYGETNPSAYDTAWVARIPAVDGSDNPHFPETVEWILQNQLKDGSWG<br>EGFYFLAYDRILATLACIITLTLWRTGETQVQKGIEFFRTQAGKMEDEADSHRPSGFEIVFPAMLKEAKILGLDLPYDLPFLKQIIEKREA<br>KLKRIPTDVLYALPTTLLYSLEGLQEIVDWQKIMKLQSKDGSFLSSPASTAAVFMRTGNKKCLDFLNFVLKKFGNHVPCHYPLDLFER<br>LWAVDTVERLGIDRHFKEEIKEALDYVYSHWDERGIGWARENPVPDIDDTAMGLRILRLHGYNVSSDVLKTFRDENGEFFCFLGQTQ<br>RGVTDMLNVNRCSHVSFPGETIMEEAKLCTERYLRNALENVDAFDKWAFKKNIRGEVEYALKYPWHKSMPRLEARSYIENYGPDDV<br>WLGKTVYMMPYISNEKYLELAKLDFNKVQSIHQTELQDLRRWWKSSGFTDLNFTRERVTEIYFSPASFIFEPEFSKCREVYTKTSNFTVI<br>LDDLYDAHGSLDDLKLFTESVKRWDLSLVDQMPQQMKICFVGFYNTFNDIAKEGRERQGRDVLGYIQNVWKVQLEAYTKEAEWSE<br>AKYVPSFNEYIENASVSIALGTVVLISALFTGEVLTDEVLSKIDRESRFLQLMGLTGRLVNDTKTYQAERGQGEVASAIQCYMKDHPKIS<br>EEEALQHVYSVMENALEELNREFVNNKIPDIYKRLVFETARIMQLFYMQGDGLTLSHDMEIKEHVKNCLFQPVA
Apostasia shenzhenica	>Ash014324

SNMSEDINLHCSALLFRLLREHGTDSSNLLGSNPIFSSLNAMIVSFKGRIQHDVKELLSLHEASFLAVEGEDELEEAGEMAAEQLRRLR
KNTSLDPKLAEHIDHALELPLHWRMTRLHTRWYIDFYEKQENADSGLLELAKLDFNMVQSAYKMELKEISRWWRNLGLASEELSFA
RDRLVEAYFWSVGCSYEPHFWRCRKEIAKIGCLLSTIDDIYDVYGSLEELELFTRAVDCLDLSKAYLLEARWYYSGYTPTLNEYIENGW
MTIAGPLLSACAYCLSEDLTMEALNNLRFYPDVVRQSSLIARLYDDLGTSNDEILRGDVAKSIQCHMKHKNVSEKAAREHIRWLIRK
HWKTMNEGLIANQQLLGSFKSHVINFPRMAQFIY
>Ash013010
DEVNGDVKSSALLFRLLRQQGFFVSQDMLVNSFKDEEGRYKAELSHDMKGLLSLYEASFLAWEEEEEELEKAKIFAAEHLIRALGHGES
ELTEEIEYSMELPLHWRAPRLHALRFIHTCEKRGGIDPSLLELAKLDFNMVQSIYKDEVKDVSRWWRNLGLSGGKLGFARDWPVESYI
WTVGLVYEPQFSRCRRELAKMVCFLHLVDDIYDVYGSPHELELFTTAVERWEVGALDELPEYMKICYLALFNTTNDIAYSILKDKGVD
TLPYLKKAWRDICNAFLAEARWYNSGYIPKFNEYMDNAWISGAAPLSLIFAQCLSQDLTHKSLQHLQPFYPSATRYSSTIFRLYDDIGT
SKVEIERGDVAKSIQCYVNEKNVVEERAREKIKDEIHKYWKLLNQERANFSEFEETFKRAVMNLPRMAHFMY
>Ash000699
SLIDADDLYLVFLQFRLLRQHGYNITSDVFDKFRDDERKFKGDLVNDVKGLLSLYEACYLATHEDGLIMDEALSFTKHHLQALSMEG
SLDGALKALVLHALETPLHRRTQRVEARHYIGLYEHDKEQRNDLLLELAKLDFHLLQLLHYEEAKILTLWWEALGLAEKFSTFTRNRI
IECYFWILCVYSEPHYSRARIMAAKVIALLSISDDFYDVYGTLEELQGLTDVIQRWDVEAANRLNDYMRITLNVWNDTFNEFEEELSSD
QKSYRVNYIKEMLKVISRAWLQETKWRDEEYIPPLKEHLEVSGVTTCYNIVSCASYLGMDDVATKEVFDWMLTFPKYVHHACMICRI
VDDIRSHEFEQKRNHFASTVQSYMKEHEVSVDQACNALFKIVDDEWKSLNQEFLNSISKYPRAILNKVVNYVRFLDTIY
>Ash001839
ADTDHEHDLHFVALHFRLLRQQRYHVSSDVFAKFLDENGDFVESLKGDIKGMLSLYEAAHFGMPGEEILDKAIGFTEFHLNNNLPLI
ELDPELAVLVSSALDFPLAKRIDRPKAKSYLSIYENDEITCNKLLLEFAKMDFALLQAMHQDEARSISMWWKDSGYAEKLPFARDRV
MECYFWVLSVYHEPCYSRARLMMTKIISQISVVDDIYDLYGTSEELQIFTDAFERWELESAAQLPEYMKLSFLTIQKTFKDFEAELAPEN
NSFRKEYLKNELKQIVRSYLQESKWARERYIPKLEDHLKVTLVTAGYSFLTCASYMGMHGNIPREIFDWITSLPDVIKSSCIIGRLMNDI
VTYELEQKRNHVASTIQCYVMEHGCLEEEACKKLMEMVDDSWKIINREYVVCKNIPLSLMVPAVNLARFNYFVY
>Ash001894
TRVDELHDLHTVALRFRLLRQQRYDVSSDVFAKFLDENGEFMESLSNDIKSMLSLYEAAHFGMPEEEILEKAISFTEFHLLNINNLPSN
LEPHLAMMVSSAMDFPLAKRIDRPKTRSYLSIYENDHEGYYYCNQFLLHFAKVDFALLQAMHQDEARNISMWWKDAGTAKVFPFS

	RDRIIECYFWVLSVYYEPCYSRARLMMTKIISQMSILDDIYDVYGTLEELQIFTDAIQRWDLKSIAQLPDYMQYSFRIIQKIFRDFEAELAP<br>ENNSFRLEYLKNELKRVVQSYLQEAKWASQCHVPKLEDHLRVSLVTAGYSFLTCASYIGMHERITRDVFDWITSLPNIIKASCIIGRIM<br>NDVVTYKLEQKRNHVASTVQCYIMEHGCSNEEACKKLMEMAGDAWKIINREFVMHDKSLPLFTMMPAVNLARFNYLVY<br>>Ash010138<br>NDEEIYSDISTCAMAFRLLRMHGYDLSSDAFSQFSDATQFKNTVQGHFKDYDAAIELCKVSQTQVLAEEPVLEKLNSWVTRFLKEELN<br>SNAILSLDTAQEADYVLRFPFYANLERLEHKRNIEHYNFGSLQMLKTSFLSSIDRRILELAVDEFSNSQLIYRKELQYLESWVKEIKLDQL<br>KFSRQKQTYCYLSAAASLFSADLSDARICWAKGGVLTTVVDDFFDGGGSAEEQSNLIMLIEGWHENHQKDFCSESVEIIFFALYNTVN<br>DLSMKAFFPQKRDVTSHIVNIWLHMMKSMKVEAEWLKNNTVPSIDEYMAHAIPSFALGPIVLPSLYFVGPVVSEDAIESPEYFNLFKL<br>VSKLGRLLNDYQGFERDLKDGKMNSVSLRIQHGNGSISKEDARRETMRTIESTRAELLGLVLQEKGSKVPKSCKELFWKMSKILHLFY<br>>Ash010892<br>TDMEESIDLHGIALLFRLFRVYGDLQGSQLSLKSLVRSIKSESMIFKANIEQDIKGLLSVYEASHLAFEGDDELDEAGEFAAEKLRELRK<br>NSSLDPKLGEQIDYALEIPLHWRVPRFHARWFIDFHSNQKKIDSNLLELAKLDYNRIQSLYKNELKELSRWWKDLGLVCEKLGFARN<br>RPVENYLWALGSAYEPQFWRIRKENFKLNCVVVVIDDIYDVFGTLDELELFTNAIERWIIAPELPEYMKICLLAFFNTVDDIANKILLEK<br>GLHIQLYLRQSCVDVCKAYFKEARWYHGGHNPNLHEYLENAWISIAAIIVLTEAYCLSDDLTAEALQNFKFYPDVVLHSSILIRLYND<br>LGTLSAEMQRGDVSKSVQCYMNDKNVSESDARKYIKRILIRKHWKALNETLAANPNLLGSFKTGLLGLPQMSQFLY<br>>Ash010893<br>SDMEESVNLHGIALLFRLFREYGDLQNSQLSLESLIRSIKSESLKVNIEQDIKGLLSVYEASYLAFGGEDELDEAGEFAAKQLSELRKNSS<br>LDPKLGEQIDYALEIPLHWRVPRLHARWFIDFHSNQKKIDSNLLELAKLDYNRIQSLYKKELKELSRWWKDLNLVCEELGFARNRPM<br>ENYLWALGCAYEPQFWRSRKENFKIICLIAIIDDIYDIFGTLDELELLTNAIEQWIIAPELPEYMKICLLAFFNTVNDIANKILLEKGLHIQ<br>LYLRQSCVDICKAYLTEARWYHGGHTPKLHEYLENAWISIGANVVLTEAYCLSDDLTAEALENFKFYPDVLRHSSILIRLYDDLGTSSA<br>EMQRGDVPKSVQCYMNDKNVSESDARKYIKRFLIRKHWKALNERFAANPNLLGSFKTGLLGLPRISQFLY<br>>Ash021680<br>MHVNFFVDCFKRFMNKEGNFEVSLCYDTKGLMSLYEASYVDMGEEILCKAREFSTKHLKASERYPENIDAEEKIIKRSLSNPIYKSLPR<br>FEARHFIGGYKGNIGASKVLKDLAIMDFNTVQSLHKSELSKVVR
Arabidopsis thaliana	>AtTPS29<br>DIIVGEDDLETISIMFEVFKLYGHKMSCDAFDRFRGNDGRFKESLVRDFRGMLQLFEVAHLGTPCEVIMDEALSFTRNHLESLTSGNAS

TASPHLLKHIQNSLYIPRYCNIEVLVAREYISYYEQEEGHDEILLKFAKLNFNFCQFHYVQELKTLTKWWRDLDLASKLPYIRDRLVES
HLVALGPYFEPHYSLGRIIVAKINMIMVVVDDTYDAYATLPQVKALTECLQSIEVSDLPDYLRIVLGSLFDVMGEIEREMRPLGRLYRV
KQVVEKIKIITKAYQEIAKWARTGHVSTFDEYMKVGVLTAGMADYAAYCFIGMEDINEKEAFEWLNSNPLIIKHLTAMFRLANDVGT
YETEINRGEVANGLNCYMKQYGVTKEEASRELRKMYVYRKKVVVEEFMHVPRQVLLRCLNIARIFDVFY
>AtTPS22
MLFTDEDDLETTAIMFEVFRLYGHKISCDVFDRFKGVDAKFKEHLVSDVRGMLQLYEAAHLATPFETILDEALSFTRYHLESLAGQQA
TAPHISRHILNALYKPRFLKMEIIAAREYIHFYQKEGHDETLLKFAKLNFNFCQLHYVRELKTLTKWWKDIDLPYKLPYIRDRLLETFIG
VMAVYLEPHYSLGRIIATKVSQVIVVMDDTCDAYGTFSEVRSLIDSLERWDPGAIDKLPSCLRIVIQSIVETMEDIEREMKPRGRSSSVQD
TVEEIKIMGRAYAEISKWARAGHVPTFDDYIELGLDSSGIRCFAMYSFISMEDCEENQTNAWFKSKPKMLRALSVIFRLTNDIAGFEEE
MRRGEVVNGVNCYVKQHNVTKELAVREIKKMIRDNYKIMMEEFLSVSRPILVRCFNIVRLVNLYY
>AtTPS23
WKCDKEEDLHATSLEFRLLRQHGFDVSENIFDVIIDQIESNTFKTNNINGIISLYEASYLSTKSDTKLHKVIRPFATEQIRKFVDDEDTKNI
EVKAYHALEMPYHWRMRRLDTRWYIDAYEKKHDMNLVLIEFAKIDFNIVQAAHQEDLKYVSRWWKDTCLTNQLPFVRDRIVENYF
WTVGLIYEPQFGYIRRIMTIVNALVTTIDDIYDIYGTLEELELFTSMVENWDVNRLGELPEYMRLCFLILYNEINGIGCDILKYKKIDVIPY
LKKSWADLCRTYLVEAKWYKRGYKPSLEEYMQNAWISISAPTILIHFYCVFSDQISVQNLETLSQHRQHIVRCSATVLRLANDLGTSPT
ELARGDVLKSVQCYMHETGASEERARDHVHQMISDMWDDMNSETKSRSRGFKEAAMNLARMSQCMY
>AtTPS28
EMMDGENDLYTVSIIFWVFRTYGHNISSDIFNRFKGHNGKFKECLATDAKGILSLYEAAHMGTTTDYILDEALSFTLSYLESLAANGTC
KPNLVRRIRNALGLLQNKNVEILVAKEYIRFYEQEEDCDKTILEFSMLNLKFLQLHYLQELKLLTKWYKEQDFESKLPPYYRDRIVELH
LATLAYINPKYSRVRIILTMIYTIQIILDDTCDRYASLREVESLAATIERWDHHAMEGLPDYLKSVAKFIFHTFQEFEREVSSESGGSYSLK
ATIEDCKRMMRSNLQLAKWAVTGHLPSFDEYLDVAGVEIAVYFTVAGILLGMENINKKEAYEWLIFRDKLVRAMSTKARLVNDLFG
YKDDMRRGYVTNSINCYKKQYGVTEEEAFRKLHQMVADGDKMMNEEFLNVPHQVLKAVLDTLRAINICY
>AtTPS4
PMSYLADQLHKDSLAFRMLRMHGRDVSPRSFCWFLNQETRNHLERNIDSFLLVILSVYRATDLMFPGEHDLQEAREYTRNLLEKRRSI
KEMIMHELSTPWIARLKHLDHRMWIEDKNSNVLSMEKASFLRLHSSYSDKLTHLAARNFEFQQAKYCRELEELTMWVKKWGLSDIG
FGREKTTYCYFATVTSLPYEYAIKFGKLAAKTAILITIADDFFDEKGSFNDLEGLTKAVLRWEGELKSYGNIIFRALDDIVRETANTCRT

HHKTDIIVHLRNIWGETFESWLREAEWSKKGHTSSMDEYIRNGMISIAAHTIALSISCLMEPCFPHNKLKPGNYDSITTLLMIIPRLLND
LQSYQKEQEQGKMNSVLLHMKNHGLEIEDSIAHIEKIIDSKRKEFLEHVLDLPKPCKEIHMSCCKVFEMFF
>AtTPS14
QFNGDLHEIALRFRLLRQEGHYVQEIIKNILDKKGGFKDVVKNDVKGLTELFEASELRVEGEETLDGAREFTYSRLNELCSGRESHQKE
IMKSLAQPRHKTVRGLTSKRMIKIAGQEDPEWLQSLLRVAEIDSIRLKSLTQGEMSQTFKWWTELGLEKDVEKARSQPLKWHTWSMK
ILQDPTLTEQRLDLTKPISLVYVIDDIFDVYGELEELTIFTRVVERWDHKGLKTLPKYMRVCFEALDMITTEISMKIYKSHGWNPTYALR
QSWASLCKAFLVEAKWFNSGYLPTTEEYMKNGVVSSGVHLVMLHAYILLGEELTKEKVELIESNPGIVSSAATILRLWDDLGSAKDEN
QDGTDGSYVECYLNEYGSTVDEARTHVAQKISRAWKRLNRECLPFSRSFSKACLNIARTVPLMY
>AtTPS26
EMMEDEDDLCTVSIIFWAFRRYGHYISSDVFRRFKGSNGNFKESLTGYAKGMLSLYEAAHLGTTKDYILQEALSFTSSHLESLAACGTC
PPHLSVHIQNVLSVPQHWNMEILVPVEYIPFYEQEKDHDEILLKFAKLSFKLLQLQYIQDLKIVTKWYKELEFASKLPPYFRDNIVVNYF
YVLAVIYTPQHSYERIMLTQYFTCLAILDDTFDRYASLPEAISLANSLERWAPNDAMDQPDYLKIVLNFILKTFEVFQKELEPEGRSYTV
KATIEEFKTVTKGNFDLAKWAHAVHVPSFEEYMEVGEEEISVCSTLAGIFMCMEKATKEDYEWLKSRPKFIQTLCARCRLKNDITGFE
DDMSRGYVTNAVNCYMKQYGVTKQEAFGELNKIIVEADKILNEEFLGVRHCVLKATFDLARMIFITY
>AtTPS6
IIDCKEDDLYTVSIIFRVFRLYGHYITPDIFNRFKGDDGNFKKCLNDDVRGMLSFYEASHFGTTTEDILEEAMSFTQKHLELFLVGEKAK
HYPHITKLIQAALYIPQNFNLEILVAREYIDFYELETDHNEMLLKLAKLNFRFLQLQYIQDLKTLTTWWKELDLVSKIPVYFRERLAEPY
FWATGIYYEPQYSAARIMLAKSIILVDIVDNTFDVYGTIDEVKSLVQAIERWDSDAVDVLPDYLKVVFRTTFDLFKELEEYVSSEARSFT
MQYAYEQLRILMKGYLQEAEWSNRGHLPSHEEYIEVGVASTAGEVLLAMTFIPMGDAAGVGVYEWLRSRPKLTHALFVKSRLRDDI
ATYKEEMKRGDVCNGINCYTKQHKVSEEEACIEFEKKTNHMSKVMNEEFLFIPLHILRPVLNYGRLADVCY
>AtTPS32
GDEEICLDLATCALAFRLLLAHGYDVSYDPLKPFAEESGFSDTLEGYVKNTFSVLELFKAAQSYPHESALKKQCCWTKQYLEMELSSW
VKTSVRDKYLKEVEDALAFPSYASLERSDHRRKILNGSAVENTRVTKTSYRLHNICTSDILKLAVDDFNFCQSIHREEMERLDRWIVEN
RLQELKFARQKLAYCYFSGAATLFSPELSDARISWAKGGVLTTVVDDFFDVGGSKEELENLIHLVEKWDLNGVPESSEHVEIIFSVLRD
TILETGDKAFTYQGRNVTHHIVKIWLDLLKSMLREAEWSSDKSTPSLEDYMENAYISFALGPIVLPATYLIGPPLPEKTVDSHQYNQLY
KLVSTMGRLLNDIQGFKRESAEGKLNAVSLHMERNRSKEVIIESMKGLAERKREELHKLVLVVPRECKEAFLKMSKVLNLFY

>AtTPS5
TTMAGENDLSTVSVMFWVSGHMDINMFRRFKGEDGKFEECHTKDVKGLLSLYEAAQLGTSTEDILDEAMSFSSSHLECLAGGTCPP
HISRLIQNELYMPQHHNAEILFASEYISFYKQEDVHNKVLLEFAKLNFKFLQLHWIHELKILTKWWNDQDLLSKLPPYFRDRMVECH
LYAVIYYFEPQYSFGRIMLAKLLVLLTVVDDTCDRYGSVPEVAKLLDCVERWDPELGESLPDYLKTVFKFTLDVFEDCERAGKSEEGLS
FNVDGALAERTHLNFAEWAAAEKVPTVEEYLEVGGVAVTMYATIALGLLGLGKAREHGYEWLKSRPKLVHDLATKGRLMNDMG
GFKDDIGRGFLANVVNYYMKEYGTTEEETYKEFHKIVRDLEKSVNSEFLGVPREILSRALNCGKMIDVTY
>AtTPS10
TNIQESDLHATALEFRLFRQHGFNVSEDVFDVFMENCGKFDRDDIYGLISLYEASYLSTKLDKNLQIFIRPFATQQLRDFVDTHSNEDF
GSCDMVEIVVQALDMPYYWQMRRLSTRWYIDVYGKRQNYKNLVVVEFAKIDFNIVQAIHQEELKNVSSWWMETGLGKQLYFARD
RIVENYFWTIGQIQEPQYGYVRQTMTKINALLTTIDDIYDIYGTLEELQLFTVAFENWDINRLDELPEYMRLCFLVIYNEVNSIACEILRT
KNINVIPFLKKSWTDVSKAYLVEAKWYKSGHKPNLEEYMQNARISISSPTIFVHFYCVFSDQLSIQVLETLSQHQQNVVRCSSSVFRLA
NDLVTSPDELARGDVCKSIQCYMSETGASEDKARSHVRQMINDLWDEMNYEKMILHHDFMETVINLARMSQCMY
>AtTPS17
DIIAKENDLETISTMFEVFRLRGYYMPCYAFNRFKGEDGRFKESLAEDIRGMLQLYEAAHLGTPSEDIMDEALSFTRYRLESLTSNHTA
TASPHLSKHIQNALYRARYHNLEILVAREYISFYEQEEDHDETLLKFAKLNFNYCQLHYIQELKDLTKWWKELDLASKLPYIRDRIVEV
YFGALALYFEPRYSLGRIIVTKITMIVTVFNDTCDAYGTLPEVTSLVDSFQRWDLGDIEKLPSYVKIVFRGVFETLEEIEQEMRPQGRSRIV
QVAVDEIKKLGKAYLAISKWARASHVPTFEEYMEFGMQTSMDHFAAYSFIAMEDCDENQTCEWYKSRPKMMEALNGVFRIKNDIN
TFEQEMSRGEVAKGLNCYMKQHGVSKEEAIGEMNKIYSNYYKIIMEEYLAVPRPILVRCLNVSRPIHHFY
>AtTPS18
DIIVGEDDLETISIMFEVFRLYGHKMSCDAFDRFRGEDGRFKESLAKDVRGMLQLFEVAHLGTPSEDIMDEASSFAQNHLDSWIGGNV
SGATPHLLKHIQNSLYIPRYCNIEVLVAREYISYYEQEEGHNKILLKFAKLNFNFCQFHYIQELKTLTKWWKDLDLASKLPYIRDRLVES
HLGGLGPYFEPHYSLGRIIVAKIIMTMVVVDDTYDAHATVPEVAVLTECLQRLNIGADDKLPDYLRTVLESVFEVMGEIEQEMRPKGR
SYGVKQVLERFKNVAKADKQLTEWARTGDVPSFDEYMKVGLVTAGMDGYAGYCFIGMEDVSEKEAFEWLSSNPLIIQALNVMFRL
ANDVGTYETEINRGEVANGLNCYMKQYGVTKEEASQELRKIYSNNKKVVMEEFMHVPRQVLLRCLNFARLFDVMY
>AtTPS19
GIIVEEDDLETISIMFEVFRLYGHKMSCDAFDRFRGGDGRFKESLAKDVRGMLQLFEVAHLGTLSEDIMDEALRFTRNHLESLTSGNVS

SASPHILKHIQNSLYIPRYCNIEVLVAREYISYYEQEEGYNEILLKFAKLNFNFCQCHYIQEIKTLTKWWKDLDLASKLPYIRDRSVESHL
GGLGPYFEPQYSLGRIIVAKTIMIIVVADDTYDAHATIPEATVLTEYFQRLNIGADDKLSGYLRIVLESVFEVMGEIEQEMSPKGRSYSVK
QVLERFKIIAKAYKQLTEWARKGHVPTFDEYMKVGLVTAGMGDYAGYCFIGMEDINEKEAFEWLNSNPLLIDALNVLFRIANDVGT
YETEINRGEVANGLNCYMKQYGVTKEEASRELRKMYIYNKKVVVEEFMRVPRQVLLRCLNFARLFDVIY
>AtTPS24
WKCDKEEDLHATALEFRLLRQHGFGVSEDIFDVIIDKIESNTFKSDNITSIITLYEASYLSTKSDTKLHKVIRPFATEQIRNFVDDESETYN
IMLRMAIHALEIPYHWRMRRLETRWYIDAYEKKHDMNLFLAEFAKIDFNIVQTAHQEDVKYVSCWWKETGLGSQLHFVRDRIVENY
FWTVGMIYEPQFGYIRRIVAIVAALITVIDDIYDIYGTPEELELFTAMVQNWDINRLDELPEYMKLCFLTLFNEINAMGCDVLKCKNID
VIPYFKKSWADLCKAYLVEAKWYKGGYKPSVEEYMQNAWISISAPTMLIHFYCAFSGQISVQILESLVQQQQDVVRCSATVLRLAND
LATSPDELARGDVLKSVQCYMHETGVSEEEARTHVQQMISHTWDEMNYETALLSRRFVETAMNLARMSQCMY
>AtTPS27
WKCDKEEDLHATSLEFRLLRQHGFDVSENIFDVIIDQIESNTFKTNNINGIISLYEASYLSTKSDTKLHKVIRPFATEQIRKFVDDEDTKNI
EVKAYHALEMPYHWRMRRLDTRWYIDAYEKKHDMNLVLIEFAKIDFNIVQAAHQEDLKYVSRWWKDTCLTNQLPFVRDRIVENYF
WTVGLIYEPQFGYIRRIMTIVNALVTTIDDIYDIYGTLEELELFTSMVENWDVNRLGELPEYMRLCFLILYNEINGIGCDILKYKKIDVIPY
LKKSWADLCRTYLVEAKWYKRGYKPSLEEYMQNAWISISAPTILIHFYCVFSDQISVQNLETLSQHRQHIVRCSATVLRLANDLGTSPT
ELARGDVLKSVQCYMHETGASEERARDHVHQMISDMWDDMNSETKSRSRGFKEAAMNLARMSQCMY
>AtTPS16
EIIAGEDDLYTISTIFWVFRTYGYNMSSDVFRRFKEENGKFKESLIEDARGMLSLYEAAHLGTTTDYILDEALDFASNNLVSLAEDGMC
PSHLSTHIRNALSISQHWNMEIIVAVQYIRFYEQEVGHDEMLLKFAKLNFNLVQRLYLQEVKILTKWYKDQDIHSKLPPYYRPVVTEM
HFFSTATFFEPQFSHARILQTKLFMAELLVDDTCDRYATFSEVESLINSLQRWAPDAMDTHPDYLKVVFKFILNAFEECEKELRPQGRS
YSLEQTKEEYKRFAKSNLDLAKLAQAGNVPSFEEYMEVGKDEIGAFVIVAGSLMGMDNIDAVEAYDFLKSRSKFSQSSAEIVRYLNDL
AGFEDDMRRGCVSTGLNCYMNQYGVTETEVFREFRKMVMNTCKIMNEEFLDVPLRVLKTNFSCVRSGFVGY
>AtTPS15
EMMANEEDLYTVSIIFWVFRRYGHYISSDFFRRFKGNDGNFKKSLIGDAKGMLSFYEAANMATTKDYILDEALSFTSSHLESLAANGA
CPPHMSRRIRNALNASQHWNMEMLVAVEYISFYEKEKDHNEMLLKFSKLNFKFLQLQYLQELKVLTKWYKEVDFVSKLPPYFRDRIV
ENHFFIQTLFVESQHSRARIMMAKYFILLVIQDDTLDRYASLPEAESLVNSLNRWAPDHAMDQPDYLKFVFKFILDTFEEFEKELRPEG

GSFGVCATIEEFKSLVKANLEAEKWALADNMPSFEEYIEVTGVGITAMTTLMGAMMCMGKIVPKEDYKWLKSRPKIIQALAIKGRLM
NDMKGYKEDMSRGYAANAVTCYMKQYRVTEQEALKEFEKMVAVANKTVNEEFLGVSRLVLKLAMGVGLMISITY
>AtTPS25
CLISDEDDLETIAIMFEVFRLYGHKMPCDVFERFKSEDGKFKESLVGDVRGLLQLYEAAHLGAPSEDIMDEALSFARYHLEPLAGTETS
SNLFKHVENVLYRARYHSIEILVARQYISFYDQEEDQDETLLRFSKLNFNFCQMHYVKELKIVTRWWKELGIASKLPYSIRERNVETYL
GGLGVLFEPRYSLARIFLAKLTLIMTVVDDTCDAYATLPEVQSLHDAFHRWDLRAMEELPRYMRIIYQSVFETVEDIDREMIARGKHG
RLQLTIDEIKSLMIWYLGIAKWARSDQVPSFEDYMEIGTPSSALDDFASYGFIAMDDCDQKQLKEWFYSKPKIFHALNALFRIRNDIVT
FEQEMSRGEVANGVNCYMKQHGVTKEAAVEELRKMERESYKIMIEEFMAMPRQILVRPVNIARVMDLFY
>AtTPS30
MIIAEEDDLETISIMFEVFRLYQHKMSCDSFVRFKGEDGRLKESLVGDVRGMLQLYQAAHLGTPSDQYIMEEAKSFTRNHLESLVESTT
IPPHFSSHIRDALYIDRYHNMEILVARKYISFYEQEEGHDLTLLKFGKLSFNYCRLHYIQELKTLTKWWKDQDIPSNLPCVRDRIVETYF
PTLGLYFEPRFSLGRIIIAKMTIIVVALNDVCDSYATYPEAKSLIDSLQRWDIEAIDELPNYSRIVLRLILETIGEIEREMKPRGRSASVQHTI
DETKSLGRAYLALSKWASEGYMPTFDEYMEVGEVTGGMDDFALYSFIAMEDCDEKPLYEWFDSKPKILQALSVLYRINNDIVTYERE
MSKGEVVNGVNSYMNQHGVTKEEAVEELRKMARDNYKIVMEELLDVPRPVLVRCLNLARLFDVFC
>AtTPS31
ARCSHVQDIDDTAMAFRLLRQHGYQVSADVFKNFEKEGEFFCFVGQSNQAVTGMFNLYRASQLAFPREEILKNAKEFSYNYLLEKRE
RELIDKWIIMKDLPGEIGFALEIPWYASLPRVETRFYIDQYGGENDVWIGKTLYRMPYVNNNGYLELAKQDYNNCQAQHQLEWDIF
QKWYEENRLSEWGVRRSELLECYYLAAATIFESERSHERMVWAKSSVLVKAISSSFSSDSSFDQFHEYIANARRSDHNDRNDRPVQAS
RLAGVLIGTLNQMSFDLFMSHGRDVNNLLYLSWGDWMEKWKLYGDEGEGELMVKMIILMKNNDLTNFFTHTHFVRLAEIINRICLP
RQYKARRNDEKEKTIKSMEKEMGKMVELALSESDTFRDVFLDVAKAFYYF
>AtTPS12
DITGDEKDLSTISIMFRVFRTYGHNLPSSVFKRFTGDDGKFQQSLTEDAKGILSLYEAAHLGTTTDYILDEALKFTSSHLKSLLAGGTCRP
HILRLIRNTLYLPQRWNMEAVIAREYISFYEQEEDHDKMLLRLAKLNFKLLQLHYIKELKSFIKWWMELGLTSKWPSQFRERIVEAWL
AGLMMYFEPQFSGGRVIAAKFNYLLTILDDACDHYFSIHELTRLVACVERWSPDGIDTLEDISRSVFKLMLDVFDDIGKGVRSEGSSYH
LKEMLEELNTLVRANLDLVKWARGIQTAGKEAYEWVRSRPRLIKSLAAKGRLMDDITDFDSDMSNGFAANAINYYMKQFVTKEEAI
LECQRMIVDINKTINEELLSVPGRVLKQALNFGRLLELLY

>AtTPS13
DITGDENDLSTISIMFRVFRTYGHNLPSSVFKRFTGDDGKFERSLTEDAKGILSLYEAAHLGTTTDYILDEALEFTSSHLKSLLVGGMCRP
HILRLIRNTLYLPQRWNMEAVIAREYISFYEQEEDHDKMLLRLAKLNFKLLQLHYIKELKTFIKWWMELGLTSKWPSQFRERIVEAWL
AGLMMYFEPQFSGGRVIAAKFNYLLTILDDACDHYFSIPELTRLVDCVERWNHDGIHTLEDISRIIFKLALDVFDDIGRGVRSKGCSYY
LKEMLEELKILVRANLDLVKWARGNQLPSFEEHVEVGGIALTTYATLMYSFVGMGEAVGKEAYEWVRSRPRLIKSLAAKGRLMDDIT
DFESDMSNGFAANAINYYMKQFVTKEEAILECQKMVVDINKIVNEELLTVPRRVLKQALNFGRLLEVLY
>AtTPS1
EMIAGEDDLYTISIMFWVFRTYGYNMSTDVFKRFKGENEKFMESITSDVKGMVSLYEAAHLRTTREDILEEALSFTTRNLESLARAGAS
SPHILMRIRNALCMPQHYNAEMIFAREYISFYEQEEDHNKMLLRFAKINFKFLQLNWIQELKTLTKWWKQQDLASKLPPYFRDRLIEC
YLFAIMIYFEPQFSLGRVSLAKINTVFTLVDDTCDRYGNVSEVAALVQCVERWDPDCMDSLPDYMKTVFKFAWNTFEECENAGIMEE
GLSYDVQGALEEWEQGDVVPTFDEYLEIGGVEVTMYVSIACSFLGLGSSREQAYKWLKSRPKFVEAQAKRARLMNDIAGFEGDMSR
GFDVNAIMYYMKQYKVTEEETFTRLQKMARDLDTTVNEEILSVPRQILKRAIDFGKMIEFTY
>AtTPS2
EIERETQDLHATSLEFILLRQHGFDVSQDAFDVFISETGEFRKTLHSDIKGLLSLYEASYFSMDSEFKLKETRIYANKRLSEFVAESKTICR
EDETYILEMVKRALETPYHWSIRRLEARWYINVYEKKHEMNPLLLEFAAIDFNMLQANHQEELKLISSWWNSTGLMKQLDFVRDRIT
ESYFWTIGIFYEPEFKYCRKILTKIFMLIVIMDDIYDIYGTLEELELFTNVVEKWDVNHVERLPNYMRMCFLFLYNEINQIGYDVLRDKG
LNVIPYLKQVWTDLFKTFLTESKWYKTGHKPSFEEYMQNGVISSSVPTILLHLFSVLSDHISDQTLTDDSKNHSVVRSCATILRLANDLA
TSTEEMARGDSPKSVQCYMYETRASEEEARRHMQSMISDSWDIINSDLKSLPRGFLAAAANLNRVVQCIY
>AtTPS3
IDRNRWGDLYATALEFRLLRQHGFSIAQDVFDGNIGVDLDDKDIKGILSLYEASYLSTRIDTKLKESIYYTTKRLRKFVEVNKNETKSYT
LRRMVIHALEMPYHRRVGRLEARWYIEVYGERHDMNPILLELAKLDFNFVQAIHQDELKSLSSWWSKTGLTKHLDFVRDRITEGYFS
SVGVMYEPEFAYHRQMLTKVFMLITTIDDIYDIYGTLEELQLFTTIVEKWDVNRLEELPNYMKLCFLCLVNEINQIGYFVLRDKGFNVI
PYLKESWADMCTTFLKEAKWYKSGYKPNFEEYMQNGWISSSVPTILLHLFCLLSDQTLDILGSYNHSVVRSSATILRLANDLATSSEEL
ARGDTMKSVQCHMHETGASEAESRAYIQGIIGVAWDDLNMESCRLHQGFLEAAANLGRVAQCVY
>AtTPS7
KMMDGEDDLYTVSIIFWVFRRHGYHISYGVFQRFKGSNGNFKESLTRDAKGMLSLYEAANLGTTKDFILEEALSFTSSHLESLAASGTC

PPHLSVRIRNALGLSQHWNMEMLVPVEFIPFYEQEIEHDEMLLKFAKLSFKLGQLQYLQELKTLTKWYKELDFATNLPPYFRDRIVEH
HFLVQAVFFSPQLSRERIMMIQYFTGLALLDDTFDRYASLHEAESLANSLERWAPDQAMDQPDYLRFVLNFILDTFEEFKRELGPEERS
YSVNATIEEFKAAAKANIDLEKWAQADHIPSFEEYMEVGEVEVTVYASLAGIFMSMGMATKEAFEWLKSRPKLVQYLSIKGRLMNDL
MGYEDDMSRGYVTNAVNCYMKQYGVTKEEAFRELYKIVVAANKTLNEEFLGVPHFLLKATIDLARMMTVAY
>AtTPS8
EMMAGEDNLYTISIIFLVLRTYGHHMSSDIFQKFKGNDGNFKGCISGDAKGLLALYEAAQLRTTTEYIMEEALSFTSSNLELLAADGRC
PPHLSKHIRNALGLSQHKQMEVLVAVEYISFYEQEKDHDKILLKFAKLNFKLMQLHYLEELKVVTKWYKEHDFASNLPPYFKYVIVE
NHFFAITMYFEPKFSQKRIMLAKYFTVLVLLDDTCDRYASLSEAESLTNSLERWAPDDAMDQPHYLKFVFKFIMGCFEEFERELASEGR
SYSVKATLEEFKTIVKANFDFAKLAHTGHVPSFKEYMEVGEVEVGVCATLAGNLMCIGHIGDEGVYEWLKSRPKFLKAASTYGRLM
NDIAGFEDDMKREYVITGVNTYMKQYGLTKMEAIRELQNLVEYNHTIMNEEFLDLPRQIRKQVINVARSLNVSY
>AtTPS9
KIMAGEEDLYTVSIIFWVFRRYGHYISSDVFQRFKGSNGSFKESLIGDAKGMLSLYEAAHLATTKDYILDEALIFTSSHLETLVATGTCPP
HLLARIRNALSICQHWNFEVLVPLDFIPFYEQEKDHDEMLLKFAKLSFKYLKLIYLQDLKILTKWYKKLDFPSKFPPYFKDRCVENYFF
VLPVFFEPQLSSARMLLTKGFILLGIQDDTFDRYASISEAESLGNSLKRWAPDHSMDQPEYLKSVLKVILDTFQEFEKELSPEGRSYSVKY
TIEEFQASSKANVELAKWAQVSHVPSFEKYMEVGQMEITACVTVAYILMSMGKTGTKEAFEWLKSRPKLVQSLCTKGRLMNDIAGFE
DDMSRGYVVNAVNCYMKQYGVTEKEAFKELRKMVVNTHKTLNEEFLCVSHYVLRETMDFARMIIVTY
>AtTPS21
MVRQEGCDLYTVGIIFQVFRQFGFKLSADVFEKFKDENGKFKGHLVTDAYGMLSLYEAAQWGTHGEDIIDEALAFSRSHLEEISSRSSP
HLAIRIKNALKHPYHKGISRIETRQYISYYEEEESCDPTLLEFAKIDFNLLQILHREELACVTRWHHEMEFKSKVTYTRHRITEAYLWSL
GTYFEPQYSQARVITTMALILFTALDDMYDAYGTMEELELFTDAMDEWLPVVPDEIPDSMKFIYNVTVEFYDKLDEELEKEGRSGCGF
HLKKSLQKTANGYMQEAKWLKKDYIATFDEYKENAILSSGYYALIAMTFVRMTDVAKLDAFEWLSSHPKIRVASEIISRFTDDISSYEF
EHKREHVATGIDCYMQQFGVSKERAVEVMGNIVSDAWKDLNQELMVFPFPLLMRVLNLSRVIDVFY
>AtTPS11
DLIADENKLHTISTIFRVFRTYGYYMSSDVFKIFKGDDGKFKESLIEDVKGMLSFYEAVHFGTTTDHILDEALSFTLNHLESLATGRRASP
PHISKLIQNALHIPQHRNIQALVAREYISFYEHEEDHDETLLKLAKLNFKFLQLHYFQELKTITMWWTKLDHTSNLPPNFRERTVETW
FAALMMYFEPQFSLGRIMSAKLYLVITFLDDACDTYGSISEVESLADCLERWDPDYMENLQGHMKTAFKFVMYLFKEYEEILRSQGRS

	FVLEKMIEEFKIIARKNLELVKWARGGHVPSFDEYIESGGAEIGTYATIACSIMGLGEIGKKEAFEWLISRPKLVRILGAKTRLMDDIADF<br>EEDMEKGYTANALNYYMNEHGVTKEEASRELEKMNGDMNKIVNEECLTMPRRILMQSVNYARSLDVLY<br>>AtTPS20<br>DMNLGEEDMYSISVIFRVFRLYRHKLSSDVFNRFKEENGDFKKCLLDDKKSLTKQWASRGNTWNYFVGGSNEEHLSGHIKNVLYLSQ<br>QENAEVVMSREYIQFYEQETHHDETLLKFAKINFKFMQLHYVQELQTIVKWWKELDLESKIPNYYRVRAVECLYWAMAVYMEPQYS<br>VARIILSKSLVLWTIIDDLYDAYCTLPEAIAFTENMERWETDAIDMPDHMKVLLRSLIDLMEDFKGEVRSEGRLYSVEYGIDEWKRLFR<br>ADLTISKWARTGYIPNYDEYMEVGIVTGGVDVTVAFAFIGMGEAGKEAFDWIRSRPKFIQTIDLKSRLRDDVATYKDEMARGEIATGI<br>NCYMKQYKVTEEEAFLEFHRRIKHTSKLVNEEYFTVPLKLVRIAFNVGRVIDTNY
Dendrobium officinale	>DoTPS34<br>NNSNEENNLNGSALLFRLLREHGINNASMLRVNALISCFKKEREKLNQNDEHNVKGMLSLYEASYLAVEGEEELEEAGKLAMEHLK<br>CIDGSLFTQQFIEEIDHALELPLHWRMSRLHTRWFIDAYGRRENFNPTLLELAKLDFNMVQSIYKAELQELSMWWRNLGLVCEELDF<br>VRDRLVENYLISLGITFQPNFARCRKAITKIISLVTTIDDIYDIYGTLDELKLFTNTIEEWKIDTSQQLPDYMKMCWIAIFNTMNDIATSFS<br>MEKALDILPCLKRVWVDLCKSQLLEAKWYHNGYTPTLDEYLENAWVSTSGHCELTAAYCLSDDLTIESLNSLEFYPPIIRYSCILFRLY<br>DDLQTSKEEMKRGDVPKSIQCYMKEKNVSESIARDYIKCLITIYWKKLNQECTTISKLWEPFRNALVGFPRTAQCFY<br>>DoTPS19<br>SNIIKNNLHYSSLLFRLLREYNIKVPNQSEDFFVRSFKNESGRFNINIMNDVKGMLSLYEASYLAVQGEDDLDDAVEFTTKHLSNYLKE<br>PLLINPLLVEEISHALELPLHWRMSRLHTMWFIDAYERQENMNPYLLEFAKLDFNIVQTIYKKELKEISRWWRSIGLVGDDFSFARDRL<br>METYLWSVGCAFEPRFWRCRKEITKLGCLITTIDDIYDVYGSLEELVFFTNVVDEWKIAESQHVSTCMKTVLLALFNTVNDIARTISTEK<br>GIDILPHLKRLWGDLCKSYLMEAIWYYIGYIPTINEYMQNAWLSISAPLILTSAYCLSEDLTIEALNSLELYIDATLHSSMITRLYDDLGTS<br>TVRTIMLLYNIYIYI<br>>DoTPS28<br>NNLIENNNLHDFALLFRLVREHDIHNASILRLDDLISCFKNEEESFNQNNQLDVKGMLNLYEASFLAMEGEDELDEAGKFAMKHLK<br>YHDRSLLSPQLVEQIEHALELPLHWRMSRLHTRWFIDAYGRHKNFNPTLLEFAKLDFNMVQSIYKTELQELSRWWRNINVVRGGLN<br>FVRDRLVENYLWAIGFTFQPELWRIRKAMTQIISFITTIDDIYDIYGTLDELKLFTNAIEAWNIATTQQLPGYMKKCLTTLFNTMDDIAS<br>SFSKEKELDILPCLKRMWADLCKAYFIEAMWYHNGYTPTLDEYLDNAWVTISGICVLTATYCLSDDLTFEAIKSLEFYPPIVRYSCMLL<br>RLYDDLGTCTEEIQRGDVPKSIQCYMKEKNVSETTARDYIRCLIRNYWKKLNKEHVISSKYVESFRKVLFDIPRTAQCFY

>DoTPS10
HNLIKNNNLYGSALLFRLLREHGINNAAILRVDTLISCFKKVRESFNPNHQYDVKEMLSLYEASYLAMEGEEELDEAGKFAMEHLRR
LDRSLLSPQLIEEIDHALELPLHWRMPRLHSRWFIDAYGKQENVNPILLELAKLDFNIVQSIYMTELKEISNWWRNLGVLCDELDFIRD
RLVENHLWALGFTFQPKFWRSRKAITKINCLVTTIDDVYDVYGTLDELEIFTNAVEDWKMDAAQQLPNCMKICLMALFNTMNEVA
YSFLKEKELDILPCLKRVWVDLCKAYLIEARWYHNRYTPTLDEYLENAWVTISGNCGLSASYCLSDDLNVEALDSIKFYPPIVQHSCMI
FRLYNDLGTDMAEIQRGDVLKSIQCYMNEKNVSDSAARDYIRCLIRNYWKKLNGEYITFSTSIESFRKALVDVPRTAQSFY
>DoTPS21
NNLIKNNNLYGSALLFRLLREHGINNGAILRVDTLMSCFKKVRESFNPNHQYDVKELLSLYEASYLAMEGEEELDEAGKFAMEHLRR
LDRSLLSPQLIEEIDHALELPLHWRMPRLHSRWFIDAYGKQENVNPILLDLAKLDFNIVQSIYMTELKEISNWWRNLGVLCDELDFIRD
RLVENHLWALGFTFQPKFWRSRKAITKINSLVTTIDDVYDVYGTLDELEIFTNAVEDWKMDAAQQLPNCMKICLMALFNTMNDIAY
SFLKEKGLDILPCLKRVWVDLCKAYLIEARWYHNRYTPTLDEYLENAWVTISGICELSASYCLSDDLNVEALDSINFYPPIVQHSCMIFR
LYNDLGTDMAEIQRGDVLKSIQCYMNEKNVSDSAARDNIRCLIRNYWKKLNGEYMTFSTSIESFRKALVDVPRTAQSFY
>DoTPS27
RMEFNGDVYAMALYFRLSRQHGFEISKDLLVKSYKYESGCFKPSVFDNIKGMLSLYEASFLAMDGEEEIDDAKEFALKHLNDFMRSN
SSTNPLLAQHIAMALELPLHHRIPKFQSQMFIEHSRHIKEINVDSIVLELGQLEFNMTQSIYKRELKEISKWWAEVSNVFDSKLNFARD
WPVESYFLAVGMAVEPQFSTYRKELAKALCFINVIDDIYDIYGLLDELQLFTNAVDRWEFASIKSLPEYMKICLEGLFNMVNSLASKIK
EEKGLDVLPNLKRAWLDLCKAYMVEARWCHTGYCPTFEEYLDNAWISVSTPLLSVISHCLSENLTKLSLESFDFYPTIVRQSSIIFRLYN
DLGTSKGELQRGDVSKSIQCYMKENHVSELVAQECIRNIINKNWKELNREWIKGSIYDETFKTVAINIPRAGHLMY
>DoTPS2
RMEFTEDVNFKALYFRLSRERGFEVSKDLLVKSYKDEKGCFKPCVFHDIKGLLSLYEASFLSMDGEDEIDEAKEFALKHLNDCMRSNS
LTNPALAKHIALALELPLHHRIQKLQAPMFIEHARNMKELDVDPILLEFAQLDFNMTQNIYNKELKEITRWWTKISNLAGGELSFARE
WPVESYFLAVGVAMEPRFSTCRKEFAKAICFINVVDDIYDIYGSLDELQLFTDAIERWDFSTSKSLPEYMKICLSELFNTMNSLACKIME
EKGLNILPYLKRAWLDLCKAYMVEVRWYYNGYCPTFAEYLDNAWISVAAPLGSVMTLCLSENLTNLSLEGFDFYPSIIRQSSIIFRLYN
DLGTSKGELRRGDVSKSLQCYMKEKHVSELEAQKCMKSIINKCWKELNREWIKASRYEETFKMVAINLPRVAHLFY
>DoTPS3
RMEFTEDVHVMALYFRLSREHGFEVSKDLLVKSYKDEKGCFKPCVFHDIKGLLSLYEASFLSMEGEDEIDEAKEFALKHLNDCMRSNS

STNLILAKHIALALELPLHHRIQKLQAPMFIEHARNMKELDVDPILLEFAQLDFNMTQSIYNRELKEITRWWTKISNLAGGELSFARE
WPLESYFLAICLAMEPRFSTCRKEFAKAVCFINVVDDIYDIYGSLDELQHFTDAVEMWDFATSKSLPKYMKICLSELFNTVNNLACKI
MEDKGLDILPYLKRAWLDLCKAYMVEATWYYIGYCPTFDEYLDNAWISVAAPLTSVMALCLSENLTNLSLESFDFYPSIIRQSSIIFRLC
NDLGTSKGELRRGDVSKSIQCYMKEKHVSESEAQKCIRTIIDKCWKDLNNEWMEASKYEETFKMVVINLPRVAHLFY
>DoTPS24
RMEFTEDVHVMALYFRLSREHGFEVSKDLLVKSYKDEKGFFKPYVFHDIKGLLSLYEASFLSMDGEDEIDEAKEFALKHLNDCMRSNS
TTNPLLAKHIALALELPLHHRIQKLQAPMFIEHARNMKELDVDPILFEFAQLDFNMTQSIYNRELKEITRWWAKISNLAGGELSFARE
WPLESYFLAVGLAIEPYFSTCRKEFAKAICFINVIDDIYDIYGSLDELQHFTDAVERWDFATSKSLPQYMKICLSELFNTVNSLACKIMK
DKGLDILPYLKRAVKLLKLLKIFKVLFL
>DoTPS20
SNILKNSLHGSALLFRLLREYGIKALNTREDFLVRSFKNENGSFKVHIVNDVKGMLSLYEASYLSVEGEDDLDEAMEFTTKHLSNYLKE
PSLIHPSLVEQISHALHLPLHWRMPTLHTMWFIDTYEKQENTNYSLFEFAKLDFNMVQSIYKKEVKEMSSWWRSIGLAGDEFSFARDR
LMENYFWAMGCALEPHFWRCRKEITKLVSIITTIDDIYDTYGSIEELVLFTNAVDEWKIIEIQSLPNCMRKALLTLINTMNEIAFAFSKE
KGLDILPQLKRPWGYQCKAYLVEAIWYNTRYIPTLNEYMENAWLSIGTALVLTVAYLLSEDLTKEALNSLELYFDVTRYSCMITRLYD
DLGTSKDELQRGDVPKSIQCYMNETNVLEFVARDHIRQLIKKYWKLLNGEYFSNFNLEESFKRYALNLPRMTQCIY
>DoTPS1
CKEVILCGLRSTAICFQLLRRFGFQISEGIFDDFMDGDHNAFLPTLSDDVIGLLSLYNASYMAFPGENKMESARSFAVKHMNRENMQE
ILPGSIESAIGHALDLPLHRRMPWMEARMYIDMYELEDDMSPPLLHLAKIHFNKVQSIHQKELKHAGSWWRRLDLGKTINFSRDRLM
ECFFYVVGIVHHPDYGFCREKLTQVGRSGHRPGRSVQIKEDRPEHRPGCKTGRLKIGLIVQADLTIILADHFKSEK
>DoTPS33
YKEVILCGLRSTAICFQLLRRFGFQISEGIFDDFMDGDHNTFLPTLSDDLIGLLSLYNASYMAFPGEKKMESARSFALKNMTRKNMQEI
LPGSIESAIGHALDLPLHRRMPWMEARMYIDMYELEDGMSPALLHLAKIHFNKVQSIHQKELKHAGSWWTRLDLGKTISFSRDRLM
ECFFYVVGIVHHPDYGFCREKLTQVGMLIATIDDVYDVYGSLEELELFTQIIDRWDIKGVVELPYYMKICFSALNDTINGVANHLYAD
DRFNILQWADLFKSFLVEAKWHKKGYIPTLKEYLSNASISAAGHVILFLAYILLKCKITKETLQLLQDFPNIIRLPSLIFRLCNDLATSSNI
QVEQERGDVATSVQCYMKETGASEEEARLYISMLISDAWNDMNEEYSTCSKPQFPQRFIDASINLARMASFMY
>DoTPS25

	NNLIENNNLQDFALLFRLVREHGIHNASILRLDDLISYFKKEEESFNLNNQLDVKGMLNLYEASFLAMEGEDELDEAGKIAMKHLKY<br>HDRSLLSLQLVEQIEHALELPLHWRMSRLHTRWFIDAYGRQKNFNPTLLQFAKLDFNMVQNIYKIELQELSSWWRNLNVVRGELNF<br>ARDRLVENYLWTVGYTFQPELWRSRKALTKINSLVTTMDDIYDIYGTLEELKLFTNSIEEWNIDAAQQLPDYMKKCLTVLFNTMNDI<br>ASSFSKDKELDILPCLKRMWADLCKAYFIEAMWYHNGYTPTLDEYLDNAWVTISGICTLITTYCLSDDLTFEAIKSLEFYPPIVQYSSML<br>ARLYNDLGTCTAEIQRGDVPKSIQCYVKEKNVSESAARDYIRCLIRNYWKKLNKEHALSSNYVKSFRKVLVDIPRAAQCFY<br>>DoTPS30<br>NNSIEENNLHGSALLFRLLREQGIKNAVNALISCFKKERENFNQNHEHIVKGMLSLYEASYLAVEGEEELEKAGKLAMDHLKCIDRS<br>LFTQQFIEEIDHALELPLHWRMSRLHTRWFIDAYGRRENFNPTLLELAKLDFNMVQGIYKVELQELSMWWRNLGLVCEELDFVRDRL<br>VENYLMSIGTTFQPNFGRLRKALTKIICFVTTIDDIYDIYGTLDELKLFTNTIEEWKIDASQQLPDCMKICSIAILSTMNDIATSFSMEKAL<br>DILPCLKRAWVDFFKSQLLEAKWYHNGYTPTLDEYLENAWVSISGNIGLTAAYCLSDDLTIESLNSLEFYPHFIRYSCTIVRLYDDLQTS<br>TAEIKRGDVPKSIQCYMKEKNVSESVARDYIKCLIRIYWKKLNQECATISTLWEPFRNDLYGCPRAAQCFY<br>>DoTPS23<br>SNILKNSLHGSALLFRLLREYGIKALNTREDFLVRSFKNENGSFKVHIVNDVKGMLSLYEASYLSVEGEDDLDEAMEFTTKHLSNYLKE<br>PLLIHPSLVEQISHALELPLHWRMPRLHTRWFIDAYERQENMNHSLLEFAKLDFNMVQSICKKEVKEMSSWWRSIGLAGDEFSFARD<br>RLMENYFWTMGCTFEPHFWRCRKEITKLASLITTIDDIYDIYGSVEELMLFTNAVNEWKITEIQSLPNCMRKALLAIINTMNDTACAFS<br>KEKGLDILPQLKQAWGDQCKAYLVEAIWYNTRYIPTLNEYMENAWLSVAISLVINAAYLLSEDLTKEALNSLEFYFDVTRYSSMVTRL<br>YDDLGTSTDELQRGDVPKSIQCYMNETNVSEFVARDHIRQLIKKYWRLFNGEYFSNFNLEESFKRYALNLPRMAQCIY<br>>DoTPS18<br>SNILKNSLHGSALLFRLLREYGIKALNTSEDFLVRSFKNENGSFKVHIVNDVKGMLSLYEASYLSVEGEDDLDEAMEFTIKHLSNYLKE<br>PLLIHPSLVKQISHAIELPLHWRSPRLHTRWFIDAYERQENMNPSLLEFAKLDFNMVQSICKKEVKEMSSWRRSIGLASDEFSFARDRL<br>MENYFWIMGCTFEPHFWRCRKEITKFASLISTIDDIYDIYGSVEELVLFTNAVDEWKIIEIQSLPNCMKTTLLALINTMNDIACAFLKEK<br>GLDILPQLKRAWGDQCKAYLVEAIWYNTRYTPTLNEYMDNAWLSAAVPLVLTAAYLLSEDLTKQALNSLQFYFDVTRYSSMVARLY<br>DDLETSTDELQRGDVPKSIQCYMNETNVLESVARDHIRQLIKKYWKLLNGEYFSNFNLEESFKRYALNLPRMAQCIY
Oryza sativa	>Os01g23530<br>NDKDLNLVSLRFYLLRKNGYNMSSGIFLSFKDNEGNFIVDDTRSLLNLYNAANLRVYGEKVLDEAATFTISRLEGVLESSDSILSEVSFA<br>LEAPIFRRARIVEMRNYIPIYEIEATRNETILEFAKLNFNLLQLLYCEELNKITLWWKELKVKSNLSFSRDRIVEMYFWMNGALYEPHYS

HSRIILTRVTAFMTIIDDIFDTYGTTEESMLLAEAINRWDESAIGLPEYIRGFYAYLLKTFDSFEEELGPEKRYRLKRLVQAYTKELKWRD
EDYTPKTLEEHFEVSMRSSGGFTLAAASFVGMDDIATKDIFEWILSYPSLFKTFDIFVRLSNDIVSNKREQTGDHYASTIQCYMKEHGTT
IHETYQRLRELIEDSWKDMVEHCTDQPLIVPQTVVNFARTVTTMY
>Os01g42610
YSSNLHEVALRFRLLRQQGYWVSPDEFNKFKCEDGSFKSDINNDPKGLLSLYHAAYLLTHNERALKEAILFATHHLELLSGSLEFPLAE
QVKRALQIPLPRTLKRVEALNFIFEWWKDVSSDINLDYTRDRVVECYFCAYIVYYEKEYARARMMLAKKIMLISLLDDTYDVHATLEE
ARKFNEALQRWDKNAVSLVPEGLKRFFLSIMSNFRDFEDELEPHEKYRNAYNIKAFQILSNNYLQEAEWFHQKYIPSFTEHATVSLVT
GGAIELPVSIIVGMGDIATKDAFDWALSYADAGRAFGEVSRFMDDLAVSQNGREKMDVANAVECYMKEHGVTSDVAEAEISEMVE
GAWRTLNQARFVYLPFVQRIANVSMSIALLF
>Os02g02930
AHGGDLLDATLAFRLMREAGHHVSADEVGRFTDDNGEFRLDYRKDIRGLLSLQDISHMNIGQEASLCKAKEFSTRNLESAINYLEPN
LARYVRQSLDHPYHVSLNQYKARHHLSYLQTLPIRCTAMEELALADFQLNKLLHQMEMQEIKRWWMDLGLAQEIPVARDQVQKW
FVWMMTAIQGASLSRCRIELTKIVSFVYIVDDIFDLVGTREELSCFTQAIRMWDLAAADSLPSCMRSCFRALHTVTNDIADMVEREHG
VNPINHLKKAWAMLFDGFMTETKWLSAGQVPDSEEYLRNGVVTSGVPLVFVHLLFMLGHDVSQNAAEFVDHIPPVISCPAKILRLW
DDLGSAKDEAQEGLDGSYKELYLKENGLAAGEAEEHVRRLIAGEWEELNRECFTFPAGFTQAALNAARMVGVMY
>Os02g17780
ARNSNVKEVDDTAMAFRLLRLHGYNVSPSVFKNFEKDGEFFCFVGQSTQAVTGMYNLNRASQISFPGEDILQRARNFSYEFLREREA
QGLHDKWIISKDLPGEVQYTLDFPWYASLPRVEARTYIGQYGGNDDVWIGKTLYRMPIVNNATYLELAKQDFNRCQALHQHELQG
LQKWFIENGLEAFGMTPEDVLRAYFLAAACIFEPNRASERLAWARVSVLANTISRHFDMSSMKRMERFMWSSEENGNLGGYAGILA
RTLCQLIDLLSQETPPVREGQKCIHNLIRCAWIEWMMQQINMKDGRYDKGRVMHPGSCTVHNKETCLLIAQIVEICAGREMINNTE
GSWFIQLASSICDSLHAKMLLSQDKKNETTINQIDKEIELGMQELAQYLLVDDRRINNKKQTFLSIVKSCYY
>Os02g36140
KDEEIMLDVTTCAMAFRLLRMNGYDVSSDELSHVAGASGFRDSLQGYLNDRKSVLEVYKTSKHSISENDLILDSIGSWSGSLLKEMLC
SNGKGTPGREEIEFALKYPFYSTLERLVHRKNIVLFDAKGSQMLKTECMPVHDSQDFLALAVDDFCISQSNYQNELNYLESWVKDNR
LDQLHFARQKITYCYLSGAATTFRPEMGYARTSWARTAWLTAVIDDLFDVGGLEQEQENLLALMEKWEEPGEDEYSEDVKIVFQAL
YNTVNEIGAKASALQGHDVTKYLVDVWLHVVRCMKVEAEWQRSQHLPTFEEYMESGMVSLGQGCTVMSALFLIGEKLPEGIVELEE

YDELFRLMGTCGRLLNDIRGIEREESDGKMTNGVSLLVHASGMSVDEAKTEVMKRIDASRRKLLSLVVPIPRPCKQLFWKMCKILHLF
Y
>Os02g36210
GGLCPVKDIDDTAMAFRLLRLHGYNVSSSVFNHFEKDGEYFCFAGQSSQSLTAMYNSYRASQIVFPGDDDGLEQLRAYCRAFLEERR
ATGLMDKWVIANGLPSEVEYALDFPWKASLPRVETRVYLEQYGASEDAWIGKGLYRMTLVNNDLYLEAAKADFTNFQRLSRLEWLS
LKRWYIRNNLQAHGVTEQSVLRAYFLAAANIFEPNRAAERLGWARTAILAEAIASHLRQYSANGAADGMTERLIDWDWESKDSAA
RSLLYALDELIDLHAFGNASDSLREAWKQWLMSWTNESQGSTGGDTALLLVRTIEICSGHGSAEQSLKNSADYARLEQIASSMCSKL
AKILAQNGGSMDNVGIDQEVDVEMKELIQRVYGSSSNDSVTFLDVVKSFCYV
>Os02g36220
NEEELMLDMGTCAMAFRLLRMHGYDISSDGMAQFVEQSSFDDSIHGYLNDTKALLELYRSSQIRCLEDDLILQDIGSWSARVLQEKIS
SKMTHKSEMLEVEYALKFPVYATLERLEQKRNIEQFKTKEQLKIEGFKLLKSGYRGAITHDEILALAVDEFHSSQSVYQQELQDLNSW
VAQTRLDELKFARLMPSITYFSAAATMFPSELSEARIAWTQNCILTTTVDDFFDGDGSKEEMENLVKLIEKWDGHGEIGSSECVEILFY
AIYNTSKQIAEKAVPLQKRNVVDHIAESWWFTVRGMLTEAEWRMDKYVPTTVEEYMSAAVDSFALGPTITSAALFVGPELSEEVFRS
KEYIHLMNLANTIGRLLNDMQTYEKEIKMGKVNSVMLHALRGSEASMEEAKREMRRVLQGSRCDLLRLVTVVPPPCRKLFWFMSK
VLHFVY
>Os02g36264
NEEELMLDMRTCAMAFRLLRMHGYDITSDGMAQFVEQSSFDDSIHGYLNDTKALLELYKSSQLRCLEDDLILEEIGSWSARVLLEKISS
KMIHISELPEVEYALKCPVYAILERLEQKRNIEQFKTKEQLKIEGFKLLKSGYRGVIPNDEILALAVDEFHSSQSVYQQELQDLNSWVAH
TRLDELKFARLMPSITYFSAAAVLLPSESARIAWTQNCILTTTVDDFFDGEGSKEEMENLVKLIEKWDDHGEIGSSECVEILFYAVYNTS
KQIAEKAMPLQKRNAVDHIAESWWFTVRGMLTEAEWRMDKYVPTTVEEYMSAAVDSFAVGPIITSAALFVGPELSEEVFRSEEYIHL
MNLANTIGRLLNDMQTYEKEIKMGKVNSVMLHALRGSEASMEEAKREMRRVLQGCRFELLRLVTVVPPPCRKLFWLMSKVLHFVY
>Os03g22634
PSLHDVALRFRLLRQQGLWVSSDVFNKFKHRDGSFIIDITNDPKGLLSLYNAANLLTHNEEALQEAILFSRHHLELMKSNLKSPLAEQ
VSRALQIPLSRNLKRVEALSYILEYNVHEQTYNPSILELAKLDFNLLQHIHQRELKTITQWWEDLSNDIGLDYIRDRIVECYFWSYSMYF
EEEYTRARMILAKFFMLTSLLDDTYDTHATLEECRNLNVAIQSWDESDISVLPDYLKKFFLKVMSNFVEFENELEPHIRHRNAYNRKV
FQLLSGYYLQEAEWFHHNYVPSFKEQIEVSVMSAVTEETLEWAIGNNDAVRAGGEVARFMDDMAAFKNGRNKLDVASSVECYIKE

YNVTSEVALAKIGSLVEDAWKTINQAHIRELLPFVHRVTNLSRSMAILF
>Os03g24640
DFAGSDDDLYTVALRFRLLRQHGVWVSACVRDKFRDGTCSFSSSLRDDPRGLLSLYNAAHMAAPVEIALDDIIAFARCHLEALSMEG
ELKSPLAEQVSRAPDIPLPRFPRRLETMSYLVEYEQEDEHDDMLLELARLEFELTRCLHLEELKALSLWWRELYESVKLSYARDRLVES
YFWTCGVFHEEEYSRARIMFAKVFGLLSLMDDTYDVHATLEECYKLNEAIQRWDEGAISILPEYLRMFYIKLLSNFDELEASLEPHEKF
RVSYAKNAFKLSSEYYLREAKWSNTKYTPSFAEHLEVSVMSSGFPMLAPVVLMGVHDVATAAAFEWATGVPDVVIAASGEVARFLN
DIASHRVGKNEKDVPSSVESYMAEHGVGEEAALAAVAAMAEHGWRTINRALMEMDPGLLPAIVNLTRTLEVIY
>Os03g24680
GSDVADDDLHVTSLRFRLLRQHGLGISADVFDKFRDNGGSFRASLSSDTRGLLSLYNAAHLAMPGEEVLDDAIAFSRRHLRSMKTAG
KLRSPMAEQVSRALDIPLPRTPRRLEAMRYIHEYGDEPGFDGVVLELARLDFELVKSLHLRELKALTLWWKDFYDNVKLSYTRDRIAE
VFFWVSGVYYEEKYSRARIMLAKVFGLITLMDDTYDVQDTLDECCRFNETIQRWDNGAVSILPEYMHAYYIKLLSNFDEMENSLEPN
EKHRVSYAITMYKQLSEYYLQEARWSSHRYLPSFAEHLYVSSISSGIPALAPAVLMGVHDVATKEALEWACAIPDLLLASGEEGRASL
VETYMTEHGAGGDAAVAAVAAASERAWRRINRACVAVEPALLPALVNLTSTMEVVY
>Os03g24690
AGFDDGDQLYLESLRFRLLRQHGFWVSADVFDKFKDSTGCFRESLSTDARGLLSLYNAAHLAMPGEAALDDAIAFSRRSLQSLQGAL
RSPMAKQVSRALDIPLPRAPKLLETMHYITEYEQEAAHDGMVLELARLDFELVRSLYLKELKALSLWWRQLYDSVQLSYARDCLVES
YFWTCAMFHGEDYSRARIIFAKVFQLMTMTDDIYDIHATLEECYKFNEAVQRWDKSAVSILPEYLRNFYIRILNDFDEMEDSLEPDEK
HRMSYVKSSFKQQSEYYLREAQWSSDKHMPSFAEHLDVSFMSIGYPTMAVVVLLCARDAASMEASEWAPSLVRAGGEVTRFLNDIA
SYKTGKSGKDAASTIECYMAERGVGGEEAVAAVAALVESAWRTINRACVEMDPNLLPALVNLATTPEVIY
>Os03g31430
ANDDFHAISLQFRLLRQQRRYMPCDAFKEFIDKQGNLNGTLCSDTRALLALYEAAHLGTPNEEILREAQVETTNQLKRIVDCIEKPLS
NKVRHALETPSFRRMKRLEARLYIPLYEEDKEECNEMILELAKLDFYLLQRLHREEVKEICEWYHGLESPRELFYARHRPAEAYFWAL
GVYYEPEYAKPRKLLAKFIATITPYDDTFDNYGLWKELQPFADVMQRWDEKGAEQLGRCYKEYAQFMFGTMNEIEGALPKGTPRKN
VNVIKDIITEVCKGYVTEIDWRDSKYIPPLKEHLQITLVTCFYWAINCTAFVVFQEGVTEEVMIWMSGFPQIVKDSCIVSRLMDDIVAH
AFETERNNVATAVTCYMKEYDSTKEEAIKALWNDVENAWKDMNEEYLSIPSSLLIQVSIMNNRVSKLAS
>Os04g09900

TKNCPVKDIDDTAMGFRLLRLYGYQVDPCVKKFEKDGKFFCLHGESNPSVTPMYNTYRASQLKFPGDDGVLGRAEVFCRSFLQDRR
GSNMKDKWAIAKDIPGEVEYAMDYPWKASLPRIETRLYLDQYGGSGDVWIGKVLHRMTLFCNDLYLKAAKADFSNFQKECRVELN
GLRRWYLRSNLEKFGGTDPQTLMTSYFLASANIFEANRAAERLGWARVALLADAVSSHFRNSTSNEELISLVPFDDAYSGSLREAWK
QWLMAWTAKESSQESIEGDTAILLVRAIEIFGGRVLTGQRPDLWEYSQLEQLTSSICCKLSRVLAQENGESTEKVEEIDQQVDLEMQEL
TRRVLQGCSAINRLTRETFLHVVKSFCYV
>Os04g10060
RDEEVMLDLPTCAMAFRLLRMNGYGVSSDDLSHVAEASTFHNSVEGYLDDTKSLLELYKASKVSLSENEPILEKMGCWSGSLLKEKL
CSDDIRGTPILREVEYALKFPFYATLEPLDHKWNIENFDARAYQKIKTKNMPCHVNEDLLALAAEDFSFCQSTYQNEIQHLESWEKEN
KLDQLEFTRKNLINSYLSAAATISPYELSDARIACAKSIALTLVADDFFDVGSSKEEQENLISLVEKWDQYHKVEYSENVKAVFFALYST
VNQLGAMASAVQNRDVTKYNVESWLDYLRSLATDAEWQRSKYVPTMEEYMKNSIVTFALGPTILIALYFMGQNLWEDIVKNAEYD
ELFRLMNTCGRLQNDIQSFERECKDGKLNSVSLLVLDSDMSVEEAKEAINESISSCRRELLRLVVVIPKSCKEMFWNLYKTSHVFY
>Os04g26960
TSSNLHEVALRFRLLRERGFWVSPDIFNKFKGDDGNFLNEFAEDPRSLLSLYNAAHLFIHGEPELEEAISFARNHLESMSSHSVLKAPL
ADQVKRHLRLPLPRTHKRVEMLHYMFEYDQENEHNPVLLELAKLDFNLLQQVHLKELKEISRWWKDVLAYMGLDHIRDRVIECYT
WSYAVYHEKDLALARMIFAKLVALTSVLDDTYDVHASIEECRMLNVAIQGWDDSAALLVPEYLRKFYEIILRTFREFEDQIPRNQRYL
AAFSKAEFQKLTSNYLEAAEWYHRNHKPSFNDQVALGTATTGTRSLAAGLMLGMGDATTKQAFQWAVTSTDAIISCGKIGRLMND
ISGFKLGQNKADMACAVEAYIEEHKVTADVAIARINEVLEDEWKTTNQARVAVLPVVQRMTLGIQLFY
>Os04g27070
ASSKLHEVALRFRLLREHGFWVSPDVFNKFKGDHGTFNNELSLADDPRGLLSLYNAAHLFIHGEPELVEAISFARHHLESFNRRNVLK
APLADQVKRALHLPLPRTHRRVEMVSYMFEYGREDGHNPVILELAKLDFNLLQRVHLKELKEISRWWKDVSGYMGINHIRDRVIEC
YTWSYAVYHEEEMSFARMLFAKIVVIALLDDTYDVHGSIQECRMLNAAIQGWDDSAVLLVPEYLRKFYEFILRCFREFEDQVPSNQK
YLIAFSKTELQRLSSYYLEGAEWSHRKHMPSFSEQVALATMTTGTRPLAAGLMVGMSEMTTKQAYEWAVNSTDAIISCGKTGRFMN
DIAGFKLGQNKADMPCSVESYINEHKVTADVAIAKINELVEDEWKTTNQARILPVVQRLINITMAIPLYY
>Os04g27190
TSSSLHEVALHFRLLREHGIWVSPDVFEKFKGEDGRFINTIADEPRALLSLYNAAHLLVHDEPELEEAMSFARHHLESMRDGSRLKAP
LDNQINRALHLPLPRTYKRVEMLHYMLEYGQEEECIVVLLDLAKLEFNLLQHVHLKELKAFSQWWKDLYGYVELSHVRDRAVESYL

WSYALFYEENLTLTRMILAKIIVFIVLMDDTYDDHATIEECRKLNEAIQRWDESAISLLPEYMKKFYRALLNYFRETEAQVEASDKYRV
TCMKKEFQNLSTYYLQEFEWLHQNYKPAFKERVALSTLSSTVPLLCVTAAVGQGDAVTKESFELTTVRSSAVIACAKIMRFMNDIAAF
KSGKNKGDAANTVECYINEHKVTSEVALDKIESMIESEWRTLNQVQKQFHVVQRVMNLAVAVPFFY
>Os04g27340
TSSSLHEVALHFRLLREHGIWVSPDVFEKFKGEDGRFINTIADEPRALLSLYNAAHLLVHDEPELEEAMSFARHHLESMRDGSRLKAP
LDNQINRALHLPLPRTYKRVEMLHYMLEYGQEEECIVVLLDLAKLEFNLLQHVHLKELKAFSQWWKDLYGYVELSHVRDRAVESYL
WSYALFYEENLTLTRMILAKIIVFIVLMDDTYDDHATIEECRKLNEAIQRWDESAISLLPEYMKKFYRALQNYFRETEAQVEASDKYRV
TCMKKEFQNLSTYYLQEFEWLHQNYKPAFKERVALSTLSSTVPLLCVTAAVGQGDAVTKESFELTTVRSSAVIACAKIMRFMNDIAAF
KSGKNKGDAANTVECYMNENKVTSEVALDKIESMIESEWRTLNQVHQQFPVVQRLLNLAVSVPFFY
>Os04g27400
ISSSLHDVALRFRLLRQHGFHVSPDVFNKFKGDDGRFVSGITNDPRGLLSLYNAAHLLTHDEPELEEAISFATQHLASLSSGTDLNPHL
IDQINRALDVPLPRTYRRMETLCYMPEYRQEEGHIPILLELAMLDFNLLQHVHLKELKAISEWWKDLYGYMGLSYIRDRVVESYVWSY
VVFYEEDSALARMIFTKIIAFIILMDDTYDSYATIQECRKLNEAIQRWDESATALPEYIKKFYSALLKTFKEFEIHVEDDGQYRIDHTKKA
FQNLSAYYLQEAEWSYQNYKPSFEEQVALSTVTSTVPLLCVSTTVGRGDALTNEAFEWAANDIGAKIACAKITRFMNDIAAFQRGKN
RGDVVSTVECYMNENKVTSEGAFTKIDLMIEDEWRTINQALCLPAVQQVLNLAICATFFY
>Os04g27540
TSSSLHEVALRFHLLREHGLWVSPDVFNKFKADDGKFIDEHNDLKQPLFDKFSRALHLPLPRTYKRVETLHYFLEYGQEEGHIPILLDL
TKLDFNILQRVHLKELKAISEWWKDLYKYIGLTYISDRAVESYIWSHTMLFGEGLALTRMICAKIIILLVIMDDTYDAHATIEESRKLNE
AIQRWDESAIPRVPEYLKKFYIKLLNNFKEIEDQVMDNEKYKVAYAKKEFQKLSHYYLQEVEWLHQNHKPSFQEQVDLSTKTSTAHL
MFVSTTVGLGDAVTKEALEWAESSTAIVAVGKIMRFMNDIAAFKHGKNKGDVTSTMECYMNEHKVISDVAFMKLTSLIEHEYRTIN
QAELSLPAAQRVVVVSLMFFY
>Os04g27720
TSSSLYEVALRFRLLREHGFWVPPDAFNKFKGDDGRFRNEIANDPRGLLSLYNAAHLLIHGEPELEEAITFAREHLKLMSQDNVLNPP
LACQVRRALTLPLPRTFKRVETICYMLEYQLEEGNIPILLDLARLDFNLLQHIHLKELKAISEWWKDLYGYMGLSYIRDRTIEGYTWSY
MMFYEEGFAFTRMFVAKLIALCRWDKSAISILPEYLKKYYSKLLINFKEFQDQVTDNEKYMVACTKEEFQKQSTYYLQEAEWSNQKY
KPGFKDQVVLSTKSSAVQLLCVAAMVGWGGTMTTEAFEWVASGNAAVIACAKIGRFMNDIAAFKRGKNKRDVASSVECYMNENG

VTSEAAFAKINALVEDEWRSTNQTRLTLLPMVQRIVNFTVSMALFY
>Os04g27790
TSSSLHEVALRFRLLREHGLWVSPVTFNKFKGDDGRFMNGIADEPRGLLSLYNAAYLLVHDEPELEEAISFSRYHLKSMMQGNNLKH
PLSDQVKRALNTPLPRTSKRTETLHYLSEYGQEEGHMSILLDLAKVEFNLLQGVHLKELKAISEWWRDLNEHVELSYLRDRVVESYTC
SHMLFYEEGLAFTRITFTKIIVLIIMMDDTYDSHATIQECRKLNEAIQRWNESAVSVLPEYLKNFYHKLLNNFKEFENQVVVSEKYRVA
HAKKEFQILSHYFLQEAEWSHNNYKPSFEEQLALSTKTSTVQLLCVSTTVGRGDAITNEAFMWAASSTTVTSCAKIMRFMNDIASFER
GKNKGEIASTVECYMNEHNIISEVAFAKLDSLVEDEWRTINQARCQLLPVVQRVVNLAICIMFFY
>Os04g52210
RDEEVMLDITTCAMAFRILRMNGYDVSSDDLCHIAEVSDFHSSHQGYLSDTRTLLELYKASEVSVADNEFILDRIGSWSGRLLKEQLSS
GALQRTSSIFEEVEHALDCPFYATLDRLVHKRNIEHFAAMSYISYAQNNIPDELERIDSWVKENRLHELKFARQKSAYFYLSAAGTVFD
PEMSDARIWWAINGVLTTVVDDFFDVGGSREELENLISLVEMWDEHHKEEYSEQVEIVFFAIFNSVNQLGAKVSAVQGRDVTKHLIEI
WLDLLRSMMTEVEWRISNYVPTPEEYMENAAMTFALGPIVLPALYLVGPKIPESVVRDSEYNELFRLMSTCGRLLNDVQTYEREDGE
GKVNSVSLLVIQSGVSIEEARREIMKPIERCRRELLGLVLAVPGPCKELFWKMCKVCYFFY
>Os04g52230
RDEEIMLDITTCAMAFRLLRMNGYHVSSVELSPVAEASSFRESLQGYLNDKKSLIELYKASKVSKSENESILDSIGSWSGSLLKESVCSNG
VKKAPIFEEMKYALKFPFYTTLDRLDHKRNIERFDAKDSQMLKTEYLLPHANQDILALAVEDFSSSQSIYQDELNYLECWVKDEKLDQ
LPFARQKLTYCYLSAAATIFPRELSEARIAWAKNGVLTTVVDDFFDLGGSKEELENLIALVEKWDGHQEEYSEQVRIVFSAIYTTVNQL
GAKASALQGRDVTKHLTEIWLCLMRSMMTEAEWQRTKYVPTMEEYMANAVVSFALGPIVLPTLYFVGPKLQEDVVRDHEYNELFR
LMSTCGRLLNDSQGFERESLEGKLNSVSLLVHHSGISIDEAKMKAQKSIDTSRRNLLRLVLAVPRPCKQLFWKMCKIVHMFY
>Os04g52240
RDEEIVLDMQTCGMAFRMLRMNGYDVSSDELSHFSEPSSFHNSLQGYLNDTRSLLELHKASKVSIAEKEVEYALEFPFYTILDRLDHKR
NIEHFDITSSQMLETAYLPCHSNEEIMALGVRDFSSSQFIFQEELQQLNSWVKESRLDQLQFARQKLDYFYFSAAATIFTPELSDVRILW
AKNGVLTTVVDDFFDVGGSKEELENLVALVEKWDKNDKTEYSEQVEIVFSAIYTSTNQLGSMASVVQGRDVTKHLVEISDILWNYFFS
EVRTDKLNIVASVNLCIRIAEVYDDRGRVEAEPHCILLGPKMPDSVIRSQECSELFRLMSKCGRLLNDVQSYEREGSQGKLNSVSLLAL
HSGVSMEEAVKQIQRPIEKCRRELLKLVAVPRPCRELFWSMCKVCHFFY
>Os07g11790

NSSDLYEVALRFRLLRKQGYWVSPDEFNKFKAEDGSFSSDDITNDPKGLLSLYNAAHLLTHNEKALEEAILFARHHLQLLRGNLAYPL
DEQVTRALEIPLPRTMKRVEVLNYIFEYSAEEKMFNPSILELAVLDFNILQKVHQNELKEICQWWENLSSDIRLDYVRERVVECYFCAY
AAYYEKEHARARMIFAKRCMLFSLLDDTYDVRATLEEARKFNDALQRWDKSDVSLLPEDLKRFFLSIISNFREFEDELEPHEKYRNSY
NIKAFQILSSNFLQEAEWFHQNYIPCFTDHVTVSLQTGGAIELPVSLIVGMGDIATKEVLDWALANPDAGRAFAEVARFMDDLAASH
SGRDKMDVASTVECYMNEHGVTREVAEAKIAGMAEDGWKSMNQIRFAFLPFVQRIANLCMSATLLY
>Os08g04500
DTDNHYDLHTTALRFYLLRKHGYYASPDVFQRFRDEEGNFTRDDTRSMLSLYNAAHLRIHGEEILDDAIVFTRNYLQSVVKHLQSPM
ADEVCSALRTPLFRRPRRVEARHYISVYDKLPTRNETILEFAKLDFGILQSLYCEELNILTMWWKELQLQDHLSFARDRMVEMHFWM
LGVLFEPQYSYGRTMLTKLFIFVSIFDDIYDNYSTLEESKLFTEAIERWDEEAAEELPGYMKFFYKKVLTTMKSIETDLKLQGNKHVDYV
KNLLIDATRCFYNEVKWRSEDQVAATVEEHLKISVPSSCCMHVPVYAFVAMGDVTTDDAINWGMAYPKIITSSCIVGRLLNDIASHE
REQGSSSSSSSVEACMREHGITKEEAYAKLRELVEESWMDIAGECLAQPPPLLEAVVNATRVLDFVY
>Os08g07080
NDKDLHLVSLRFYLLRKNGYDVSSDMFQHFKDKEGSFVADDVRSLLSLYNAAFLRTHGEKVLDEAIVFTTNRLRSELEHLKSPAADE
VSLALNTPLFRRVRILEIRNYIPIYESATTRNESILEFAKLNFNLLQLIYCEELKSITGWWKELNVESNLSFIRDRIVEMHFWMIGACSEPH
YSLSRIILTKMIAFITILDDIFDTYATTEESMMLAKAIYMCNETATVLLPKYMKDFYLYYLKTFDSFEEALCPNKSYRVCYLKELFKRLVQ
EFSQEIKWRDDHYIPKTIEEHLELSRKTVGAFELACASFVGMGDLVAKETLDCLLTYPELLKSFTTCVRLSNDIASTKREAGDHHHAST
IQSYMLQHGATAHEACVGIKELIEDSWKDMMKEYLDLQPKIVARVIDFARTGDYMY
>Os08g07100
NDKDLHLVSLRFYLLRKNGYDVSSDIFQHFKDKEGSFVADDTRSLLSLYNAAYMRTHGEKVLDEAVVFTTNRLRSELKHLKSPVADE
VSLALDTPLFRRVRIIETQNYIPIYESATTRNEAILEFAKLNVNLLQLIYCEELKTITRWWKELNVESNLSFIRDRIVEMHFWMTGACSEP
HYSLLRIILTKMTAFITILDDIFDTYATTEESMMLAKAIYMCNESATVLLPKYMKDFYLYYLKTFDSFEEALGPNKSYRVLYFKELREQA
GDHYASTIQCYMLQHGTTIHEACIGIKELIEDSWKDMMKEYLNLQPKIVARVIDFARTGDYIY
>Os11g28530
DEEEIMLDMATCAKAFRLLRMHGYDVSSEGMARFAERSSFDDSIHAYLNDTKPLLELYKSSQVHFLEEDFILENIGSWSAKLLKQQLSF
NKISKSLMPEVEYALKYPFYATVEVLEHKGNIERFNVNGFQRLKSGYCGSGADKEILALAVNKFHYAQSVYQQELRYLESWVAEFRL
DELKFARVIPLQSLLSAVVPLFPCELSDARIAWSQNAILTAVVDDLFDGGGSMEEMLNLVALFDKWDDHGEIGCSSNVEIMFNAVYN

	TTKRIGAKAALVQKRCVIDHIAEQWQVMVRAMLTEAEWAAGKHIPATMGEYMSVAEPSFALGPIVPVSAYLLGEELPEEAVRSPEY<br>GRLLGLASAVGRLLNDVMTYEKEMGTGKLNSVVLLQPASRGASVEAARAEVRRAIQASWRDLHGLVFIIPRPCREVFWHTGKVASV<br>FY<br>>Os12g30824<br>NEEEVMLDIPTCAMAFRLLRTHGYDITSDEMAHFSEQSSFDDSIHGYLNDTKTLLELFKTSQIRFSCEDLVLENIGTWSAKLLKQQLLS<br>NKLSTSAQEVEYVLKFPLHSTLDRLEHRRNIEQFKVEGSKVLKSGYCGSHSNEEILALAVDYFHSSQSVYQQELKYFESWVKQCRLDEL<br>KFARVMPLIVHFSSAATIFAPELADARMVLSQTCMLITVYDDFFDCPESREEKENYIALIEKWDNHAEIGCSKNVEIVFYAVYNTYKQI<br>GEKAALKQNRSIMDQLVEDLVSSAKAMMVEADWTATKYIPATMEEYMSNAEVSGAFASFVCPPLYFLGLKLSEEDVKSHEYTQLLK<br>LTNVIGRLQNDSQTYRKEILAGKVNSVLLRALTDSGNTSPESIEAAKEIVNRDAESSMVESLPIPRPCKDRFWEMCKIVFYFY
Phalaenopsis equestris	>Peq011991<br>YDDNDLYTVALRFRLLRQQRYNILADVFNKFLDEKGDIKDCFSSDAKALLSLYEAGNLALPTEEMLDKAVNFSKSRLMSMKSELEPH<br>FALIVSSSIDIPLFKRTDRIKTRSYLSIYEQVSDFNQVLLEFAKMDFNCLQALHQEEARKITLWWKNLGLAERLPFARDRLVECYFWVLS<br>VYFEPRYSRARLMMTKCITQMSFLDDVYDVYGTLEELEILTHAIQGWDIESASKLPEYMQHSFLSLINTFEGMETELAPEHNSFRIQYLK<br>NELQKIARAYLQEAKWAKTSYVPKLEEHLEISLITAGYAFLTCASYVGMEEVIPKDVFDWVMSFPEIIKASCIIGRLMNDIVSYKLEQER<br>NHVASAVQCIVMEQECSEEEACKKVLEMSNNAWKVINKEFAMSANLPFYLVWPIINLARFNEFIY<br>>Peq011994<br>YDENDLYTVALRFRLLRQQRYHISSDVFSKFLDEKGDFKDCFSSDAKALLSLYDAAHLGMATEEILDKAINFSKTHLISMKSQLEPHFS<br>LMVSSSLEIPLFKRTDRIKTRNYLSIYEQDTKFNEALLEFGKFDFICLQAMHQEEARKLSMWWKNLGLSKRLPFSRDRLVECYFWVLSV<br>YFEPYYSRARIIMTKCITQMSFLDDIYDVYGTVEELHLLTDAIEGWDIKLIDKLPEYMQHSFRSLFETFKEIEIELAPEQSSFRVQYLIYELK<br>KVSKAYLQEAIWANKCYVPKLEEHFGASLLTAGYSFLTCASYMGMKEAISKEVFDWVISLPEIVMSSCVIGRLMNDIVSFEHERKRTH<br>VASAVQSYAMEHGCSEEEACNKLTEMSNNAWKSINKEFVELSNLPLSLVWPIVNLARFNEFIY<br>>Peq021785<br>KDEEIYLNIATCAMSFRLLRLHGYDVSSDALAPFADMRHFKNTLQGYIRDLNAIIELYKASQVQISPEEAVLKKLNSWVTHFLKEELNT<br>NANHSLDILQEVDHTLGFPFYASMERLEHKLNIEHYNSDKLHMLKSSFVYSMKNNDLLKLAVHTFNTSQLTYKKELQYLESWVKES<br>KFNQLGFSRQKQIYCYLSAVMTFFSSETSSARICWAQCSTLITVIDDFFDFGGSSEELANLIKLVEKYALSLFSFVVAIMKNIFVIDGSIFGR<br>WHGVQEKDFCSENVKIIFFALYNTINELGAKASLFQKRDVTKDIINVWLVLLSSEMKEAEWRQNSTFPTFDEYMTSAIPSFALGPIILPSI

YLAVPELLENIIGSPEYKNLLKLLGKFGRLINDIQGYQREKATGKLNSLWLIVHHGNGSISEEDARKHMWSLIDSTRAELLGLVLQNK
GSVVPRACKEVFWKTCKAAYIFY
>Peq014251
RDILHNNLDGSALLFRLLREYGIRVPNPSEDFLIKCFKNESGSLKVNIINDVKGMLSLYEASYLANDDEDELDEAKKFTNKNLSIYLKEP
VLINHSLVEQIRHALELPLHWRMPRLHTRWFIDAYGKQEKMNPHLLKLAKLDFNMLQSIYKKELKEMSRWWKSLGLAGGDFSFAR
DRLMENYFWSMGCTFEPHFWRCRKEITKIGSFLTTIDDIYDIYGSLEELMIFTDVVNEWKTTGNEKLPNNMKKTLSALFNTMNDIAAV
TLAERGLDILPRLKRLWRDQCKAYLVEAIWYHTRYTPTFNEYMENAWITIGVPLLLTCAYCLSEDLTLEALNCLDFYPKVTRYSSMITR
LYDDLATSTDELQRGDVPKSIQCYMMEANVSESVAREHMKYLIEKYWKLLNYEYLHNLNMEECFKRHVLNFPRVTQFLY
>Peq010503
YGESHEINLQAAALKFRLLRQHGYRVTSDVFRKYKDEEGEFRSDLADDVRGLLSLYEACFMATHEDDILNEALSFTEHHLQRLSLEKE
DLQHESPALKKLISQALELPLHRRIPRLGARYYIDAYEHDKEQRNDTILELAKLDFHLLQLLHYEEARSLSIWWKGIGCSEIFKFSRDRI
VECYFWILSVYFEPQYSKARKITTKVIALLSITDDIYDAYGTSDELQSFTNVIQRWDMEAAEQLKEYLKIQFHNLNRAFQEFEDELSSNG
KSYRVHYLKEILKVVVRAWNMEVKWRNEGYIPALKEHLQVTTVTTCYNLLTCASLIGMGDEVTIEAFDWISSFPEFTYQASLICRLRD
DVVSQEFEQKRNHVASAVQCYMNEHNSTLDDSCKMLLKLVESSWKSLNHEYLSLSKTFTKAVLMRVINLARVMETAY
>Peq008177
ARNSEVRDIDDTAMGFRLLRLHGYSVSPDVFRNFKRDDKFFGFIGQSTQAVTGMYNLNRASQLIFSGEEILNQAKNFSYQFLREKQAS
NLLLDKWVISKDLPGEVAYALDFPFYASLPRVESRWYIEQYGGDDDVWIGKSLYRMLYVNNAVYLELAKADFNQCQAIHKLEWLSL
QRWFEACVRKEYGMRQKNVLRAYFLASASKFEPDRSAERLCWAGTAALAQAVVVSYNDSSATNKVEHCSTRPDPDHQCRSKQTAE
ELVGHILMLLDRPLLLRVPAQTFRHHLRRAWEEWLVKLEEGESRGETALLLVRSIELCAGRTEPEGGAARLEYDKLVRLTISICGRLPV
HEGNKIRIADDSHLESDMQQLIKCVLQPESPDGLSGPTKQTFLAVIKSIYYLAWCPPAALDDHITKVLFERVG
>Peq023158
FDSNDLYAVALHFRILRQQRYYISCDVFEKFCEEKGEFRCSLSSNVNALLSLYEAAYLSLPGEEILDKAKIFSKPLLKTSLCNLEKHLAM
MVSAALDVPLVRRVDRPRTKLYLSIYKNDSLFNKLLLDFAKLDFFILQTLYQEEVRSLSIWWKDLGLAKCFPFSRDRIVELSFWLISVHH
EPFLSRVRNMLTKLLALLSLTDDIYDNYGTVEELELFTNVIKSWEIEAVSMLPKYMQPCFLAFHKTINEFERELEQEQYSFRVQYLIEEIK
KMAQAYYEEAKWSSELYVPKLKDQLKASLATAGHSVISCASYMAMREDIPENLFEWVTSMPAIMTSSSLIGRVMNDLASCEFDQSKG
QVASTVQCYMVEHKSSKEEARMKLMEMVEEGWKIINREFMFSTISIALIQTIINITNFNVLVY

>Peq022099
KDEEIYSNVATCAMTFRLLRMHGYDVSSNALSPFVDVRHFKNTVQGYIGDLNAAIELYKASQVQISRDEPILKKLNSWVTYFLKEELN
TNSINSLDISQEVDYTLRFPFYASLERLEHKRNIECSNLDNLQMLKTSFVASMNNNDLKELAVDAFSSSQLICRKELQYLESWVKESKL
DQLGFSRQKHMYCYLSAASTLFSSELSSVRLCWAKGGVLTTVVDDFFDGGGSSEELANLMKLVEKWHEIHEKDFCSENVKIIFFALYN
TINELGAKAFVIQKRDVTNHMIDIWLTMMNSMMKEADWLKNNIVPSLDEYMTHAIPSFALGPIILPSLYFVGPELSENIITHSDYYKLY
ELVSKVGRLSNDIQGFERDKAQGKLNSLSLVLHGNGSVSEEDVRRDAWRMIDSTRAELLGLVLQNEGSLLPRACKELFWKMSKILHF
FY
>Peq027069
RMTNDAYVMAMYFRLSREHGLVASKDLLVQCYKDENGSYKPYVLHDTKGILSLYEASFLAMDGEDELDELKEFTLKQLRYLKTSNSS
TNPKLSEYIAKALELPLHHRIPKLQAPIFIRLARDIEEVNPDPIVIEFAQLDFNMTQSIYIRELKEITSYV
>Peq017060
GDGEVNPSAYDTAWIARIPSIDDPTKPLFPRTLEWIMKNQLEDGSWGESTFFSLYDRLVCTLSCIITLTKWSQGDELIARGLHFLQTRIQ
DLGNEKSVRTVGFEMIFPSMLNEAKTLGLHLPYHLPCMKHIIKLREEKMRRIPMRLMHSVQTTLLHSLEAIDANLVQWDRILKLQCA
NGSMWDSPSATVATYLNTNDEKCFEYLTYIVKRFKDHAPFTYPLDTFERGWMIDTIQRLGIDHHFREEIFDTLDYLYRYLRKDGLGWE
REAVVTDIDDTCMGLRLLRLHGYPVSSDVLEYFKDNNDTFLCFMGETHKGVSEFFNLYRFSQIAFPGEKILKEAKKFAEQHLKIGIND
NQAYDKWAIKKALQMEIEWGLRNEWKMSLPRLDAQEYIRNYGENDVWIGKSVYRWKPALVQVLPTKLKRVFMGVYDTMNELETV
AKNAQGKDIISYLHDLRKKQVQQYLYTREVKDTKQIESFEEYVDHVKNGLAVAIRLVPAMFLVRDALSAKALEDMNLVTKDRVAVR
LYMKEQICPEQDVVSYLSEKVDDAFAELTHEYLKPNKYTPHSYLRLIFEHGRIIRFHL
>Peq017061
GSDASVTDLDDTCMGLRLLRLHGYPISSDMLEYFRDDEGNFLCLKGETHKGISDFFSLYRFSQIAFPGENILKHAKKLAEQHLMKNIK
GRQAFDKWAIKKALHQEVEWALRNQWMKCLPRLEAQEYIRNYGENDVWIGNTIYRMYNINNPKYLELAKLEHNKLHSMHTEEA
NSILLWWNSYGFDDPLITQLNPKEIHLSICAALCEPKFSTARITYTKWNCIESMLKYMFESHHSIEELKLFSHAINEWKPSLVQVLPTQL
KRVFMGTYDTMNGLAIEGKDVQGIDVFPYLHDLRKKQVQQYLMFREVKDKKQFKNFEDYVHQVKRGIAVAIRLVPAMFLMGDVA
PDKVLKLLDQKSTILDYLDTYIALMNLVTEERVAISLYMKEQNYSVQEAKAFLEEKIDETFANLTQEYLKPNNHISHFHRRLIFEHGRIF
RFFL
>Peq009409

RMTMDAYVMALYFRLSREHGFVASKDLLVQCYKDKNGSYKPSVLHDTKGILSLYEASFLAMDGEDELDELKEFTLKQLRYLKTSNSS
TNPKLSEYIAKALELPLHHRIPKLQAPIFIRLARDIEEVNPDPIVIEFAQLDFNMTQSIYIRELKEITSYV
>Peq011850
IGDDDDDLYAVALRFRLLRQHKYQITSDVFIKFLDDKGEFKSPLCNDVRALLSLYEATYLGFPDEEILDKAKNFSRVHLKSSIYNTEPSL
ALMVSSALELPLVRRTDRLKARSYLEIYENYNLCNKKLIDFSKLDFNLLQAIHKEEVQVISVW
>Peq017062
GDGELSTSPYDTAWVARIPSKNDPSKPHFPTTLEWVVKNQLEDGSWGEPIFFSLYDRLVCTLSCVITLTQWKQGHELIAKGLYFLQTRI
KDLDGEKGVRTVGFEIIFPSMLNEAKVIGLNLPYDLPCIRHIIKLREEKMTRIPMEVLHSVPTTLLYSLEAIEADLVQWDRILKLQCRNG
SISDSPSATAAAYLNTNDEKCLEYLTYIVKKFQDCAPSLYPVDTFERCWIIDTIQRLGIDHHFRKEIHITLEFVYRILRKDGLAWGSDASI
TDIDDTCMGLRLLRLHGYPISSDMLEYFKDDEGNFLCFMGETHKGISDFFSLYRFSQIAFPGENILKHAKKLAEQHLKSKMKGHQAFD
KWAIKKALHKEVEWAIRNQWRMSLPRLEAQEYIRNYGENDVWIGKSLYR
>Peq006301
NNLIENNNLYGLALLFRLLREHGITNTSSLRADVLITNIKRDGQSFNPNHQSDVEGMLSLYEASYLAIEGEEELYESGKLAKEQLRYIDS
YLISPQLAENINYALEIPLHWRMPTRWFIDSYGRRENVNPTLLELAKLDFNMVQSIYKAELQEMSRWWKNLGLVCDELNFARDRLVE
NYLWAIGIAYQPKFWRCREAITKIICLTTIIDDIYDVYGTLDELKLFTKAIEEWKMDAAHQLPDFMKICFNALLNTLNGIASSFSMEKQ
LDILPCLKRVLTDLCKAFLVEANWYHNRYTPTLDEYLENAWITIAGICPLIASYCLNDDMTIEDLNSLEFYQPIVRYSCLLLRLYDDLGT
TTAEIQRGDVPKSIQCYMNENNVLESVARDHIKCLITNYWKKLNNERAKISRSIESFKKALVDMPRTAQCFY
>Peq006309
NNLIENGNLHGSALLFRLLREYDIKNASLLRVEALINCFKSERGNFNPNHQHNVKGLLSFYEASYLAMEGEEQLDEAGKIAMERLRN
LDISLLSPQIIEEINHAFELPLHWRMSRLHARWFIDVYGTRENVNPTLLELATLDFNIVQSIYKAELQEMSRWWKNLGLVCDELNFAR
DRLVENYLMALGFTFQPEFWRIRKAITKLNSFVTTIDDIYDVYGTLDELKLFTNAVEEWKIDAGQQLPNYMKKCLDALFNTMNDIAT
SFSVEKELDILPCLKRVVIKYLFQFSMSFKL
>Peq013532
CEVVIPCGLRTTAICFRLLRRFGFQVSEGIFDDFMDSDHKTFMPRPCDDADELLSLYNASDMAFHDETKMDAARSFAVKHLKVISTVG
RIKRSVKHALDLPLQRRMPWMEARVYMDMYEDEDGMSPALLQLAKIHFNEVQSIHQQELKQVVNWWGRLDMGKTLSFSRNRLLE
CFFYVVGIVNDPDYGFCREKLTQVGLLIAYIDDVYDIYGSLEELELFTQIIDRDMKDMIELPDYMKICFSTLNDIINEVTSQLSMDDSHK

	HMQWADLFKTFLVEAKWRKDRYIPTLKEYLSIATISISGHVILLHTYVLLKCKITKETLQQLQDFPNIIRFSSLIFRLSNDLATSSNLQEER<br>ERGKTAIAVQCYMNEKGASEEEARLYISVLISDAWKELNREYSACSYYCQFPQQFIDASINLARMASFMY<br>>Peq013535<br>CEVVIPCGLRTTAICFQLLRRFGFLVSEGIFDDFMDSDHKTFMPRPCDDADELLSLYNASDMAFNGEKKMDAARSFAVKHLKLISTVG<br>RIERAVKHALDLSLHRRMPWMEARVYIDMYEDEDGMSPALLQLAKIHFNEVQCIHQQELKNVINWWARLDLGKTLSFSRNRLIECF<br>FYVVGIVHGPDSGFCREKLTQVGMLIATVDDVYDIYGSLEELELFTQIIDRWDMKDMIKLPDYMKICFSTLNDLITEVASQLSLNDRLE<br>VMPYLKRAEERDRGETAIAVQCYMNEEGASEEEARMYVSVLISDAWKEMNREYFTCSYYSKFPQQFIDASINLARMASFMY<br>>Peq025553<br>KDEEIYANIATCAMAFRILRMNGYDVSSNALAQFADVKQFKNTLQGHMRDLNAIIELYKASLVQISPEEPVLKKLNAWVTHFLKEEL<br>NTNAIHSLDVLQEVDYTLKFPFYASLERLEHKRNIEHYKWHEIHEKDFYSENVKIIFLALFNTVNELGAKASLFQNRDVTKHIIKMWQ<br>DLLKSTMKEANWIENSTVPTIDEYMTNAIISFALEPIILPTIYFIVPELSENIIGSSEYYNLFKLVSKFGRLFNDIHGFETDIAAGKRNSLWL<br>LVHHGNGSISEEDVKKDMLSLIDSTRAELLGLVLQNVGSVVPRACKEVFWKTCKTLQIFY<br>>Peq011573<br>NKNNVAFQLYKDSLAFRLLRMKGYQVSPWRFCWFIDDEEIMSHIKDNHRFFLGPMLSIYKASHIAFPDDHKLDKAGEFARQILQMGI<br>LSMKSKNETIFLSTSPLLQQEIEHELGLKWLARMDHLEHRFYIERGGIYLFWIGNNAPYCKILQNDDLLQLAIDNFTTRQIVYKKELHE<br>LHRWAKDTGLSTMGFGREKTSYCYFAVASSICFPINIDSRKEAAKCATFIAVADDFFDEKAPLDELFILTDALQRWDGEFLTGHSKVIF<br>NALDNLVHDISQKSFKRYGYDVKKILQDAWKEIFNSWLKEAEWARSSHCPSIDQYIEIGKTSVAVQMMIFASCYLTNSNIYVEESCWD<br>ISNTMITQSVMESCRLLNDLESYEKEAMVGKPNIILLYLKENPKEGIDDAKAFVMNILEKKKKKILQLVMSKDSTKSEMPTEWKDLHL<br>SALKVFQMFF
Populus trichocarpa	>Pt0001s31550<br>DNREMKEDLYGTSVEFRLLRQHGYNVPQVFNSFKDEQGNFKNCLRDDVKGMLNLYEASYYLVNGESILEEARDFSEKHLKEYSKEQ<br>NEDHYLSLLVNHSLELPLHWRMQRMEARWFIDAYGRKRDLNPILLEFAGLDFNMVQAKYQEDIRHASRWWTSMDLGNKLFYTRD<br>RLMENTLWAVGEVFEPQFGYYRKMATRITALITALDDAYDVYGTLEELEVFTDVIESWDVNALDQLPYYMKISFFALFQSINEIGYNIL<br>KEQGINVVPSLKKLWGDLCRAFLKEAKWYYAGYTPTLQEYLDNAWLSVSGQVILGHAFFLVTNQLTEEAVRCCMEYPDLIRHSSTIV<br>RLADDLGTSSDEIARGDNPKSIQCYMHETGATEQEAREHVRYLIYETWKKLNVEILPFSKKFMGIPMDLARTAQCFY<br>>Pt0001s31570

DNREMKEDLYATSVEFRLLRQHGYNVPQDVFNSFKDEQGNFKNCLRDDVKGMLNLYEASYYLVNGESILEEARDFSEKHLKEYSKE
QNEDHYLSLLVNHSLELPLHWRMQRMEARWFIDAYGRKRDLNPILLEFAGLDFNMVQAKYQEDIRHASRWWTSMDLGNKLFYTR
DRLMENTLWAVGEVFEPQFGYYRKMATRITALITALDDAYDVYGTLEELEVFTDVIESWDINALDELPYYMKISFFALFQSINEIGYNIL
KEQGINVVPSLKKLWGDLCRAFLKEAKWYYAGYTPTLQEYLDNAWLSVSGQVIIGHAFFLVTNQLTDEAVRCCMEYPDLIRHSSTIV
RLADDLGTSSDEIARGDNPKSIQCYMHETGATEQEAREHVRYLIHETWKKLNAEILPFSKKFMGIPMDLARTAQCFY
>Pt0001s31580
DNREMKEDLYGTSVEFRLLRQHGYNVPQDVFNSFKDEQGNFKNCLRDDVKGMLNLYEASYYLVNGESILEEARDFSEKHLKEYSKE
QNEDHYLSLLVNHSLELPLHWRMQRMEARWFIDAYGRKRDLNPILLEFAGLDFNMVQAKYQEDIRHASRWWTSMDLGNKLFYTR
DRLMENTLWTVGEVFEPQFGYYRKMATRVNALITTLDDAYDVYGTLEELEVFTDVIESWDINALDQLPYYMKISFFALFQSINEIGYNI
LKEQGINVVPSLKKLWGDLCRAFLKEAKWYYAGYTPTLQEYLDNAWLSISGQVILGHAFFLVTNQLTEEAVRCCMEYPDLIRYSSTIV
RLADDLGTSSDEIARGDNPKSIQCYMHETGATEQEAREHVRYLIHETWKKLNAEILPFSKKFMGIPMDLARTAQSFY
>Pt0001s44080
DDDHHHNDLYAISLKFRLLRQQGYKISCDVFGKFKNSQGNFNDSLVNDTRAILSFYEATHLRVHGDEVLEEALVFTTSHLEFLATHSS
SPLRAKINHALKQPIRKNIPRLEARNYFSVYQEDPSCSEVLLNFAKLDFNILQKQHQKELSDIAKWWKELDFAKKLPFARDRVIECYF
WILGVYFEPEHFLARRMLTKVIAMTSVIDDIYDVYGKPEELELFTDAIERWEITAVDLLPEYMKVTYKALLDVYTEIEENMVNEERSYR
VYYAKEAMKNQVRAYYHESKWFHQKHTPTMEEYMAVALVTSAYAMLAATSFVGMGVVTKDSFDWLFRGPKILKASEIICRLMDDI
VSHKFEQKRGHVASSIECYMKQHGTTEQETVHEFRKQVTDAWKDVNEEFLAVPMPLLTRMLNLARVIDVVY
>Pt0002s05300
ARNSEVHDIDDTAMGFRVLRLNGHHVSADVFKHFEKGGEFFCFAGQSTAAVTGMFNLYRASQLLFPGEKILEKAKEFSFKFLREKQA
ANLLDKWLITKDLPGEVGFALEIPWHASLPRVESRFYIEQYGGEDDVWIGKTLYRMPYVNNNEYLQLARLDYNNCQALHRIEWANF
QKWYEECNLRDFGISRKTLLYSYFLAAASVFEPERSNERLAWAKTTILLEMIHSYFDDDNSGAQRRTFVHEFSTGISRSGKTRKELVKM
LLGTLNQLSFGALEVHGRDISHSLRHAWERWLISWELEGDRRRGEAELLVQTIHLTAGYKVSEELLVYHPQYEQLADLTNRICYQLG
HYKNKHDNGSYSTIGSTDRITTPQIESDMQELMQLVIQKGIDPKIKQTFLQVAKSFY
>Pt0004s02970
SPNHLPLDVFGVALRFRLLRQEGYNVSQEVFNNFKNEEGNFHLIQENDVKGLMALYEASQLSMESEDILDEAGEFSAKLLNHHESEI
VANTLKHPYHKSLARFMVKNFLNNIDIRNENIKVFSELAKIDCEIVRSIHQKEILQISNWWKDLGLAKELKFARDQPLKWHMWSMSV

LIDPNLSEQRVELTKPISLVYIIDDIFDLYGTLNDLSIFTEAVNEWDLTAANQLPESMKISLKALFDITESISTKILEKHGWNPIESLQKSW
KKLCNAFLEEAKWFASGKLPKPEEYLRNGIVSSGVHVVLVHMFFLLGQGINKETVDFVVGFPPIISFTATILRLWDDLGTAKDENQDG
HDGSYLECYIREHNVTVERAREHVSHLICDAWKKLNQECLPFSPSFTKACLNVARMIPLMY
>Pt0004s02990
SPNHLPLDVCGVALRFRLLRQEGYNVSQEVFNNFKNEEGNFHLIQENDVKGLMALYEASQLSMESEDILDEAGEFSAKLLNHHESEI
VANTLKHPYHKSLARFMVKNFLNNIDIRNENIKVFSELAKIDCEIVRSIHQKEILQISNWWKDLGLAKELKFARDQPLKWHMWSMSV
LIDPNLSEQRVELTKPISLVYIIDDIFDLYGTLNDLSIFTEAVNEWDLTAANQLPESMKISLKALFDITESISTKILERHGWNPIESLQKSW
KKLCNAFLEEAKWFASGKLPKPEEYLRNGIVSSGVHVVLVHMFFLLGQGINKETVDFVDGFPPIISFTATILRLWDDLGTAKDENQNG
HDGSYLECYIREHNVTVERAREHVSQLICDAWKKLNQECLPFSRSFTNACLNVARMIPLMY
>Pt0004s03810
WTNSMATQLYKDSLAFCLLRMHGFRVSPGMFCWFLLEEVQDQIESNHEYFSSVILNVYRATDLMFPGDHELEEARSFSRKLLEKTTS
MGNEDQHTVPFPSHSVIKHELRFPWMARLDHLEHRMWMEEEEHWNIVMMWTNIQRFDTCRLSCLHNDKLKQLAVKNYEFRQTT
YKSELEELTRWSKSWGLSDMGFGREKTAYCYFAVAASTSLPQDSEIRMMVAKSAIVITVADDFYDMEGSLDDLEKITDAVQRWDATL
SGHSKTIFDALDSLVNELARKYFRQHGTDITNSLRDIWSETFASWFTEAKWSKSGFIPAAEEYLETGMTSIASHTLVLPASCFLSPSIPDY
KLNPVQYESITKLLMVIPRLLNDIQSYKKEQKEGKTNFVLLHLKENEADIEDSIAYAREILDKKKKELLEHMDDFSKPCRHLHLSCVKV
FQMFF
>Pt0005s09830
GNNFDDDLFTVALRFRLLRQYGYNVSSDIFNEFKDGKGNFKDNLIDDVEGLLSLYEASFLGGHGEDTLDKALSFCKTHLESAVAHLV
SPLADKVSHALKRPLLKGVPKHEQWHHILIYQQDEACTGAVLKLAKLDFNVVQKCYQDELRIISRWWIDLDFATKLPFARDRVIECF
FWGLGAFLEPQFVLARRFITKVLIFLSILDDIYDVHGTIEELELFTEKIERWDTSMEDLPDYMKLFFEALIGFFDEIEQETGKEGRPYCVH
YSREMLKNQARAYLIEARWFNQDCVPQLEEYRRGGVYTSCYPMAAVAWLCGMATGSKEVFEWMLKNPKIVVASSDIGRLMDDITS
HEFEQERGHVASAVECCMKQYGVSKKEAYDMLNKMVESDWKDINEELLTVPRQVLILMLNLARIIDVVY
>Pt0005s23190
ARNSNVHDIDDTAMGFRILRLHGHQVSADVFKHFEKGGEFFCFAGQSTGAVTGMFNLYRASQVLFPGEKILEDAKEYSFEFLREKQA
ANLLDKWIITKDLPGEVGFALEIPWYASLPRVETRFFIEQYGGEDDVWIGKTLYRMSYINNSEYLQLAKLDYNNCQALHRIEWENFQK
WYEECNLRDFGISRRTLIFSYFLAAASIFEPERSKERLAWATTTVLLDIVGSYFNHNNSSEQRRAFIHEFSYGINGREDLCLVCQELVKLL

LGTLNQLSLGALVVHGRDISHSLRHAWEKWLLIWELEGDRRQGEAELLVQTINLTAGYLVSEELLAHHPQYEQLVDLTNRICYQLD
HYKKNKVHYNGSYSTITSNTDRITTPQIESDMQELVQLVVGIDSNIKQTFLQVAKSFYY
>Pt0007s02810
GDLHTTSLHFRLLRQHAFSVSTDVFGKFRSRDGKFKDSIRTDVAGLLSLYEASYLGVPGEDHVLEEAKNFSSKHLKSLLETIKDEFLAQ
VKQSLEVPRHWKMPRIEARDFIDIYSSDNTRNLDLLELAKLDYNLVQSQHQRELKELARWWGALGFKEKLSFSRDRLMENYLWAM
GMVFEPQFSKCRIGLTKFVCILTTIDDIYDVYGLPEELELFTKLVNRWDSMAIDDLPDYMKICYLALFNFVNEMAYDVMRDHGLFVLP
YLVEEWANLCGSYLVEARWFSNKYSPTLSEYLENARTSIGSPAALAHACMLLGSVAQSSLMDCFKHGNDQLIYWSSLITRLSDDLGTY
TAESERGDVTKSIQCYMIEKGASEKESKEHIKGLINQAWKELNKEKCSLPKPLVNMSLNMARTAQCIF
>Pt0007s02920
SYVEAEKSLRATALCFRLLRQHDIFHGFIDDQGNFMASLHNDIEGMLSLYEASHLACEGEEILNKANKQTSIYLRNHLGNSDSITARVS
HALEVPLHHKMIMLEARWHIESYGKREDANPTLLQLSKFDFNMMQSVLQRDLQDMSRWWHDLGLKNKLRFSRDRLMECFFWTVG
MAFEPEFNSCRKGLTKVTSFITTIDDVYDVYGALDELEAFTEAVERWDVSAVRNLPDYMKLCFLALFNTVNEMAYDHLKEQGEDAIP
CLTKAWADLCKAFLQEAKWSYNNITPSFEEYLENAWRSVSGTVILIHAYFLMGENISKQALDYLVNYDELLRWPSIIFRLSNDLATSSA
EIARGETANSISCYMYETGASEAEARKHIEKLIQKAWRNMNKCQIPFARSFVGTINLARIAQCTY
>Pt0007s07360
FLENNDHDLHTVALLFRVLRQYGCKVSSDVFKKFKDTNGEFKKTITSDVKGNLSLHEAAHLSVNGEQILDEALEFSRTNLESLATQSG
PRLARHIKYALIRPIHKTVQRLEAREYISFYEEEDFRNETLLKFAKLDFNRVQLLHQQELSTLSSWWKDLNLVEELPYARDRIVEMYFW
VNAMHFEPQYALARILSTKLGALITVIDDTYDAYSTYEELQHFTKAVIRCNIDAIDQLPDSMKALYRALLSYFDDVANEVSKNGKSFT
AVNYVKEEMKEMIRTYIVEAQWCNDRFVPPLNEYVRNGKISIGFMATTTVFFVVETARIKELEWLTSKAKISEAGCLFLRLMNDIVTH
EFEQKREHCASAIECYMKEYGVSMNEAVKELQKTCADAWKDINEDCLAISMNLLNVCVNNARATDVVY
>Pt0007s07410
LLENNDHDLHTVALLFRVLRQNGCKVSSDVFKKFKDTNGEFKKTITSDVKGNLSLHEAAHLSVNGEQILDEALEFSRTNLESLATQS
GPRLARHIKYALIRPIHKTVQRLEAREYISFYEEEDFRNETLLKFAKLDFNRVQLLHQQELSTLSSWWKDLNLVEELPYARDRIVEMYF
WVNAMHFEPQYALARILSTKLGALITVIDDTYDAYSTYEELQHFTKAVIRCNIDAIDQLPDSMKALYRALLSYFDDVANEVSKNGKSF
TAVKYVKEEMKEMIRTYIVEAQWCNDRFVPPLNEYVRNGKISIGFMATTTVFFVVETARIKELEWLTSKAKISEAGCLFLRLMNDIVT
HEFEQKREHCASAIECYMKEYGVSMNEAVKELQKTCADAWKDINEDCLAISMNLLKVCVNNARATDVVY

>Pt0008s08190
GEEEIFSDNATCALAFRILRLNGYDVSLDTLNQFSEDHFSNSLGGYLKDSGAALELYFPPLQVHDALNFSDHANLQRLAIRRRIKHYA
TDDTRILKTSYRCSTIGNQDFLKLAVEDFNICQSIQREEFKHIERWVVERRLDKLKFARQKEAYCYFSAAATLFAPELSDARMSWAKN
GVLTTVVDDFFDVGGSEEELVNLIELIERWDVNGSADCSEEVEIIYSAIHSTISEIGDKSFGWQGRDVKSQVIKIWLDLLKSMLTEAQWS
SNKSVPTLDEYMTTAHVSFALGPIVLPALYFVGPKLSEEVAGHPELLNLYKVTSTCGRLLNDWRSFKRESEEGKLNAVSLYMIHSGSTE
EEAIEHFKGLIDSQRRQLLQLVLIIPRPCKDLFWNMIKLLHTFY
>Pt0008s08220
GEEEIFSDNATCALAFRILRLNGYDVSLEDHFSNSLGGYLKDSGAALELYRALQLSYPDESLLEKQNSRTSYFLKQGLSNVSLCGDRLR
KNIIGEVHDALNFPDHANLQRLAIRRRIKHYATDDTRILKTSYRCSTIGNQDFLKLAVEDFNICQSIQREEFKHIERWVVERRLDKLKF
ARQKEAYCYFSAAATLFAPELSDARMSWAKNGVLTTVVDDFFDVGGSEEELVNLIELIERWDVNGSADCSEEVEIIYSAIHSTISEIGDK
SFGWQGRDVKSHVIKIWLDLLKSMLTEAQWSSNKSVPTLDEYMTTAHVSFALGPIVLPALYFVGPKLSEEVAGHPELLNLYKVMSTC
GRLLNDWRSFKRESEEGKLNAISLYMIHSGSTEEETIEHFKGLIDSQRRQLLQLVLIIPRPCKDLFWNMIKLLHTFY
>Pt0011s03440
KNLKVEENLYVTALRFKLLRLHGYEVSQGVFNGFFDGTFDKSKCTDVRGLIELFEASHLAYEGEATLDDAKAFSTRILTGINCSAIESD
LAKHVVHVLELPSHWRVMWFDVKWHINAYENDKQTNRHLLALAKVNFNMVQATLQKDLRDVSRWWRNLGIIENLSFTRDRLVE
SFLCTVGLVFEPKYSSFRKWLTKVIIMILIIDDVYDVYGSLHELQQFTKAVSRWDTGEVQELPECMKICFQTLYDITNEMALEMQREKD
GSQALPHLKKVWADFCKAMFMEAKWFNEGYTPSLQEYLSNAWVSSSGTVISVHSFFSVMTELETGEISNFLEKNQDLVYNISLIIRLC
NDLGTSVAEQERGDAASSVVCYMREVNVSEEVARNHINNIVKKTWKKINGHCFAKSPTLQLLNTNMARVVHNLY
>Pt0011s14600
GAYVHDHDLQMVALRFRLLRQQGRYVSCDVFKKFKDTEGNYKVCLANDIQGMLSLYEAIHLRVHREDILEDALTFATTHLKSITTD
MCPPPLLVKLRHALDQPIHKDLPWLGAKHYISIYEQEASHSEVLLKFAKLNFNFLQNMHQKELADMTMWWKKVDLSKKLPFARDR
LVECYFWILGVCFEPQYSFARIIMTKVIAMTSVMDDVYDVYGTMEELVLFTDAIERWDISNIDHLPEYMKFFYKQLSDVYKEIETELAA
QGRSYRVDYAKEAMKKQVQAYFVEARWLHENYMPTMDEYMRISLISSGYPLLTCISFVGMGDIVTKDAFEWLNKDPKIVKAASLIAR
LMDDIVSHKFEQERGHVASAVECYMNQHEVSEEQAYDELRRQVVEAWKDINEELLHVPIPLLTRVLNLARVMDVMY
>Pt0015s05270
DMEDDDLYNTALGFRLLRQHGYNVSCDIFNKFKDDKGYFKPSNDVRGILGLYEAAHLAVHGEDILDEALAFTTIHLKSMATSPNCPL

TAKVSHALKQPIRRGVPRLESRRYISFYQDEPSCNKTLLRLAKLNFNVVQELHKEELSEITRWWKGLDFARRLPFARDRVVECFFWIVG
AYFEPQYSLARKILTKVIAMTSIIDDIYDVYGTLEELELFTEAIDRWDTKSMDQLPDYMKICYEALLNVYSEIEEKVAKEGWSYRVHFGK
QAMKVLVHAYFDEAKWFHENHIPTMEEYMQVALVTSGYSLLATVSFIGMGDMVTEQAFDWVFNRPKIVRASETISRLVDDVRSHKF
EQERGHAASGVECYIRQYGLSEQEVYKEFHMQVVNAWKDINEECLAVPMPLLERILNLTRVIDVIY
>Pt0015s09710
DMEDEDLYNTALGFRLLRQHGYNVSCDIFNKFKDDKGYFKQSNDVRGILGLYEAAHLAVHGEDILDEALAFTTIHLKSMETSPNCPL
TAKVSHALKQPIQRGVPRLESRRYISIYQDEPSCNKTLLRLAKLNFNLVQELHKEELAEITRWWKGLDFARRLPFARDRVVECFFWIV
GVYFEPQYSLARKILTKVIAMTSIIDDIYDVYGTLEELELFTEAIDRWDTKSMDQLPDYMKICYEALLNVFSEIEEKVAKEGWSYRVHYG
KDAMKVLVHAYFNEAKWFHENHIPTMEEYMQVALVTSGYSMLTTVSFIGMGDMVTKQAFDWVFNHPKIIRASETIGRLMDDVKSH
KFEQERGHAASGVECYIRQYGLSEQEVYKEFHMQVVNAWKDINEECLAAPMPLLERILNLSRVIDVIY
>Pt0017s06920
FDAVTKTSLHATALSFRLLRQHGFEVSQEAFGGFKDQNGNFMENLKEDIKAILSLYEASFLALEGENILDEAKVFAISHLKELSEEKIGK
DLAEQVNHALELPLHRRTQRLEAVLSIEAYRKKEDADQVLLELAILDYNMIQSVYQRDLRETSRWWRRVGLATKLHFARDRLIESFY
WAVGVAFEPQYSDCRNSVAKMFSFVTIIDDIYDVYGTLDELELFTNAVERWDVNAIDDLPDYMKLCFLALYNTINEIAYDNLKEKGE
NILPYLTKAWADLCNAFLQEAKWLYNKSTPTFDDYFGNAWKSSSGPLQLVFAYFAVVQNIKKEEIENLKKYHDIISRPSHIFRLCNDL
ASASAEIARGETANSVSCYMRTKGISEELATESVMNLIDETWKKMNKEKLLFAKPFVETAINLARQSHCTY
>Pt0019s01270
ENTETVHDLYATALEFRLLRQRGYHVPQEVFNHFKDEQGNFRACIHDDLKGMLNLYEASYFLVDGENILEDARDFTTKNLENYVKK
CNTTEYLSELVSHALELPLAWRMLRLEAHWFINLYETKTDMEPVLLELAKLDFNMVQAVYQEDLKDSSRWWKMTGRGEKLDFARD
RLVVYLLWSVGIIFEPQFGNIRRMITKLNSLITTIDDVYDVYGTLDELELFTDAVVRWDLNFMDHLPDYMKLCFFALFNSINEIAYDILR
DQGVDNLPYLKKTWADLCKSHLLEAKWYYSGYTPTLQEYLDNAWISVGAPLAIVHAYFYASNPTTKEASHFMEEYPDIIRWSSIILRL
ADDLGTSSDEMKRGDVSKSIQCYMYESEASEEEARDHIRKLISNAWKKINAYQFHISQTIIGVVVNLARAAQCIY
>Pt0019s01290
TVHDLYATALEFRLLRQRGYHVPQEVFNHFKDEQGNFRACIHDDLKGMINLYEPSYFLVEGENILEDARDFTTKNLENYVKKCNTTE
YLSELVSHALELPLAWRMLRLEAHWFINLYETKTDMEPVLLELAKLDFNMVQAVYQEDLKDSSRWWKMTGLGEKLDFVRDRPMV
WDVNFMDHLPDYMKLCFFVLFNLINEIAYDILRDQGVDSLPFLKKAWIYTNAWISIATSLAIVHTYFYASNPTTEEASHFMEEYPDIIR

WSSIILRLADDLGTSSDEIKRGDVSKSIQCYMHETQASEEEARDHIKKLISNAWKKLNASQFHISQTIIGVAVNLPRTAQCIY
>Pt0019s01320
LLEKHDFDLYTVSLLFRVLRQHGFKMPCVVFDKFKDTNGEFKKTIINDVKGILSLYEASLLSVHGEQVLDEALVFTKANLESLAMQSN
PRLADHIRNALIRPFHKGVPRIEARKYISFYEEEESRIDTLLKFAKIDFNRVQLIHRQELSILSRWWNDLNFSEEFPYARDRIVEIYFWAN
GIHFEPQYAFSRMMVTKYTKIVSLVDDTYDAYASFEEIQHFTNAIERCSMNAIDQLPDYMKVLYRALLNLFNETENDMGKQGRYYAS
YYVKEAFKELVRGYHAEAEWADKCHVPTFDEYVRNGLATSAYGVIMAASFLGMEEVAGGEEYEWLKSNPKIIKAGKMIGRLMNDI
VGHEDEQKRGDCASGVECYMKQYDVSEKKAIEEIQKMDVNAWKDINEDCMNAPMLLLQHFVNLIRVTDVIY
>Pt0019s01340
ENTKTVHDLYATALEFRLLRQHGYKVPQEVFNHFKDEQGNFRAWIHDDLKGMLFLYEASYFLVEGESILEDARDFTTKNLEKYFKKC
NPSEYLSKMVSHALELPLAWRMLRLESNWFINVYETKTDMEPVLLELAKLDFNMVQALHQEDLKHSSRWWKRTGLGEKLDFARDR
LVENFLWTVGVIFEPQFGNCRRMLTKVNSLITTIDDVYDVYGTLDELELFTDAVVRWDLNFMDRLLDYMKLCFLAFYNSVNEMTYDI
LKYQGVDILPYLKKAWADLCKSYLLEAKWYFSGYTPTLQEYMENAWISISAPVILVHAYFYVSNPTTEEASQFMEEYPDIIRWSSMILR
LADDLGTSTDELKRGDISKSIQCYMHEAGVSEEKAREHIRNLIENTWKKINDYQFRISQTFIGIAINLARMAQCMY
>Pt0019s03350
LLEKHDFDLYTLSLLFRVLRQHGFKMPCVVFDKFKDNNGEFKKTIINDVKGILSLYEASFLSVHGEQVLDEALVLTKTNLESLAMQSSP
RLAHHIRYALIRPFHKGVPRIEARKYISFYEEEESRNDTLLKFAKIDFNRVQLLHRQELSILSRWWNDLNFSEEFPYARDRIVEIYFWAN
AIHFEPQYAFSRMMVTKYTKIVSLLDDTYDAYASFEEIQHFSDAIERCCMDAIDQLPEYLKVLYRALLNLFNETESDMGKQGRSYASY
YVKEAFKELTRGYQVEAQWADVGHVPPFDEYVPNGLETTGYGVIMAASFVEMDEVAGEEEYKWLKSNPQIMKAAKMIGRLMNDIV
GHEDEQKRGDCASGVECYMKQYDVSDKKAIEEIQKMVANGWKDINEDCMNAPMLLLQHIVNLARVTDVVY
>Pt0019s03980
ENTETVHDLYATALEFRLSRQRGYHVPQEVFNHFKDEQGNFRAWIHDDLKGMLNLYEASYFLVEGENILEDARDFTTKNLENYVKK
CNTTEYLSELASHALELPLAWRMLRLEAHWFINLYETKTDMEPVLLELAKLDFNMVQAVLQEDLKDSSRWWKMTGLGEKLDFARD
RLMENFLWSAGIIFEPQFGNCRRMLTKLNSLVTTVDDIYDVHGTLDELELFTDAIVRWDLNFMDVLPDYMKLCFFALFNSINEVAYDI
LRDQGVDSLPYLKKAWADLCKSYLLEAKWYYSGYTPTLQEYLDNAWISIGVPLAIVHAYFYAPNPTAEEASHFTEEYPDIIRWSSMIV
RLADDLGTSSDEIKRGDVSKSIQCYMHETEASEEEARDHIKKLISYAWKKLNASQFHISQTIIGVAVNLPRTAQCIY
>Pt0019s03990

LLEKHDFDLYTVSLLFRVLRQHGFKMPCVVFDKFKDTNGEFKKTIINDVKGILSLYEASFLSVHGEQVLDEALVFTKANLESLAMQSN
PRLADHIRNALIRPFHKGVPRIEARKYISFYEEDESRIDTLLKFAKIDFNRVQLIHRQELSILSRWWNDLNFSEEFPYARDRIVEIYFWAN
GIHFEPQYASSRMMVTKYTKIVSLMFRKGNGIFCLNFRCSMNAIDHLPDYMKVLYRALLNLFNETENDMGKQGRSYASYYVKEAFK
ELVRGYHAEAEWADKCHVPTFDEYVRNGLATSAYGVIMAASFLGMEEVAGGEEYEWLKSNPKIIKAGKMIGRLMNDIVGHEDEQK
RGDCASGVECYMKQYDVSEKKAIEEIQKMDVNAWKDINEDCMNAPMLLLQHFVNLIRVTDVVY
>Pt0019s06190
LLEKHDFDLYTLSLLFRVLRQHGFKMPCVVFDKFKDTNGEFKKTIINDVKGILSLYEASFLSVHGEQILDDALVFTKANLESSAMQSSP
RLADHIRNALIRPFHKGIPRIEARKYISFYEEDESHMDTLLKFAKIDFNRVQLLHRQELSILSRWWNDLNFAEEFPYARDRIVEIYFWVN
AIHFEPQYAFSRMVVTKYTKFVSLLDDTYDAYASFEEIQHFTNAIERCCMDAIDQLPEYLKVLYRALLNLFSETESDMGKQGRSYASYY
LKEAFKELARAYQVEAQWADEGHVPTFDEYVRNGLATSSYGVTTAASFVEMDEVAGREEYEWLNSNPKIIKAGKMIGRLMNDIAG
HEDEQKRGDCASGVECYMKQYDASEKKAIEEIQNMVANGWKDINEDCMNAPMLLLQHIVNLVRVTDVMY
>Pt0019s06220
LLEKHDFDLYTLSLLFRVLRQHGFKMPCVVFDKFKDTNGEFKKTIINDVKGILSLYEASFLSVHGEQILDDALVFTKANLESSAMQSSP
RLADHIRNALIRPFHKGVPRIEARKYISFYEEEESRNDTLLKFAKIDFNRVQLIHRQELSILSRWWNDLNFAEEFPYARDRIVEIYFWAN
GVHFEPQYAFSRMVVTKYTKIVSLLDDTCDAYASFEEIQHFTNAIERCCMDAIDQLPEYLKVLYRALLNLFSETESDMGKQGRSYALY
YVKEAFKELARAYRVEAQRADEGHVPTFDEYVRNGLTTSAYGVITAVSFVGMDEVAGQEEYKWLKSNPKIMKAGKMICRLVNDIVG
HEDEQKRGDCASGVECFMKQYDVSEKKAIEEIQKMVANGWKDINEDCMNAPMLLLQHIVNLVRVTEVTY
>Pt0092s00200
DNREMKQDLYATSVEFRLLRQHGYNVPQDVFNSFKDEQGNFKNCLRDDVKGMLNLYEASYYLVNGESILEEARDFSEKHLKEYSKE
QNEDHYLSLLVNHSLELPLHWRMQRMEARWFIDAYGRKRDLNPILLEFAGLDFNMVQAKYQEDIRHASRWWTSMDLGNKLFYTR
DRLMENTLWTVGEEFEPQFGYYRKMATRVNALITTLDDAYDVYGTLEELEVFTDVIESWDINALDQLPYYMKISFFALFQSINEIGYNI
LKEQGINVVPSMKKLWGDLCRAFLKEAKWYYAGYTPTLQEYLDNAWLSISGQVILGHAFFLVTNQLTEEAVRCCMEYPDLIRHSSTI
VRLADDLGTSSDEIARGDNPKSIQCYMHETGATEQEAREHVRYLIHETWKKLNAEILPFSKKFMGIPMDLARTAQSFY
>Pt0121s00250
FDLSTVSLLFRVFRQHGFKMPCVVFDKFKDTNGEFKKTIINDVKGILSLYEASFLSVHGEQVLDEALVFTKANLESLAMQSNPRIADHI
RNALIRPFHKGVPRIEARKYISFYEEDESRNATLLKFAKIDFNRVQLIHRQELSILSRWWNDLNFSEEFPYARDRIVEIYFWANGIHFEPQ

	YAFSRMMVTKYTKIVSLVDDTYDAYASSEEIQHFTNAIERCSMNAIDQLPDYMKFKELVRGYHAEEEWADKCHVPTFDEYVRNGLA<br>TSAYGVIMAASFLGMEEVAGGEEYEWLKSNPKIIKAGKMIGRLMNDIVGHEDEQKRGDCASGAECYMKQYDVSEKKAIEEIQKMDV<br>NAWKDINEDCMNAPMLLLQHFVNLIRVTDVIY
Sorghum bicolor	>Sb01g015070<br>FSTLHEAALRFRLLRTHGLWVSPDELSKFRGDDGSFRTEIMNDHRCLLSLYNAAHLLTHGEVELEEAILFARQHLELGLLTSSLRAPLA<br>GQVTRALKLPLPRTLKRLEALDYMSEYTQEQTYNPSILELAKLDFNLLQRLHLKELKAICQWWKDIYQEVELNYMRDRVVECFFWSY<br>TVYYEQDHLRARAMLTKIFALLTVVDDTFDDHATLDESRKLAEALRRWDNSAVSMLPEYLRKFYLRLLQNFEDFEDELPPNERYRVA<br>YTREAFQMTSESYLQESEWFHHSCKPKFQEQVNVSTVSIGPQIAATAMLMGMGDEATRDAFEWALRGDTAVMSFGWIARFLNDIAS<br>FNSGKSKKDVATSVECYMNEYNVTSEVAMTEIGYLIEDGWKTANRARFELLPAVQRIINLTVCMPFTY<br>>Sb01g021990<br>ARNSNVKDVDDTAMAFRLLRLHGYNVSPSVFKNFEKDGEFFCFVGQSTQAVTGMYNLNRASQIAFQGEDVLHRARIFSYEFLRQRE<br>AQGLRDKWIIAKDLAGEVQYTLDFPWYASLPRVEARTYLDQYGGKDDVWIGKTLYRWYIENCLDTFGVQPQDVLRAYFLAASCIYE<br>PSRAAERLAWARTSMIANAISTHLDISADKKRLECFVHCLYEESDVSWLKINPNDAILERALRRLINLLTQEALPIHEGQRFIHSLLSLA<br>VIEICAGRISEAISVINNKDSDWFIQLTCNTCDGLNHKVLLSQDAEKNEATINCIDKIELNMQELQLLRSDEKTTNKKTKQTLWDVLRS<br>SYYHCPQHIIDRHVSRVI<br>>Sb01g032610<br>RNESFHEISLQFWLLRQDRYYVSCDVFQSFMDNQQNLNVSLQSDVRALLALYEAAHLGTPNEQFLIEAQRQTTSLLRSMVDHLEKPL<br>ADKVRHALQTPSFRRMKRLEARLYIPLYEEDKEDCDELILELAKLDFYLLQQIHREEVKEICEWYHGLDSPRKLFYARHRPAEAYFWA<br>LGVYYEPQYAKARKLLAKFIATITPYDDTFDNYGMWEELQPFADVMQRWDMKEAEGLNECYSDFARFMFGTMIEIENALPKDIGRR<br>NVNFIRDIINEVCKGYVTEIGWRDSKYIPLLEEHLKVTLITCFYWAINCTAFVVFEENVTEEILKWMSKFPQIVKDSCIISRLMDDIVAHE<br>FETERNNVATAVTCYMNEYKTTKEEASDVLWNSVEHAWKSMNHEYLSLPSSLLIRVINLARMMETMY<br>>Sb01g034700<br>GGGAADDGLHTVALRFRLLRQHGVWVPADVFDRFKDTTGGFSESLSSDPRGLLSLYNAAHMATPGEQGLDEAISFARRHLESLKLK<br>GTLSSPLAEQVCRALDIPLARLPKRLETMHYVVEYEKEEGHDAVLLELARLDFDLVRFLHLRELKDLSLWWKDLYGNVKLNYARDRL<br>VENYFWTCGVFHEEEYSRARMLFAKTFGLLSLMDDTYDVYATLEECHILNDAIQRWDENSASILPEYMRMFYINLVRNFQGFEDSLQ<br>PNEKYRVSYAKQAFKLSSKYYLDEAKWCSEKYAPSFKEHMEVSVMSSGFPTLAVVLLMGAGDMATREAFRWAIGVPAVVSASGEVA

RFRNDIASYKKKNKKDVASSVECYAKEHGTSGEEAAVAIAGMAEHAWRTINRSCMLLPAAQLVVNLTKTLEVIY
>Sb01g035460
NSTCLHDVALRFRLLRQHGFWISPDEFNRFKDKNGNFDVDITNDARGLLSLYNAAYLFTHGEAELEEAILFARQHLESMRNNLEYPL
AQLVNRALHLPLPRTFRRVEALHYISEYKGTPTHNPSLLEFAQLDFDLLQRLHLKELKALSRWWKDLYNEGELTYSRDRVVECYLWS
YTAYFEKEHTRARMILAKIIALIILTDDTYDVRATLEECRKFNEAIQRWEESAISLLPDYLKKFYLKLMNIFKEFEDELEPHEKYRVAFSR
KAFQILSSNYLQEAEWFHGGYKPTFKDQVKISTVCSGAPFASVGLLVGMGDVATKEALEWAISCTDAVKAFAEVTRFMNDLASFKR
GKNKNDVDSSVECYISEHGVTTEVAFAKINSLIEEAWKTINRARFELLPAVQRVANITASMPLMY
>Sb01g039090
DVDRVDDVRLMTLSFRLLRQNNYPVSPEVVRSLKDGTGNFKKTLQKDTEGLLSLYEASHLAFEGDHLLDEARVFSTEALRELRPSMH
PHLRSSVDNALAVPLHWAAPRLQARWFINHYARDSDADLSLLHFAKLDFNNVQKLQQQELSRITRWWRNADLSESLPFARDRLME
CFYFATGVAPEPSLEACREVVAKTFALIVLLDDIYDIYGTLDELVMFTDAIERWGTSASEQLPEYMKAIYLTIVSTSNEVAEHVLRQEGC
DARFLLKKAWHDLCKAFLMEAKWHYSNYKPTLHEYLENGWISVSGPLMLIHAFPIIEKGVTPNSIQQLESYPKLVQMVSKIFRLCNDS
ATHSEELKRGDAPSSIAIYMSENRATEHDARKAMRDLTMETWKTVNQDAFRFPLPFANACVNMARISHCIY
>Sb04g001780
HSDDLFDATLAFRLLREAGHDVSADVLRRFTDDSGEFKLPLSMDIRGLLSLHDMSHLDIGGEVLLYKAKEFSSKHLASAIRYLEPSLAE
YVRQSLDHPYHLSLMQYKARHHLTYLQSMPIRDTVVEKLAVAEFQLNKLLHQQEIQEVKRWWMDLGLVQEIPVVRDQVMKWYM
WSMTAVQGCSFSRYRVEITKIIALVYVVDDIFDLVGTLEELSLFTEAVKVWNMAAADSLPRCMRSCYMALYTVTNEITDMVEKEHGL
NHVNHLRKAWAVLFDGFMVEAKWLATEQVPTAEDYLRNGVVTSGVPLTFVHIFIMLGDQSIEALIDQMPSVISCPAKILRLWDDMG
SAKDEAQEGLDGSYMDFYLMENRCGPSDVEAHMRNLIAREWEELNRECLTFSSNLTQTCLNAARMISVMY
>Sb04g001800
HSDDLFDATLAFRLLREAGHDVSANDVQRFTKDSCEFKLPLSKDIRGLLSLHDMSHLNIGGEALLYKAKEFSSKHLASAIRYLEPSLAE
YVRQSLDHPYHLSLMQYKARHHLTYLQSLPIRDTAVEKLAVTEFQLNKLMHQKEIQEIKRWWMDLGLVQEIPIVRDQVLKWYMWS
MTTLQGYSFSRYRVEITKIIALIYVVDDIFDLVGTLEELSHFTEAVKVWNTAAADSLPSYMRSCYKALYTITNEIADMAKQEHGLNPVN
HLRKAWVVLFDAFMVEAKWLTMDQVPTAEDYLRNGVVTSGLPLTLVHIFIMLGDQSTETLIDHMPSVISCPAKILRLWDDMGSAKD
EAQEGIDGSYRDFYLMENRCGPSDAEAHMRSLIAREWEELNRECLTFSSNFTHACLNITRMISVMY
>Sb04g001810

SNDLFDATLAFRLLREAGYDVSADVLWRFTDNSGEFKLPLSKDIRGLLSLHDMSHMNIGGEALLDKAKEFSSKHLASAIRYLEPSLAE
YVRQSLDHPYHLSVMPYKARHHLTYLQSLPTRDTAVEKLAIAEFQLNKLLHQKEMQEIKRWWMDLGLAQEIPVVRDQVLKWYMW
SMTALQGYSFSRYRVEMTKIISLVYVVDDIFDLVGTLEELSLFTEAVKMWNTAAADSLPSYMRSCYKALYTITNEIADMAEKEHGLNP
ANNLRKAWTVLFDGFMVEAKWLANHQVPTAEDYLRNGVVTSGVPLTFVHIFIMLGDQNSEALIDHMPSVISCPAKILRLWDDMGS
AEDESQEGFDGSYKNFYLMENRCSPTDAEAHMRSLIAREWEELNRECLTFSSNFTQVCLNIARMVSVMY
>Sb05g006470
GGDDLFALALQFRLLRQHHYNVASEIFNNFMDENGDFKDALRSNVDGLLSLYEAAHLGKSDEDLLRKAIIFTKDCLSSLVNGGQLPK
PVLQEVLHALDLPTQRRIKRLEAKLYISIYENGDESNQDIVELAKLNFHMLQQMHRDEVRTISLWWYNDLNPSSLGPYMRKRPVECY
YWALGIFYEPQYAKARIVLTKLLTLLTMFDDIIDSYGTMEEVHLFNQAVQSWNEEAAKQIGDGYWYLIFHISKTLEEFVSKDGGSPMA
IDCFKETLKAGSKAMVQELVWREEGQVPTVHEYLKQGAAVSILYWPIAVISFAGMFPSDDEIFTWAGSYPKIIESSTTLCRLMDDVAG
HENEEERSKCVTAVECYVREHGVTVQEAKQALTCLVDEQWRCINQELYAVPIALLDPVLDLVRVMEEVY
>Sb05g019210
EILRSEELHIVALRFRLLRQHGFFVSTDVFDEFRDGTGNFNTCLTRDPKGLLSLYNAAYLAVPGEDVLDGVIAFTRTHLEAMKGNLTSP
IADQICRALDIPLPRYMPQLETMHFITEYEQEDGHNATLLELARLDYCLTRSAQLKELRTFCLWWKDFYKNVNLTYSLDRGVEMYFW
GFGVFPGEGNSRARIIFSKIVALISLMDDTFDTHATFEDCKNLDEAIQRWDESATSILPEYLRMYYTKMLSCFNEFEDILEPKEKYRVPY
VQKAVMLQSKYYLEEAKWCNEKYMPTFKDQIELSSLSSTIPVLTLAALMAAGNEATKEALEWASVVPDMKGRKNKNDVASSLDCY
MNEHGTTGTEAAAALSAMVEHAWRRINKAFMEIDRALLRAAVINQARTNEVVY
>Sb05g022320
ATDVLSLFAEESRFHDSVEGHMNDTKALLELYKASLVEYALNLPFYATLQPFKHKRNIECFGTEGIRIHKSAYLACDATENILALAIED
FHLSQSIYQQQLQYIERWVKEVRLDQLKFARDLPLSLFVFLATNVFPCELYDASIAWTQKCILTTVVDDFFEGGGSTKELRNFVTLIEK
WDMHAGIECSEDIEILFRAVYDTNNQIAAIGAKLQNRSVIDHIVEIWVKYVRTLMIEAEWTTKGHVPTMEEYMSVAETSSALGPVVVP
SLYLVGPKLSDDMIRDPEYKNLLRYLGIGIRLINDIGTYEKEMSEGYVNSVLLRAFDSSIEAAKREIHVLLANSQRELLKLVLPIPRPCKDI
FWNTYKIGRQFY
>Sb06g002820
KDHTYDNIAFTALKFRLLRENGFPEGQLGYHNYGNCTTKTPRQEDVNTLLLLHEASYLAFGDEEILDVARTYSAKALKELMPSMLPH
LREAVAHALEIPLHWRAPRLETRWFIDYYARDINMCPLLLQFAKLDFNQVQDEHQKDLAAVTWWWRNIGLGEKLPFARDRLMECF

HYANGIVWDPKLGPCRQMLAKVSNLIVHLDDVYDVYGTMDELVLFTNAIARWDAIPNERLPEYMKALYSVIYHTSNEVAEHALKE
HGCSMHYHLQKLWHDICMAFLLEAKWHHGNCRPSIQKYLENGWVSSSAPLLLSHAFSMLHSVINMNTISKMQTTHRLVQQVSLIFR
LCNDSATHMDELQRGDAPSFIAINMAENGNEDDSRKVMQDLILKSWKVINEEAFQYSTPFNKACVNLARISHCVY
>Sb06g028210
RDEEIMLDAATCAMAFRILRMNGYDVSSDELYHVAEASMFHNSLGGYLNDTRTMLELHKASTVSTSEDEYILDTIGSWSSTLLREQLG
SGGALRRTPLFREVEHALDCPFYTTLDRLDHRWNIENFNVTGHRMLETPYLSSRHTSRDILTLAVRDFSSSQFKYQQELKHLESWVKE
CKLDQLPFARQKLAYFYLSAAGTMFPPELSDARILWAKNGVLTTVVDDFFDVGGSKEELENLLLLVEMWDEHHKIEYSEQVEIVFSSI
YNSVNQLGAKASLLQDRNVTKHLVQIWLDLLKSMMTEVEWRMSKYVPTEEEYMANASLTFALGPIVLPTLYFLGPKIPKSAIKDPEY
NELFRLMSTCGRLLNDVETFEREYNEGKLNSVSLLVLHGSMSISDARRKLQKPIDTCRRDLLRLVLVIPRPCKELFWKMCKVCYFFY
>Sb06g028220
KDEEIMMDKATCAMAFRLLRMNGYDVSSDVLSHVAGPSTFHDSLQGYLNDTKSLLELYKASKVSLSENDLVLDGIGFWSGNLLKDK
LCSSRVKKDLIFEMEYAVKFPFYATLERLEHKRNFDAWGPLMLTTKSSSFCIDQEFVALAVEDFSFSQYVYQDELRHLDSWVKENKLD
QLQFARQKLTYCYLSAAATIFSSELSDARISWAENGVLTTVVDDFFDVGGSKEELENLIALVEKWHAHHTVGYSEQVKIVFSAIYTTV
NHLGVIASAAQGRDVTNHLVEIWLDLLRSMMVETEWQRSQYVPTVEEYMTNAVVSFALGPIVLPALYFVGQEVLEHAVEEYDELFR
LMSTCGRLLNDSQSFEREGNQGKLNSVSLLVRHSGMSIEAAKKALQKSIDVSRRDLLRLVLVVPRPCKELFWKMCKILHLFY
>Sb06g031270
LHDVALRFRLLRQQGFWVSPDEFNRFKDKHGNFDVGITNDARGLLSLYNAAHLFTHGEAELEEAILFARQHLESMRNNLEYPLAQQ
VNRALLVPLPRTVRRLEALHYISEYKESPAHDPSLLEFAQLDFDLLQRLHLKELKALSRYNQYLAIRIVLTFLGLNNVNTTHRYCSAAY
YEKEHSRARMILAKIIALIILIDDTYDVRATLEECRKFNEAIQRWEESAISLLPDYLKTFYLKLMNIFKEFEDELETHEKYRVAFSRKAFQI
LSSNYLQEAEWFHGGYKPTFEDQVKISTVCSGAPFASVGLLVGMGDVATEEALEWAISCTDAVKAFAEVTRFMNDLASFKHGKNKN
DVDSSVECYISEHGVTTEVAFAKINSLIEDAWKTINRARFLPVVQRVANITASMPLIY
>Sb07g003080
GASASSHDLYVTSLRFYLLRKHGYTVSSDVFAKFRDEQGNISSDDDMTTLMMLYDAAHMRTRGEDILDNIIVFNKSRLETVVKSENLE
PDLAEEVTITLETTRFRRAERVEARRFISVYEKKATRDDTILEFAKLDYNIVQAVYCDELKQLSMWWKDLRSQVDMTFSRDRLVEMYF
WMTVIVYEPDYSYSRIMLTKLVLYIALLDDIYDNYSTTDESNIFTTAFKRWDDKAVEEIPQHLRNFYKSVIRTADEIVAELKVQNNKNS
EVVREVMFHVAESYHAEVKWRDEQYVPADVDEHLQISLGSIMAMQVVVLTFVSMGDVTTREIIDWAFTYPRMIRAVTAMARILNDI

	MSYEREQASDHMASTVQTCMKQYGVTVEEAIEKLKFICEEAWMDIVQGCLYPMAILHKVVSVGRSLDFIY<br>>Sb07g004470<br>DVKDLNLVSLRFYLLRKNGYDVSSDVFLNFKDKDGNFASDDIRSLLSLYNAAYLRTHGEEVLDEAIIFTRRHLEAALTSLESKLADEVS<br>LSLQTPLFRRVRILETRNYIPIYEMEPSRNEAMLEFAKLNFNLLQILYCEELKTVTAWWKQLNIETDLSFIRDRIVEMHFWMAGACSEP<br>KYSLSRVILTKMTAFITILDDIIDTHSTTEEGKLLAKAIDRCSQDANEVLPDYMKHFYMFLLKTFDSCEDELGPNKRYRLKILVRGYSQEI<br>EWRDEHYVPETIDKHLEISRVTVGAFQLACSSFVGMGDIITKEVLDWLLTYPELLKCFTTFVRLSNDITSTEHSTTMHDACEKIKGLIED<br>SWKDMMQLYLEQSKVVAQTVVDFARTGDYMY<br>>Sb07g004480<br>DDKDLNLVSLRFYLLRKHGYYVSSDVFTSFKDKEGNFVADDTKCLLSLYNAAYLRTHGEKVLDEAIIFTRHQLEALLDSLESTLADEVS<br>VTLQTPLFRRVRILETRNYIPIYEKEAARNEVILEFAKLNFNLLQLIYCEELKKVTLWWKQLNVETNLSFIRDRIVECHFWMTGACFEPQ<br>YSLARVISTKMTACITILDDIMDTYSTTEEAMLLAEAIYRWEENAAELLPEYMKDFYLYLLKTIDSCDNELGPNKSFRTFYLKEVLKVLV<br>RGNSQEIKWRNENYVPETINEHLEHSGRSVGAFQVACSSFVGMGDNITKEILEWFLTYPELLKSFTTIARLSNDIASTKREQNVGHHVS<br>TVQCYMLKHGTTMDDAYEKIKELIEDTWKDMMELYLEQPKLVTQTVVDFARTADYMY<br>>Sb07g004485<br>DDKDLNLVSLRFYLLRKHGYDVSSDVFKCFQDKEGNFVVKDTKSLLSLYNAAHLRIHGEEVLDEAIIFTRGKLESVLDSLETTLADEVT<br>LALQTPLFRRVRILETRNYIPIYEKEVARNEVILEFAKLNFNLLQLLYCEELKMITLWWKQLNVETNLSFIRDRIVEMHFWMTGACSEK<br>KYSLTRTITTKMTAYITILDDIMDTHSTTEEAMLLAEAIYRCEENAAELLPEYMKDFYLYLLKTFDSVKHELGPNRSFRVFYLKELLKILV<br>RGYSQEIKWRDEHYVPETIDEHLEVSKATVGAFQVACSSFVGMGDIITKEILDWLLSYPKLLKSMTTFVRLSNDIASTKREQTGGHHAS<br>TVQCYMMQHGTTIHDACEKIKELTEDTWKDMMKLYLEQPKVIIQTVLDFARTAEFMY<br>>Sb07g005130<br>QDLDLPTTSHLFYLLRKHGYHISSDVFLKFRDDKGNIVTDDARCLLLMYEAAHLRVKGEEILDNILIFTKSQLQCIVDDLEPQLKEEVK<br>YALETPLFRRLKRVQTRQYISIYEKNTAHNNMLLEFSKLDFNILLTLYCEELKDLTLWWTEFQTQANTSIYARDRMVEMHFWMMGVF<br>FEPQYSYSRKMLTQLFMIVSILDDLYDNHCTTEEGNVFTAALERWDEEAVEQCPTYLRTLYVNILTTVKAIEEWLNLQNNKHAKLVK<br>RLIIDMAKCYNAETEWRDKKYVPATVDEHLKISARSSGCMHLVSQGFISMGDVATSEAIKWASTYPKIIQAVCIIARLANDIMSYKREE<br>TSQNMVSTVKTCAKEYGTTVAQAIEKLRELIEEAWMDITEECLQQPKVLLERVANLARTMDFLY<br>>Sb07g020980

	NSSSLHEVALRFRLLRQQGFWVSADEFEKFKNEDGSFISGITNDPKGLLSLYNAAHLLTHDEEILEDAILFSRQHLELIRSSLKSPLAEQV<br>GRALEIPLPRTLKREETISFIPEYSIQDQTYSPVILELAKLDFNLLQHLHQKELKEISQWWKELSGEIGLDYVRDRIVECYFWSYTVHYEQ<br>ENARARMILARLFLLTSLLDDTYDVHATLEEARELNKAIERWDDNDVSLLPEYLKEFFVKVISNFREFEDELESHEKYRNVYNIKGFQT<br>LSKYYLQEAEWFHHGYTPSFKDQVNVSVITAGGQVLSIGLLVGMGEATKEAFEWATGDTDAIWACGQVSRFMDDMSAFKNGRNN<br>MDVASSVECYMKERNVPSEVALATISSFVEDAWKTINQAKYPTLLPVVQRVTSLAKSMTLLF<br>>Sb07g025700<br>DNFHGSNDLHVVALRFGLLRQHGLWVSADVFDKFRDAMGSFSMDLATDSKGLLSLYNAAHMAVPGEAVLDDAVAFARRHLEAA<br>KGKLIRSPMVEQVSRALNTPRPRWPRRLEAMHYITEYEQEDEHNAIILELARLDFSIVRSVYIEEIKNLSLWWRDLYNDVKLPYARNRI<br>VETHLFSSGVFPEKEHSRARIIFTKTFAFLSLMDDTYDTHATLEECQKLTEAIQRHEYLRMFYIKLLRNYKEIEEDILEPWEKNRMADFK<br>KSFKLVSKSYLKEAEWFSQNYTPSFKEHIDFSITSTGLPMLSHVALMGAGQLATKEAFDWALDMPDLVKGMAETGRFFNDISSYKPR<br>NSLKDVVSSLECYMKEHDMTPNDATVAFETMVEHAWRRINKAYMELDHGILPAVVNMARTVQMFY<br>>Sb09g000990<br>SDYDLGTVALWFCLLRKHRYRVSSDVFVRFKDEKGGFLVDSPQDLLNLYNAAHMRTHGEVILEKAILFSQRRLETMIPYMEGSLLAEI<br>KSALEIPLPRRVRIYELKYYISTYEKDATVHEKVWQLAKLNSNIMQLHHQHELGIITSWDSKGAHDLPECMKFALEKIFDSYETIENML<br>HQEEKYRMAYLRYFVKDLVRSYSKEVKMLQEGYIPKSVEEHLKVSVITTTCPFLSCASFVGMHDIATKDFFDWVSSVPKMVQELSVILR<br>LVDDLGSYEREQLIPHVASTINSYMKEHNVSIEVARGQIQVLKEKSWKDFNSEWLAYPKQLLERIFNFTRTMEFIY
Solanum lycopersicum	>SlTPS10<br>KQNDNDNNLYVVSLRFRLVRQQGHYMSSDVFKQFINQDGKFKETLTNDVQGLLSLYEASHLRVRDEEILEEALTFTTTHLESIIVSNLS<br>NNNNSLKVEVSEALTQPIRKTLPRVGARKYISIYENNVAHNHVLLKFAKLDFNVLQKLHQRELNELTRWWKDLDFANKIPYARDRL<br>VECYFWILGVYFEPKYSRARKMMTKVLKITSVIDDTFDAYATYDELVAFTDAIQRWDASAIDSISPYMRPLYQALLDIYSEMEQVLSNE<br>GKLDRVYYGKHEIKKIVRAYFKEAQWLNDANYIPKYEEHMEISLVTAGYMMGATNCLVGVEEFISKDTFEWLKNEPLIVRAASLISRA<br>MDDIVGHEDEQKRGHVASIIECYMKEYGASKQEAYAKFKKEVTNVWKDINKEFFRPTEVPMFVLERALNFARVIDTLY<br>>SlTPS12<br>KQNDNDNNLYVVSLRFRLVRQQGHYMSSDVFKQFINQDGKFKETLTNDVQGLLSLYEASHLRVRDEEILEEALTFTTTHLESTVSNLS<br>NNNSLKAEVTEAFSQPIRMTLPRVGARKYISIYENNDAHNHLLLKFAKLDFNMLQKLHQRELSDLTRWWKDLDFANKYPYARDRL<br>VECYFWILGVYFEPKYSRARKMMTKVIQMASFFDDTFDAYATFDELEPFNNAIQRWDINAIDSVPPYLRHAYQALLDIYSEMEQALA

KEFKSDRVYYAKYEMKKLVRAYFKEAQWLNNDNHIPKYEEHMENAMVSAGYMMGATTCLVGVEEFISKETFEWMINEPLIVRASSL
IARAMDDIVGHEVEQQREHGASLIECYMKDYGVSKQEAYVKFQKEVTNGWMDINREFFCPDVEVPKFVLERVLNFTRVINTLY
>SlTPS14
NKNEDDNDLYIVALRFRLVRGQRHYMSSDVFKKFTNDDGKFKETLTKDVQGLLNLYEATHLRVHGEQILEEALSFTVTHLKSMSPKL
DSSLKAQVSEALIQPIYTNVPRVVAPKYIRIYENIESHDDLLLKFVKLDFHILQKMHQRELSELTRWWKDLDHSNKYPYARDKLVECYF
WATGVYFGPQYKRARRMITKLIVIITITDDLYDAYATYDELVPYTNAVERCEISAMDSISPYMRPLYQVFLDYFDEMEEELTKDGKAHY
VYYAKVEMNKLIKSYLKEAEWLKNDIIPKCEEYKRNATITVANQMILITCLIVAGEFISKETFEWMINESLIAPASSLINRLKDDIIGHEH
EQQREHGASFVECYVKEYRASKQEAYVEARRQIANAWKDINTDYLHATQVPTFVLQPALNLSRLVDILQ
>SlTPS16
YWIDDLHAIALCFRLLRQQGYRVSCDAYKKFTDDQGNFKIELINDVHGMLSLYEAAQFRVHGEEILDEALNFTTTQLKLILPKLSNSP
LAQQVANALKFPIKDGIVRVEARKYISFYQQNQNHNQLLLNFAKLDFNILQMLHKKELCDITRWWKELEIVKTLPYVRDRLAEVYF
WSLGVYFEPQYSTARKILTKNISMISLIDDTYDIYGTLDELTLFTEAIERWNIDASQQLQLPSYMKIIYCGLLDVYDEIKKDLANENKSFLI
NYSIIEMKKMVMAYFQEAKWYYGKTIPKMEEYMKSGISTSAYVQVATTSWLGMGNVATKDSFDWIVNEPPILVASSIIARLLNDLLSH
EEEQKRGDAPSGVECYMKEYGVTKEEAHIKIRNTIENSWKDLYEEYFKVNGTIIPRVLLMCIINLARVIEFIY
>SlTPS17
YSIGDLHAIALCFRLLRQQGYYVSCDAFKKFTNDQGNFKEELVKDVEGMLSLYEAAQFRVHGEQILDEALNFTIAQLKQILPKLSNSQ
LAQQITNALKYPIKDGIVRVETRKYISFYQQNQNHNEVLLNFAKLDFNILQTLHKKELSDMTRWWKKMELVNTLPYARDRLVECYF
WCLGTYFEPQYSVARKMLTKISFYISIIDDTYDIYGKLDELTLFTQAIERWNIDASEQLPLYMKIIYRDLLDVYDEIEKELANENKSFLVN
YSINEMKKVVRGYFQEAKWYYGKKVPTMEQYMKNGISTSAYILLTTTSWLAMGNVATKDAFDWVATEPPIVVASCYIIRLLNDLVSH
EEEQKRGNAASAVECYMNEYSVTKEEAHIKIRDIIENYWKDLNEEYFKVDMIIIPRVLLMCIINLTRVAEFIY
>SlTPS18
KNEEIFSNVTHCAMAFRLLRMSYYNVSSDELAEFVDEEHFFSTSGKFISDVAIIELHKASQLTINEKDDILDKINNWTGIFMQQKLLNN
DFLDIKSKKEVELALRMFYVTYDRAENRRYIESYQENNFKMLKTAYRCGSMNNIDLLTFSMQEFELGLSQYQEEVEQLKRWYEDYRL
EQVGLAQEYIYRTHLISVAVFFEHELSNARIMYAKYAMFLTLSDDLFEHLASKDELLNIIELVQRWDEHTNVGFHSEKVKLFFTALYDT
IEEVATNAQIKQGRNVKHHIIELFVEGLNSMLVDRVEWGTRIPSIEEYLRVSLSTFGGKCMVLTSQYVVGIHLCNYQSDDEIQDLCYCS
GIVMRLLNDLQSFKRERSDSRLVNMVKLVMKQRSGTICEEEEEEAIKHIKETIECNRRKLLRMVLQSKGKGSKVPQALKDLFWRTTKA

VYF
>SlTPS19
KNEQIFSNVTHCAMAFRLLRMSYYDVSSDELAEFVDEEHFFATNGKYTSHVEILELHKASQLAIDHEKDDILDKINNWTRAFMEQKL
LNNGFIDRMSKKEVELALRKFYTTSHLAENRRYIKSYEENNFKILKAAYRSPDINNKDLLAFSIHDYELCQAQHREELQQLKRWFEDY
RLDQLGLGERYIHASYLFGVTIIPEPELSDARLMNAKYAMLLTIVDDHFESFASKDECLNIIELVERWDDYASVGYKSEKVKIFFSTFYKS
IEELATIAEIKQGRSVKNHLINLWLEMMKLMLMEQVEWCSGKTIPSIEEYLYVTSITFCAKLIPLTTQYFLGIKISKDLLESDEICGLWNC
SGRVMRILNDLQDSKKEQKEGSITLVTLLMKSMSEEEAVMKTKEILEMNRRELLKMVLVQKKGSQLPQLCKDIFWRSSKWAHF
>SlTPS20
KNEQIFSNVTHCAMAFRLLRMSYYDVSSDELAEFVDEEHFFATNGKYKSHVEILELHKASQLAIDHEKDDILDKINNWTRAFMEQKL
LNNGFIDRMSKKEVELALRKFYTTSHLAENRRYIKSYEENNFKILKAAYRSPNINNKDLLAFSIHDFELCQAQHREELQQLKRWFEDY
RLDQLGLAERYIHASYLFGVTVIPEPELSDARLMYAKYVMLLTIVDDHFESFASKDECFNIIELVERWDDYASVGYKSEKVKVFFSVFY
KSIEELATIAEIKQGRSVKNHLINLWLELMKLMLMERVEWCSGKTIPSIEEYLYVTSITFCAKLIPLSTQYFLGIKISKDLLESDEICGLWN
CSGRVMRILNDLQDSKREQKEVSINLVTLLMKSMSEEEAIMKIKEILEMNRRELLKMVLVQKKGSQLPQLCKDIFWRTSKWAHF
>SlTPS21
KNEEIFSNITHCAMAFRLLRMSNYDVSSDELAEFMDEEHFFTTSGKYTSHVEILELHKASQLAIDQEKDDILDKINNWTRTFMEQKLL
NNGFIDRMSKKEVELALRKFYTTYDRAENRRYIKSYEENNFKILKAAYRSPNINNKDLLIFSIHDFDLCQTQHREELQQLKRWFQDCR
LDQLGLSEQFISTTYLIGIAVVSEPEFSNARLMYAKYVMLLTIVDDLFDGFASKDELLNIIQLVERWDDYASVGYNSERVKVFFSVFYKSI
EELATIAEIKQGRSVKNHLINLWLEVMKMMLIERIEWWTSKTIPSIEEYLYVTSITFGSRLIPLTTQYFLGIKISKDLLESDEIYGLCNCTGI
VMRLLNDLQTYKREQGESSMNLVTILMTQSPRRTNICEEEAIMKIKEILEMNRRELLKMVLVQKKGSQLPQLCKDIFWRTSKMVYF
>SlTPS24
GEDEIFTSAGTCSMAFRILRGYGYNVSSDPVAQFLEQEQYSGHLNDIHTMLDLYQALEMIIATDKPVSMKLNSSSLQSLIQRLSDEFYPP
NGLTKQIREQVDDVLKFPSHANIKRVANRRNIKHYDVDNTRVLKTSYSSSNFGNKDFLTLAVEDFNLCQSIHRNELKQLERWLTQNR
LDKLKFVRERSAYCYFSAAATIFQPELSDARMSWAKNGVLTTVIDDFFDVGGSMEELNNLILLFKKWDVDVSTDCCSERVGIIFSALHS
TISEIGDKASKWQARSVTRHITDIWLNLLNAMLREAEWAKDMSVPSLDKYMANGYVSFALGPIFLPALYFVGPKLPDDVVQHPEYHS
LFELVSTCGRLLNDIRSFERESKDGKLNAVTLSVTHGNGRISEEAAIEGLSHRVEMQRKELLKLVLQREGSVVPNACKDLFWEMSKVL
HQ

>SlTPS25
KTSSTSKDLYATALCFRLLREHGYHASQDMLKDLFDGKGKLPLDMKTSLELFEGSHLSIDGENLLNDIRLFSTKNLKNLSLDVDRLTS
NPLAWRVRWYDVRKHIITAQNCNDTNPMLLKLAKLNFNIIQATHQKDLKDVIRWWRNVSIIENLEFTRERIVESFFFAVGIASEGEHG
SMRKWLAKVIQLILIIDDVYDIYGTLADVQQFTVAIEKWDPEEVQRLPKSIQICFGALHDTMEDISVEIQRQKGGPSVLPHLKQVWVNF
CKALLVEATWYHKGHIPTLEDYLHNGWTSSSGPLLSLHVILGLTNENLHLCKNCQEIIYYTSLIIRLCNDQGTSTVELERGDVASSIICY
MHQENVSEDVAREHIESIILNSWEKTNYHFNRLSTSHRKIMKHVINEARMAHVMY
>SlTPS27
KDSDNEMDLYSTALYFRIFRQYGYNVTQDVFLSYMDEMGEKINVDTNMDPKTMMQLFEASHLALKDENMLDEARIFCTNNLKNIIP
MEMPLHWKVEWYNTREHISKQANEKEEGVSKLKLLQLAKLNFNMVQAEHQKDLVHILRWWRNLGLIENVSFSRDRIVESFLWSVG
VAFEPQHSNFRNWLTKAITFIIVIDDVYDIYGTLQNLQLFTDAVVRWDPKVVEQLPSCMQICFWKLYDTTNDVALEIQQQKGCKFPV
LTYLQKVWAEFCKALLVEAKWDSKGYTPTFSEYLENGWKSSGGTVLSLHVLLGLAQDFSQVDYFLENERDLIYYSSLIIRLGNDLGTST
AELERGDVSSSILCYMRKENVKEDVARKHIEEMVIETWKKMNRHCFENSSPLIKYIMNIARVTHFIY
>SlTPS28
HEEDDLYKVALYFRLFRQHGYPISSDCFNQFKDTKGKFKKTLLIDVKGMLSLYEAAHVREHGDDILEEALIFATFHLERITPNSLDSTLE
KQVGHALMQSLHRGIPRAEAHFNISIYEECGSSNEKLLRLAKLDYNLVQVLHKEELSELTKWWKDLDFASKLSYVRDRMVECFFWTV
GVYFEPQYSRARVMLAKCIAMISVIDDTYDSYGTLDELIIFTEVVDRWDISEVDRLPNYMKPIYISLLYLFNEYEREINEQDRFNGVNYV
KEAMKEIVRSYYIEAEWFIEGKIPSFEEYLNNALVTGTYYLLAPASLLGMESTSKRTFDWMMKKPKILVASAIIGRVIDDIATYKIEKEKG
QLVTGIECYMQENNLSVEKASAQLSEIAESAWKDLNKECIKTTTSNIPNEILMRVVNLTRLIDVVY
>SlTPS3
SEATSANSLYYTALKFRILRQHGFYISQDILNDFKDEQGHFKQSLCKDTKGLLQLYEASFLSTKSETSTLLESANTFAMSHLKNYLNGG
DEENNWMVKLVRHALEVPLHCMMLRVETRWYIDIYENIPNANPLLIELAKLDFNFVQAMHQQELRNLSRWWKKSMLAEKLPFAR
DRIVEAFQWITGMIFESQENEFCRIMLTKVTAMATVIDDIYDVYGTLDELEIFTHAIQRMEIKAMDELPHYMKLCYLALFNTSSEIAYQ
VLKEQGINIMPYLTKSWADLSKSYLQEARWYYSGYTPSLDEYMENAWISVGSLVMVVNAFFLVTNPITKEVLEYLFSNKYPDIIRWPA
TIIRLTDDLATSSNEMKRGDVPKSIQCYMKENGASEEEARKHINLMIKETWKMINTAQHDNSLFCEKFMGCAVNIARTGQTIY
>SlTPS31
FEAHEYNDLCTLSLQFRILRQHGYYISPKIFSRFQDANGKFKESLCDDIRGILNLYEASHVRTHGEDTLEEALAFSTAHLESAAPHLKSP

LSKQVTHALEQSLHKSIPRVETRYFISIYEEEELKNDVFLRFAKLDFNLLQMLHKQELSEVSRWWKDLDFVTTLPYARDRAVECYFWT
MGVYAEPQYSQARVMLAKTIAMISIVDDTFDAYGIVKELEVYTDAIQRWDVSQIDRLPEYMKISYKALLDLYNDYETELSNDGRSDVV
QYAKERMKEIVRNYFVEAKWFIEGYMPPVSEYLSNALATSTYYLLTTTSYLGMKSATKKDFEWLAKNPKILEANVTLCRVIDDIATYE
VEKGRGQIATGIECYMRDYGVSTQVAMDKFQEMAETAWKDVNEGILRPTPVSAKILTRILNLARIIDVTY

>SlTPS32

FEAHDLNTLSLQFRILRQHGYNISQKIFSRFQDANGKFKESLSNDIKGLLNLYEASHVRTHGEDILEEALAFSTAHLESAAPHLKSPLSK
QVTHALEQSLHKSIPRVETRYFISIYEEEEFKNDVLLRFAKLDYNLLQMLHKQELSEVSRWWKDLDFVTTLPYARDRAVECYFWTMG
VYAEPQYSQARVMLAKTIAMISIVDDTFDAYGIVKELEVYTDAIQRWDISQMDRLPEYMKVSFKALLDLYEDYEKELSKDGRSDVVQY
AKERMKEIVRNYFVEAKWFIEGYMPPVSEYLSNALATSTYYLLTTTSYLGVKSATKEDFEWLAKNPKILEANVTLCRVVDDIATYEVE
KGRGQIATGIECYMRDYGVSTQVAMDKFQEMAEIAWKDVNEGILRPTPVSTEILTRILNLARIIDVTY

>SlTPS33

FEAHDLNTLSLQFRILRQHGYNISPKIFCRFQDANGKFKESLSNDIKGLLNLYEASHVRTHGEDILEEALAFSTAHLESAAPHLKSPLSK
QVTHALEQSLHKSIPRVETRYFISIYEEEEQKNDVLLRFAKLDFNLLQMLHKQELSEVSRWWKDLDFVTTLPYARDRAVECYFWTMG
VYAEPQYSQARVMLAKTIAMISIVDDTFDAYGIVEELEVYTDAIQRWDISQIDRLPDYMKISYKALLDLYDDYETELSKDGRSDVVHYA
KERMKEIVRNYFVEAKWFIEGYMPPVSEYLSNALATSTYYLLTTTSYLGVKSATKEDFEWLAKNPKILEANVTLCRVIDDIATYEVEKG
RGQIATGIECYMRDYGVSTQVAMEKFQEMAEIAWKDVNEGILRPTPVSTEILTRILNLARIIDVTY

>SlTPS35

SEAQEYNDLQTSSIQFRLLRQHGYNISPKLFSRFQDAKGKFNESLSNDIKGLLNLYEASHVRTHGEDILEEALAFSTAHLESAAPHLKSP
LSKQVTHALEQSLHKSIPRVETRYFISIYEEEEQKNDLLLRFAKLDFNLLQMLHKQELSEVSRWWKDLDFVTTLPYARDRAVECYFWT
MGVYAEPQYSQARVMLAKTIAMISIVDDTFDAYGIVKELEVYTDAIQRWDISHIDRLPDYMKISYKALLDLYDDYETELSKDGRSDVV
HYAKERMKEIVRNYFVEAKWFIEGYMPPVSEYLCNALATSTYYLLTTTSYLGVKSANKEDFEWLAKNPKILEANVTLCRVIDDIATYD
VEKGRGQIATGIECYMRDYGVSTEEAMEKFEEMAEIAWKDVNEGILRPTPVSTEILTRILNLARIIDVTY

>SlTPS36

KQNDNDNNLYVVSLRFRLVRQQGHYISSDVFKQFMERDGKFKKTLNNDVQALLSLYEAAQIRVRGEDILEEALTFTTTHLESMIPLLS
DNPLKAQIIEALTHPIHKVIPRLGARKYIDIYENMESHNHLLLKFSKLDFNMLQKQHQRELSELTSWWKDLDLASKVPYARDKLVEG
YTWTLGVYFEPQYSRARRMLVKVFKMLSICDDTYDAYATFDELVLFTNAIQRWDINAMDSLPPYMRPFYQAILDIFDELEEELTKEGK

SDRVYYGKFEMKKLARAYFKEAQWLNAGYIPNCDEYIKNAIVSTTFMALGTTSLIGMEEFITKDIFEWITNEPSILRASSTICRLMDDIS
DHESDQQRGHVASVIECYTKEYGASKQEAYVKFRKEVKDAWKGINKALLRPIEVPIFVLQRILNLARTMDTFF
>SlTPS37
CFLKYQNHHDISLCFRLLRQEGYHVSADVFKKLKNNDDGTFGLNLNQDVNGLIGLYEASQLGVEGEYILDEIAKFSGDHLNACLAN
SDEARIIKETLKYPYHKSLSRWKNKSFINNFKGINGWGKNTLKELANMDYFITKEIHQHELAQVFRWWKSLGLAEELKLLRDQPLKW
YTWPMAMLTDPKMSQERIELAKCISFVYVIDDIFDVYGTIEELTLFTQAVHRWELSAMMDLPEYMRSLYKALYNTINSIGYNIYKIYGQ
NPTQNLQNTWAHLCSAFLIEAKWFACGMVPTTDEYLKNGLVSSGVYVALIHLFYILGLGVSSMHLQDISLMSTSIAKILRLWDDLGSA
KDENQEGKDGSYVEYYMKENKDSSMELAREHVIKLIEDEWKQLNKEHFCLMSQSTRSFSKASLNSARMVSLMY
>SlTPS38
KRDHHNEELYDTALEFRLLRQHGYHLPQEIFCSFMNEEGKFKTALVEDTKGLLSLYEASYLCMEDENIMENARDFATHYLMENVKK
KMDEQVSHALEMPVHWRMERLEARWFIEIYHKKENMNPLLLELAKLDYNMVQATYLEELKQMSRWDKNMKLVKKMSFVRDRLV
EGFFWAVGFTPNPQFGYCRKLSTKLSVLLTTIDDIYDVYGTLDELELFTDIVDRWDINAIEQLPEYMKISFLALFNSMNELAYDILKEQG
FSIISHIRKQWANLCKAYLLEVKWYQRGYTPSLDEFLRNAWITNTGPVLIMHAYFCITNPIKEDELQRLNHYPAIIYSPSLILRLANDLA
TSPDEIKKGDYLKSIQCYMHDSKSCEENARNYIKKLIDETWKKMNRDILRDESLSKDFRRTSMNLARIAQCMY
>SlTPS39
CFLKYQNHHDISLCFRLLRQEGYHVSADVFKKLKNNDDGTFGLNLNQDVNGLIGLYEASQLGVEGEYILDEIAKFSGDHLNACLVNS
DEARIIKETLKYPYHKSLSRWKAKSFINNFKGINGWGKSTLQELANMDYSITKEIHQHELIQVSRWWSSLGLAEDLKLLRDQPLKWYT
WPMTMLTDPKMSQQRIELAKCISFVYVVDDIFDVYGTIEELTLFTQAVNRWELCVMKDLPEYMRATYKALYDTINSIGYNIYKIYGQN
PTQNLRNAWANLCNAFLKEAKWFASGELPTTDEYLKNGLVSSGVHVVLVHMFYLLGFGLNNQNSIYLEDSSAMASSVATILRLWD
DLGSAKDENQEGNDGSYIECYMKGQKNASIELAREYVVKLIEDEWKQLNKKHFNLMNGSLGSYSKASLNLARMVPLMY
>SlTPS4
CRDNSLYSTALKFRLLRQHGFHISQDIFNDFKDMNGNVKQSICNDTKGLLELYEASFLSTECETTLKNFTEAHLKNYVYINHSCGDQY
NNIMMELVVHALELPRHWMMPRLETRWYISIYERMPNANPLLLELAKLDFNIVQATHQQDLKSLSRWWKNMCLAEKLSFSRNRLV
ENLFWAVGTNFEPQHSYFRRLITKIIVFVGIIDDIYDVYGKLDELELFTLAVQRWDTKAMEDLPYYMQVCYLALINTTNDVAYEVLRK
HNINVLPYLTKSWTDLCKSYLQEARWYYNGYKPSLEEYMDNGWISIAVPMVLAHALFLVTDPITKEALESLTNYPDIIRCSATIFRLND
DLGTSSDELKRGDVPKSIQCYMNEKGVSEEEAREHIRFLIKETWKFMNTAHHKEKSLFCETFVEIAKNIATTAHCMY

>SlTPS40
ARNSPVQDIDDTAMAFRLLRLHGYAVSADVFKHFESKGEFFCFVGQSNQAVTGMYNLYRASHVMFSGEKILENAKIFTSNYLREKRA
QNQLLDKWIITKDLPGEVGYALDVPWYASLPRLETRFFLEHYGGEDDVWIGKTLYRMPLVNNSLYLELAKSDYNNCQALHQFEWRR
IRKWYYECGLGEFGLSEKRLLVTYYLGSASIFEAQRSTERMAWVKTAALMDCVRSCFGSPQVSAAAFLCEFAHYSSTALNSRYNTEDR
LVGVILGTLNHLSLSALLTHGRDIHHYLRHAWENWLLTVGEGEGEGEGGAELIIRTLNLCSVHWISEEILLSHPTYQKLLEITNRVSHR
LRLYKGHSEKQVGMLTFSEEIEGDMQQLAELVLSHSDASELDANIKDTFLTVAKSFYY
>SlTPS41
ARNSNICEVDTTCMAIRLLRLHGFDVSPDVLHKFKDGDEFFCLRGESNKSATVMFNLYRCSQALFPGEIICEEAKNFTYNFLHQYLAN
NQSKDKWVIAKDIPGEIRYALEFQWYASLPRVESRLYIDQYGGADEIWISKTLYRMPDVSNNVYLEAAKLDYNRCQSQHRFEWLIMQ
EWFEKGNFQKFGISKKEVLVSFFLAASSIFEVEKSRQRLAWAKSCILCKMITSYINQEATTWNSFLMEFKNYRDMSIKKSNETKEIIVLN
NLCQFLHQLTKETYQDLGKDIHHQLHNVWEEWLEENNTTCQEAAVLLVQTINLSSGHMTHDEILSKYTNKVCHMLNEFQNDQIC
NSSKARDIELHMQALVKLVFSNTSSNNINQGIEDTYFKVVKTFYY
>SlTPS5
IAPPDSLYTTALKFRLLRQHGFHISQDILNDFKDENGNLKQSICKDTKDILNSSKDEHDNLKQSTCNNTKGLLKLYEASFLSIENESFLR
NTTKSTLAHLMRYVDQNRCGEEDNMIVELVVHALELPRHWMVPRLETRWYISIYERMSNANPLLLELAKLDFNIVQATHQQDLRIL
SRWWKNTGLAEKLPFSRDILVENMFWAVGALFEPQHSYFRRLITKVIVFISIIDDIYDVYGTLDELELFTLAIQRWDTKAMEQLPDYMK
VCYLALINIINEVAYEVLKNHDINVLPYLTKSWADLCKSYLQEAKWYHNGYKPNLEEYMDNARISIGVPMVLVHSLFLVTNQITKEA
LDSLTNYPDIIRWSATIFRLNDDLGTSSDELKRGDVSKSIQCYMNEKGASEEEAIEHIEFLIQETWEAMNTAQSKNSPLSETFIEVAKNIT
KASHFMY
>SlTPS7
IAPPDSLYTTSLKFRLLRQHGFHISQDVLKDFKDENGNLKQSICKDTKGMLELYEASFLSTETENTLKSATRFTMSHLKNYVDNHSCG
NQDDDIIVELVVHALELPRHWMMPKLETEWYIRIYGRMPNANPLLLELAKLDFNIVQAAHQQDLKILSRWWKSMSLAEKLSFSRDR
LVEDFFWSVGLAFEPQHSLCRRMLAKNVAFIIVIDDIYDVYGSLDELEIFTHAVERWDIKAMEQLPDYMKICYLSLFNTTNEMAYHILK
QQGINVLPYLTKQWTDLCKSYLQEAKWYHNGHKPRLEEYMDNAWISIATPLVLLHAFIFLTNPITQEALESLNNYPDIIRRCAIINRFV
DDLGTSSDELKRGDVPKSIQCYMNDTGASEEEAREHINLLIKEMWEVMNKDQISKQVLFSEEFIKIVFNFSRTSHCVY
>SlTPS8

	KRDHLYAKALEFRLLRQHGFNIVSQETFGGFYDNTTGFGEIHHNEDTKGMLYLYEASFLAIEGEKELELARNLTEEHLREYLADQNK<br>NDVDQNLVELVHHALELPLHWRMLRLETKWFINYYKKRQDKMIPFLLELATLDFNIVQAAHIEDLKYVARWWKETCLAENLPFAR<br>DRLVENFFWTIGVNFLPQYGYFRRIATKVNALVTTIDDVYDVFGTLDELQIFTHAIERWSIDELDRLPDNMKMCYYALDNFINQLAD<br>DAFEEQGIFISPYLRNSWRDLCKSYLREAKWYHSQYIPSMEEYMDNAWISISAPVILVHAYFLVANPVNKEALHYLENNYHDIIRCSAL<br>ILRLANDLGTSSDELKRGDVPKSIQCYMNETQASEEEARQYIRLLISQTWKKLNEAHWLAADPFPKIFVTCAMNLARMAQCMY<br>>SlTPS9<br>KQNDNDNNLYVVSLRFRLVRQQGHYMSSDVFKQFTNQDGKFKETLTNDVQGLLSLYEASHLRVRNEEILEEALTFTTTHLESIVSNLS<br>NNNNSLKVEVGEALTQPIRMTLPRMGARKYISIYENNDAHHHLLLKFAKLDFNMLQKFHQRELSDLTRWWKDLDFANKYPYARDR<br>LVECYFWILGVYFEPKYSRARKMMTKVLNLTSIIDDTFDAYATFDELVTFNDAIQRWDANAIDSIQPYMRPAYQALLDIYSEMEQVLS<br>KEGKLDRVYYAKNEMKKLVRAYFKETQWLNDCDHIPKYEEQVENAIVSAGYMMISTTCLVGIEEFISHETFEWLMNESVIVRASALIA<br>RAMNDIVGHEDEQERGHVASLIECYMKDYGASKQETYIKFLKEVTNAWKDINKQFFRPTEVPMFVLERVLNLTRVADTLY
Selaginella moellendorffii	>Sm76432<br>ELVDAVRTMFRSLGDGNISRSAYDTAWVARVPALDGSNSPQFPMCLDWIIKNQFEDGSWGDKDLFFTYERVCSTLACVISLKIWNTQ<br>EKHIEKAGLEFIRRTMPALETEESAHMLIGFEIVFPAMLDEAMELGLDLDYSSPVVHKFHAEREKKLQRISLDVLQTHPTPLLYSVEGL<br>HKSLDWHKVVKLQCSDGSLLSSPSSTACALMYTGNEKCLQYLNNILERYKDAAVPNTYPVDLFEHIWIVDRIERLGIARYFTREIKDA<br>LDYVYRNWTDKGISWARGTPIQDGDDTSMGFMVLRSHGYDVSADVFKHFQHENEHGFFCFVGQVSEAVTGMLDLYKATKVMFPG<br>DVILQKARAFTRSFLDEKRRKGELNDKWVVTKDLPGEVEFELDNPFHATVERIATRSYIDQYGVDDVWIGKSLYRMPFVNNPVFLEL<br>AKLDFNTCQAFHKQEFKQLERWYAESSFRKFGCYHRDLEQSFFGAAAIIFEPELATARVVWSKCSFIASVIAEYFRRESSIVDLQDLLNG<br>VQR<br>>Sm86551<br>IPATDGGRMWELVETVRSMLNSLHDGEISVSAYDTAWVARVPALDGSNKPQFPMCLNWIMNNQLEDGSWGDRDLFLTYDRICSAL<br>ACAIALKTWNTGDKIVHKALEFIRKTMPKMELEDSTHMPIGFEIVFPAMIEEAMALELDIDYREPVLQTIYAERKKKLERIPMNVVQN<br>YPTTLLHSLEGLHKTIDWDKVIKLQSPDGSLLFSPASTACALMHTGNEKCLQYLNNLVKRFNCAVPNVYPVDLFEHLWIVDRLQRLG<br>ISRYFTQEIKSALDYVYRYWTDKGIAWARGSPVQDADDTSMAFRLLRSHGYDISPDAFKTFQEGDSFVCFSGQAGQAVTGMYNLYRA<br>SQVMFPGETILEEAGSFARKFLEGKRQENQLYDKWIISKDLPGEVEFALDNPMHARLERLATRRYIDQYAADDVWIGKSLYRMPFVN<br>NPIFLELAKADFNMCRALHRKEFQQLERWYDESSLSMFKGFSRSKLEQTFYSAAATIFEPELSPARLIWSQCWFISLGINEYFDHQGSTK

ELEDLINNVERWNVNSLGNCSAEVKILFVELYNIVQNHSKQGFLYQGRSIGGALREIWKTWLSSLLQRTKWKMSDNNPTLEEYLKAS
HSSIEPAVRSTMYFVGETLATTGDIKDSAICQMMNTASRLVQDTHTDKVDSSLNSITIYLEENPQLTKSEALSEVQALANKNMQKLLY
ETLQPGALPQACKQLFLNAARIMNVFPGTNKVQAKLSNHVKRVLSQPVL
>Sm112927
MIEEMRKLLASLDDGEISPSAYDTAWVGRIPSQSNSTCPEFPETLEWIAHNQLPDGSWGDRNHFQIYDRVLSTVSCLVALKTWNLGH
DNINKGSERFLKQNIYKLTKDKGDLLCGFELIFMTMLEEAKQKGLDIPIDIPALKILQGYRQKKLQKIPLEMVHSIPTTILYSLEGLQDHI
NWEKILQFIGTDGSFLSSPSATACVYMHTKDARCLEYLKGVVKKVKNSVPCQYAIDLFERLWIVDTLERLGIDRYFQPEIKNILDYVYK
YWSDKKGIGWGRDSYLKDIDDTSMGFRLLRLHGYKVTPDVFLNFMSSEDKFFCFPGESYHGASDIFNLYRASQVAFANDNILTKAKN
YAHKYLSQLDKAYLDKWSAKKNFFQEVEFELSNQWNSCLPRAYSKSYIHNYGPNDIWIAKTIYRLPFVNNELFINLAKEDFNACQSIH
QSEIQTLLRWWAALKFGDLPFFGDKVVTAHFSIASCMFEPEFSELRLFYTKYALLSSTLDDLADYYGSPAQTRCILEAIRSWDPSLVSHL
SEEVQICFSGLYRTINEMVKSASKVQTGSSIDIREHMQEQLISAQLVDAEWMERKHIPSFETYLSNATVSVGMQDLLLSSIFFCGESISKH
LMQEIKNSRCLQLTCLIARLCNDIGTYQFEREKGEVASSITCYMRENPGITESQAIEHLQGIIDESWKELTEEFLTPSQIPRSIKRLMFETA
RIFQFIYPKKDNFKDPSKAMASLIQNVLYKAAE
>Sm124329
MQAYDTRESPSCKKLPDLINEVRQMLRLVSDGNISMSPYDTAWVARVPSPLNSQQPEFPQCLDWIVENQRQDGSWGDEVFNIYDRIL
CTLSCILALKTWNIHDDVIATGKSNILASTEDKGDLLVGFELIFPPMVEEAKSQGLRIPSNHLFIQLLEELREAKLKGISLDTIHSAPTTVL
FSLEGLQECIDWTQILTLQSEDGSFLTSPSSTACVYLHTKDEKCLTYLRNLVKLHKNAVPDFYPLDMFERLWVVDTLQRLGIDRFFKQE
IKEILDYVYRYWDDKKGIGWARNVSVPELDCSGMGFRLLRLNGYDVSADVFTNFMKDGKFFCFSGERSHGISDVFSLFRASQVAYPKE
AILDQAHAYSQQYLSHLHDHGLDKWAFKNDLSGEVTFELNNAFCSCPPRLYAKSYISKYGTDDSWIAKTIYRLPNVNNAAFLEMAK
QDYDFCRSVLQDETAELSRWWSSTGISFTSKRVKLTDHLVNRLHFQIAAYIFEPELSAMRLAYTKAACLVVACDDLADHFLDAREYQ
ELVASIERWDDPSATKLTDDAKSFYHAVYTTLNELMQEASKACVSTSGIISRQFAVYAKSVVGEVTQRGNTSMPTLEEYLKKSRPRTGI
PAVIICSLPFCGIDMDLETLERASNCKEMELTCDIIRLSNDLATYKVEMENGLSISSITAYLNYCPETSKEDAVKKVTEILDQKTKELIQV
YTQQSKDGLPQLVRRIIFETARTARLVYANGIKESRDELKLPGDDMQTVIRNIIGTKGDVEPSHIN
>Sm163980
MLNSLHDGEISVSAYDTAWVARVPALDGSNTPQFPMCLNWIMNNQLEDGSWGDRDLFLTYDRICSALACAIALKTWNTGDKIVHK
ALEFIRKTMPKMELEDSTHMPIGFEIVFPAMIEEAMALELDIDYTAPVLQTIYAERKKKLERIPMNVVQNYPTTLLHSLEGLHKTIDWD

KVIKLQSPDGSLLFSPASTACALMHTGNEKCLQYLNNLVKRFNCAVPNVYPVDLFEHLWIVDRLQRLGISRYFTQEIKSALDYVYRYW
TDKGIAWARGSPVQDADDTSMAFRLLRSHGYDISPDAFKTFQEGDSFVCFSGQAGQAVTGMYNLYRASQVMFPGETILEEAGSFARK
FLEGKRQENQLYDKWIISKDLPGEVEFALDNPMHARLERLATRRYIDQYAADDVWIGKSLYRMPFVNNPIFLELAKADFNMCRALH
RKEFQQLERWYDESSLSMFKGFSRSKLEQTFYSAAATIFEPELSPARLIWSQCWFLSLGINEYFDYQGSTKELEDLINNVERWNVNSLG
NCSAKVKILFVELYNIVQNHSKQGFLYQGRSIGGALREIWKTWLSSLLQRTKWKMSDNYPTLEEYLKASHSSIEPAVRSTVYFVGETLA
TGDLKDSAICQMMNTASRLVQDTHTDKVDSSLNSITIYLEENPQLTESEALSQVQALANKNMQKLLYETLQPGALPQACKQLFLNA
ARIMNVFPGTNKVQAKLSNHVKRVLSQPVL
>Sm402349
MLMELMQFSSIDDDDDFVQSTMSSDGNRLQHCRSVIVELLLLARRERRAGGRKVGPGVRSAQQAGGLVVSASGIFCIIVSSKPQSDFLS
GVFDFSNPPTGRSLPHSHELLPPLGTLEAVVVGCLLVGAGDLVEKGKLSSSLDCDCDCGCGGKFAISGDDDKLLLALLYWCGSMLNL
SISLCRTRTSYNVETTLTLREGIQGENRSLQRAADDGRSDCLVISRFEGSACIIHLASGERSPARSAFLFSQRECENRSPRTRQNFLLACRT
GQNESTGEHETTELERKGCKCTRPGDVGVYYYFYEIFRNAAELAAAENGRMGVGDGSVGIVSFLWQPLLGQRSRVLERSFANFDDDF
SLGVADTLFVNRSRVETGSYSWSLFLASYRSFSHGSKRNKTPPNTQPASTLDAAAKMLQHGGDAFEIATDSQTSVVVTSCTGSFLLKG
QNQGRREGKAFAMAKVLFSSFQQTGISGSLKSGQLSGVFINGTNLKSNAHAKRFRKNSTSSITISCCASNSPTLENTKLAEAPEKRQKK
KQLPYQGILHVPGDRVEELDTRETSLLVAEVKGWLMKLASGKGEISPSAYDTAWVARIPSESDSSLPEFPEALEWIINSQLPDGSWGDD
RHLQLYDRVLSTLSCLVTLKTWDIGHNSIAQGTKFLRENMIKLKQDDGDLLSGFEVTFPMMLHEAKQLGLDIPYETEFTRLLEISTKKK
LAKIPLDKIHSAPTTLLYSLEGLQDLEIDWQKILKLQSKDGSFLSSPSSTACVYLKTKDRKSLQYLQNAMEDQNYAVPCHYPIDLFESL
WVVDTIERLGIDVFFRDEIKAVLDYVYSFWTNEGIGWGSTCLVNDIDDTAMAFRILRMHGYNVSPDAFNQFWLPGDKFCCFVGELSH
GVSEMLNLHRASQVDFPNEAILTKTFKYSHDYLLNVDSAHMDKWATKKNLMGEVAFELANPFHDCLPRIYNNAYIKHYGMDDLW
IAKTIYRQLANRYAQQCQSYQPSELTELVNWWHSSRFEDVPSTRLTANIDMLPYIYYVICATFHEQEFAQLRVFFSKACCLNTLFDDL
MDCATSIEELDRLQNVIERWDISLSHELPLEYRIPFQEFYNTVLVMTEAASKIHKNLSPEFICKYLSGIYTKLIKSEIADARWKIEGYIPSFE
EYMENAEVSISTWVHIEMKLGQPTQGVSCYMKEHPGATEEDALVYLQSLLEKTKRELNESYFITHENDLPKNIKRFNFEMVRMMLIT
YNETRQLSKTD
>Sm402351
MPGRKHDDHGLLDEWKLVRGCRSRALPSHGSQGGGGLNADDTAYECSHSRLVLTATSKAVVSERQSISSRALQRLTRTSIILLAWRRI
KRLGYWQANEHQVFAQWCTKVDSIADIEGRFLVTQMKYARASKSIALDTVEEEKMKVTITRRFQHTGISGIFKSGQLFGSNLKTNAQI

RKGSLTPSIRITCCASNPLNFGNTKPAEAPEKRQRKQQPYQGILHVPDDRIEELDYRETSLLVEEVKGWQMKLASGKGEISPSAYDTA
WVARIASECDSSLPEFPEALEWIINSQLPDGSWGDDRHLQLYDRVLSTLSCLVTLKTWDIGHNSIAQGTKFLRENMIKLKQDDGDLLS
GFEVTFPMMLHEAKKLGLDLPHETDCLGIPLDKLHSAPTTLLYSLEGLQDLEIDWQEILKLQSKDGSFLSSPSSTACVYLKTKDRKSLQ
YAMQVQNYAVPCHYPIDLFESLWVVDTIERLGIDVFFRDEIKAVLDYVYSFWTNEGIGWGSTCLVNDIDDTAMAFRILRMHGYNVSP
DAFDQFWLPRDKFCCFVGELSHGVSEMLNLHRASQVDFPNETILTKTFKYSHDYLLNVETAHMDKWGNEEKPHGRSNEIQVIFELA
NPFHDCLPRIYNNAYIKHYGIDDPWIGKIIYRLPLVNNKVLLELANWYSQQCQSYQSSELIELTKWDISLSQKLPLKYKIHVQELYNTIL
VMTETASKIHQILSSKFIHNYLSKIYTDLIKSRISAHCRIQGYIPSFKEYMQNAEVSISIGSPVLMSILFCGEPLTEELLNTIHDSKPLKLNNII
FRLCNDNIKGETEEDALSYLQSLLEKNKK
>Sm403761
MSSFSLAQHRRLLLLLLRPRSARGVSSSCARLADDDEKKVSSRLFQRQQVMNAREEDALEEATGPPPKVTTDHVTMSFARSSGAGGQ
NVNKGHGCSVAAPQSQAQAASNGRASRRIIGRGNIEDALEKIQEIIDEACYVPPPPSEETKNRVIKLTCKLHLKTVVLSAISPFRGYTQ
QRVPPDPHLGVSPDGALHEGGHCDFSLSVYPTKLFHLKIEMQEIIEEALQRCKAIAVVLFAEDLELLTVKLRMSFSAIQSSSGFFTQDNK
IKYFHSGRRSKALNVGSFTQFSAGLVLRAGLEVHGTLSIQSHPSSKECEDGSWDQGEEDVQRVCSTLSCVTALKTWNRGECSIQRGVS
FLMSRLPLVHNKLTKDMKAILHRLLQEGKSLDLELNYDESFVQHICEQGQEFLQRVSNDALEQYPTSFVHMLEAFHDRPKWQRLLR
YQSSSGSLFSVTLTACAYMHTGDRKCLRFLNGLLEGPLTKPNKNLSQLLRLIDWLEHLGIERFFEKDIEQTLIQAFRFWRGNAAGSGRQ
QHLKSFEDSALLFRILRRHGYEVSPDVLHQRTFCETRDLKELSLASHFLFPSESRKMLDSIPIIDVANQETTFPWYSIPHRVQHIEYMTKF
MASMDSISMSERASLLAFTKFDFNACQKIYQSDLQRVMRWNGECDFGRLGFARQKEVFCFFTAAATMFQPELSMARIVWAQMSVLT
TVIDDFMDTHDSLDNLRNFCTAVKRWDPSALELNEAARIIFQGLFKTVTWTAECASAVQGRDMTANLRAQWERLCDAFFREAEWR
LTNHCPSLEEYMENGITSFALEPIILSAILFLDGKVESSVLDHPDCINMYVRVSTIGRLLNDIQGFHRESSGGEFSWIAIYMKEHPGTSVE
DAVVEIRKILNATMEELVEDVLRPRAVPKRCKRVFENTMRILNFVYHKGDGFSTDELRGHVKQDTIWYFSVKAGGCDGATGVKTLLP
TFPCGKG
>Sm403764
MSFATQISSGLFAPNNIPSSKRFKPLNMGSFTQFSAGLVLRAGVKVPGTLSVQSHPSSKESSSSTIKKLFAGVDDACGFSAYDMGWIARI
PHAHNPELGPQFPQSLEWIVHNQCDDGSWDQGEEDAQRVCSTLSCVIALKTWNRRECSIQRGLSFLKSQRPLVHDKLTRDMEAILH
RLLQEGKSLDLELNYDESFVQRICEQGQEFLQSVSNDALDQYPTSFVHILEAFHERANWQHLLRYQSSGGSLFSVTLTACAYMHTGD
KKCLRFLNGVLEGPLTKPNKNRHFRGLSQLLRLAEWLEHLGIERFFEKEIEQTLIQAFRYNVFYPSDRLSKAIHVIDVTTFPWYNIMHRI

QHIEYMTKFTASMDFISTSERSSILAFAKSDFNACQKIYQKDLQHVMRWNEECGFGRLGYARQKDVFCVFTAAATMFQPELSMARIV
WAQMSVLTTVIDDFMDTHDSLDNLRNFCTAIRRWDPSASELNDDASILFRGLLKTVTWTAQCASAVQGRDMTASFRAQWERLCDA
FFREAEWRLTNHCPSLEEYVENGITSFALEPIVLSTLLFLDEKIESAVLDHPDCINMCRRVFTIGRLLNDIQGFDRESSAGEFSWITIYMKE
HPGTSVEDAVVEIRKILNATIEELVEDVLRPRAVPKRCKRVFENMMRILNFVYRKGDGYSTDAELRGHIKQAPRMACHSVRLKEILAN
GAENEGDGDRANNSVITEMWSIPAISYKISEDILCFRKSLITGAVWQFILQRDLELQINHQQSN
>Sm406214
MSSFSLAQHRRLLLLLLRPRSARGVSSSCARLADDDEKKVSSRLFQRQQVMNAREEDALEEATGPPPKVTMDHVTMSFARSSGAGGQ
NVNKGHGCSVAAPQSQAQAASNGRVSRRIIGRGNIEDALEKIQEIIDEACYVPPPPSEETKNRVIKLAAAEDQKRLQIKKQIANKKSSR
REQRASFTKNLWYSQQSVPSVDTFNNGVKALSSTRSYQCLAYSKGKFRVRCANMDSGQCRQIPHLGVSPDGALHEGGHCDFSLSVY
PTKLFHLKIEMQEIIEEAFLCFIKAIAVVLFGTAEGLELLTVKLRMSFSAIQISSGFFTQDNKIKYFHSDRRSKALNVGSFTQFSAGLVLRA
GREVHGTLSIQSHLSSKESTIKKLFAGVNDASGFSAYDMGWIARIPHAHNPELGPQFPRSLEWIVHNQCEDGSWDQGEEDVQRVCST
LSCVTALKTWNRGECSVQRGVSFLMSRLPLVHNKLTKDMKAILHRLLQEGKALDLELNYDESFVQYIREQGQEFLQRVSNDALDQY
PTSFVHMLEAFHDRPKWQHLLRYQSSSGSLFSVTLTACAYMHTGDRKCLRFLNGLLEGPLTKPNKNLSQLLRLVDWLEHLGIERFFE
KDIEQTLIQAFRFWRGNAAGSGSQQHLKSFEDSALLFRILRRHGYEVSPDVLHQRTFCETRDLKELSLASHFLFPSESRKMLDSTPIIDV
ANQETTFPWYSIPHRVQHIEYMTKFMTSMDSISMSERASLLAFTKFDFNACQKIYRSDLQRVMRWNEECDFGRLGFARQKEVLCFFT
AAATMFQPELSMARIVWAQMSVLATVIDDFMDTHDSLDNLRNFCTAVKRWDPSALELNEAARIIFQGLFKTVTWTAECASAVQGR
DMTANLRAQWERLCDAFFREAEWRLTNHCPSLEEYMENGITSFALEPIILSAILFLDEKVESSVLDHPDCINMYVRVSTIGRLLNDIQG
FHRESSGGEFSWIAIYMKEHPGTSVEDAVVEIRKILNATMEELVEDVLRPRAVPKRCKRVFENMMRILNFVYHKGDGFSTDELRGHV
KQVLHVKLE
>Sm407280
MSKVLLSSFQRTGGGSLQLFGGTNLKSNAQPNRFRKVSCCASKPAEVLEKREKEKQQPYQGILHVQAGDRIEELDHRETSLLVKEVK
GWLMKLASGKWEISPSTYDTAWVARIPSDSDSSLPEFPEALEWIINSQLPDGSWGTKFLRENMIKLNHADGDFLSGFEVTFPMMLHE
AKRVGLKLPYDTEFTRLLEISTTEKLAKISLDRLHSTPTTLLYSLEGLQGLEIDWQKILKLQSKDGSFFSSPSSTACVYLKTKDKKSLQYLQ
KAMKNQNYAVSCHYPIDLFESLRVVDTIERLGIDLFFRDEIKAVLDYLYSFWTNEGIGWESTCIVNDIEDTAMAFRILRMHGYSVSPDA
FNQFWLPGDKFCCFVGELPHGVSAMLHLYRASQVDFPNEEILTKTFKYSHDYLLNVESSHTSATKKNLMGEVTFELENPLHDCLPRIY
NNAYIKHYSIDDPWIAKTIYRLPHVNNKVLLELANRYAQQCQSYQRSELKILVEWWHSSHFEDIPSTRFKSNVNMLPYIYYVICSTFHE

QNFGQLRIFFTKTCCMNTLFDDLLDSAKSITELDHLQNMIERWDTSFLSQKEYKIIFQEFYNTILVMTEMASKINQNLSPEFIHKYLLAI
GTELIKSGIIDARWKLQGYIPGFEEYMENAEVSIGIATHLLMGILFCGDHLTEELLNIIYDSKLLKLGSIISRICNDIQTYKIEMKLGQSAH
GVSCYMKDHPGETEEDALAYLQSLLEKTKKQLNEIYFTEKDLPKNIKRFNFDIARMMMFTYNKAKQDLFRNPNKELQNMIEFCLKA
>Sm412139
MSKVLLISFQRIGSGSLQLFGGTNLKSNAQPNRFRKVSSCASKPAEALEVKGWLMKLASGKGKISPSTYDTAWVARIPSDSDSSLPEFPE
ALEWIINSQLPDGSWDDDCTKFLRENMIKLNHDDGDFLSGFEVTFPMMLHEAKRVGLKLPYDTEFTRLLEISTTEKLAKIPLDRLHSTP
TTLLYSLEGLQGLEIDWQKILKLQSKDGSLFSSPSSTACVYLKTKDKKSLQYLQKAMKHQNYAVSCHYPIDLFESLRVVDTIERLGIDLF
FRDEIKAVLDNLYSFWTNEGIGWGSTCIVNDIEDTAMAFRILRMHGYSVSPDAFNQFWLPGDKFCCFVGELSHEVSAMLNLYRASQV
DFPNEETLTKTFKYSHDYLLNVESSHTSATKKNLMGEVTVELANPFHDCLPRIYNNAYIKHYSIDDPWVAKTIYRLPLVNKKVLLELA
NRYAQQCQSYQPSELKILVKWWHSSHFEDIPSTRFKANINMLPYIYYVICATFHEQEFGPLRTFFTKTCCMTTLFDDFLDSAKSIRELDS
LQNMIESWDTSFLSQKEYKIIFQEFYNTILVMTKIASKINENLSLEFIHKYLLAIYTKLIKSGIIEARWKIQGYIPGFEEYMENAKVSIAIAT
HVLMGILFCGDPLTEELLNIIYDSKLLKLGSIISRICNDIQTYKIEMKLGESAQGVSCYMKDHPGATEEDAVAYLQSLLEKTKKQLNEIY
FTEKDLPKNIKRFSFDMMMFTYNKAKQDLFKNPNKELQSMIEFCLET
>Sm418910
MPNYYNKDSSLIVTLAKLVDKKSLRQAGRQDSAMASVLFSFKHTGTSASLKSSRAPATRISCCSLSFDNKKPAEAPENQKKKQQPYQ
GILHVPADHVEELDHRETSLLVAEVKGWLMKLASGKGEISPSAYDTAWVARIGSESDSSLPEFPEALEWIISSQLSDGSWGYDRHLQLY
DRVLSTLSCLVTLKLWDIGHSCITQGTKFLRENMIKLKQDDGDLLSGFEVTFPMMLKEAKQLGLDLPYDTEFTRLLEISTKKKLAKIPL
DRLHSSPTTLLYSLEGLQGLEMDWQKILKLQSRDGSFLSSPSSTACVYLKTKDRKSLQYLQKAMEDQNYAVPCHYPIDLFESLWVVDT
IERLGIDVFFRDEIKAVLNYVYSSWTNEGIGWGSTCLVKDIDDTAMAFRILRMHGYNVSSDAFNQFWLPGDKFCCFIGEVSHGVSEML
NLHRASQIDFPNEEILTKTFKYSDGYLLNVESSHMDKWAMKKNLMGEVAFELANPFHDCLPRIYNNAYIKHYGVDDPWIGKTIYRLP
LVNNKVLLELANRYAQQCQSYQPSEMIELVNWWHSSGFENIPSTRLKANINMIPYLYYLICSTFHEEEFGQLRVFFIKTCCINTLFDDLL
DYATNIKELDHLENVIERWDISMSQELSLEYKILFQEFYTIVLVMTEAASKIHQNLSPEFIYKYLLGICIKVIKSIIADARWKIQGYIPSFEE
YMENAEVSVSSWVHILMGIFFCGELLTEELLNTIYDSKPLKLDRIISRLSNDMKTYKIEMKLGQQAQGVSCYMKDHPGATEADALIYL
QSLLNKTKKELNESYFISHQKDLPKNIKRFNFEIVRMILITYNEVRQVDLFKNPDKELQDMIEFSLETYRISNQ
>Sm439448
MSMSTFFKGPLLLTMFAYNYRPEIRQRIDHPIYGNRHVEAKVLLSSFQHIGGGSLQLFGGTNLKSNAQPNRFRKVSCCASKPAEALEK

	REKEKQQPYQGILHVQAGDRIEELDHRETSLLVKEVKGWLMKLASGKWEISPSTYDTAWVARIPSDSDSSLPEFPEALEWIINSQLPDG<br>SWGTKFLRENMIKLNHADGDFLSGFEVTFPMMLHEAKRVGLKLPYDTEFTRLLEISTTEKLAKIPLDRFHSTPTTLLYSLEGLQGLEID<br>WQKILKLQSKDGSFFSSPSSTACVYLKTKDKKSLQYLQNAMKHQNYAVSCHYPIDLFESLRVVDTIERLGIDLFFRDEIKAVLDYLYSF<br>WTNEGIGWESTCIVNDIEDTAMAFRILRMHGYSVSPDAFNQFWLPVDKFSCFVGELSHGVSAMLNLYRASQVDFPNEEILTKTFKYS<br>HDYLLNVENSHTSATKKNLMGEVTFELANPFHDCLPRIYNNAYIKHYSIDDPWIAKTIYQLPLVNKKVLLELANRYAQQCQSYQPSE<br>LKILVKWWHSSHFEDIPSTRFKANINMLPYIYYVICATFHEQEFGPLRIFFTKTCCMNTLFDDILDSAKSITELDSLQNMIESWDTSFLSQ<br>KEYKIIFQEFYNTILVMTKMASKINQNLSPEFIHKYLLAIYTKLIKFGIIEARWKIQGYIPGFEEYMEHAEVSIAIATHVLMGILFCGDPLT<br>EELLNIIYDSKLLKLGSIISRICNDIQTYKIEMKLGESAQGVSCYMKDHPGATEEDALAYLQSLLEKTKKQLNKIYFTEKDLPKNIKRFSF<br>DMARGMMFTYNKAKQDLFKNPNKELQSMIELCLET

TPS proteins from *P. equestris* and *A. shenzhenica* were obtained from their genome database [25]. The other TPS proteins were downloaded from Phytozome version 12.1 database (http://phytozome.jgi.doe.gov/pz/portal.html).

Table S7 The FPKM values of *DoTPS* genes in different *D. officinale* organs.

Gene ID	Fb	Se	La	Po	Gs	St	Le	Ro	Rs	Rw
DoTPS1	1.2173	12.3391	1.7087	0.0947	0.1462	0.0000	0.0506	0.0000	0.0000	0.0000
DoTPS2	0.6080	5.9884	3.2341	0.0000	1.5680	0.9939	0.0319	0.3406	0.1358	0.0000
DoTPS3	8.3954	34.6934	17.0447	0.0000	7.0096	8.1833	0.0000	2.3638	0.3534	0.1604
DoTPS4	1.8939	1.3733	1.1464	0.0000	4.1851	3.5203	0.3542	7.8949	23.4339	16.4147
DoTPS5	0.7914	13.2949	152.6900	0.0448	2.5230	0.0000	0.1076	9.6566	0.8778	15.8660
DoTPS6	0.0400	0.0000	0.0421	0.0000	0.0000	0.0000	0.0000	0.7572	3.9059	0.0000
DoTPS7	0.4317	14.0691	49.8742	0.0895	1.2788	0.2708	9.2217	3.6585	0.9160	0.6236
DoTPS8	9.4823	6.6341	11.0165	0.5050	23.4320	5.9896	0.5539	1.3638	1.1329	0.6170
DoTPS9	4.0618	0.2621	0.0211	0.0374	4.0561	1.2428	0.0000	7.9739	4.5215	7.8422
DoTPS10	0.0000	1028.7356	3268.7298	0.0632	145.0056	0.0956	0.0676	0.0000	0.0000	0.0000
DoTPS11	0.0000	0.0000	0.0000	0.0000	0.0000	0.0823	0.0000	0.0000	0.0000	0.0000
DoTPS12	0.0000	0.0000	0.1695	0.0000	0.0000	0.0000	0.0000	0.1904	2.0500	0.0000
DoTPS13	0.0000	0.1373	0.0000	0.0228	0.0000	5.2483	0.1830	7.2889	3.1529	0.7420
DoTPS14	3.3417	2.8140	2.2112	3.4162	2.7519	1.5051	0.6905	4.7703	3.8745	2.8503
DoTPS15	0.0000	0.0000	0.0772	0.0000	0.0000	0.0000	0.0000	6.8117	24.9504	0.6713
DoTPS16	0.2951	0.0000	4.8930	118.7210	2.5872	0.0694	0.0000	0.0000	0.0783	0.0710
DoTPS17	0.0000	0.1034	0.0000	0.0000	0.4953	759.8893	22.0740	0.0000	0.0781	0.5673
DoTPS18	0.0727	0.0680	1.3780	0.3394	0.5240	0.0000	0.2176	14.4042	1.3501	0.2451
DoTPS19	0.0842	4.0566	14.2685	0.0000	9.7867	78.5955	0.6298	0.3982	0.0447	0.0000
DoTPS20	2.0532	6.7076	29.2717	0.3681	14.9119	6.1246	18.3284	2.9219	0.5169	0.8713
DoTPS21	0.0390	100.6108	2716.5036	0.0243	81.5684	0.0000	0.0000	0.0000	0.0000	0.0376
DoTPS22	0.0719	15.6847	616.6676	0.0000	5.2533	0.0000	0.0000	16.0802	10.3427	68.1060
DoTPS23	0.1091	16.3636	61.3193	1.1765	38.9150	0.1711	0.4715	7.7396	1.5816	0.3501
DoTPS24	0.3074	219.5391	153.9008	0.0273	18.3111	0.2480	0.0438	4.6220	0.6057	0.4229
DoTPS25	19.2802	4.3919	0.0817	0.0000	3.5780	6.8637	0.0387	93.8604	23.4594	19.3139
DoTPS26	0.0369	0.0000	1.2815	1.9052	0.6734	0.0000	0.0000	0.0436	0.0000	0.0000
DoTPS27	0.0000	7.6697	3.0748	0.0000	0.3319	0.0000	0.0313	0.0000	0.0333	0.0000
DoTPS28	260.6178	29.7739	0.9286	0.0203	27.5280	3.6897	0.3585	12.3247	7.6256	8.0543
DoTPS29	0.8101	0.0345	0.0388	0.0458	0.0000	0.2426	0.0000	0.0000	0.9766	0.0355

DoTPS30	0.2087	0.1562	0.3955	0.0000	0.1203	0.3536	0.0000	18.7103	4.4290	9.8092
DoTPS31	28.8962	19.5584	38.1274	68.4811	38.0720	29.1411	16.1677	23.9266	19.2718	24.4360
DoTPS32	13.7093	6.9285	5.0302	9.2781	10.4072	32.5144	24.9788	41.6248	37.2907	25.5609
DoTPS33	1.7942	6.8443	0.8718	0.0286	0.8840	2.6845	0.2753	0.0000	0.0000	0.2215
DoTPS34	0.7452	0.0634	0.1070	0.0211	0.4230	0.1275	0.0000	13.9790	1.1140	33.6942

All FPKM values were downloaded from NCBI under BioProject PRJNA262478 [25].The different tissues were sepals (Se), labellum (la), green root tips (Rt), roots (Ro), white part of roots (Rw), pollinia (Po), flower buds (Fb), gynostemium (Gs), stems (St) and leaves (Le) in two-year-old *D. officinale* adult plants.

Table S8 The expression values of *DoTPS* genes in response to 200 mM mannitol treatment for 48 h.

GeneID	Mannitol-0h	Mannitol-12h	Mannitol-24h	Mannitol-48h
DoTPS1	0.1276	0.1598	0.0000	0.0000
DoTPS2	0.3753	0.3901	0.5617	0.2568
DoTPS3	0.3959	0.1410	0.0564	0.0580
DoTPS4	0.0052	0.0223	0.0877	0.0476
DoTPS5	1.3909	1.8039	1.7631	2.1192
DoTPS6	0.0752	0.0559	0.0333	0.0238
DoTPS7	0.0803	0.4008	0.4808	0.3799
DoTPS8	1.4286	0.0000	0.1358	0.0000
DoTPS9	0.0764	0.0954	0.0763	0.0784
DoTPS10	0.3944	0.6160	0.2956	0.4044
DoTPS11	0.1605	0.1372	0.0016	0.0067
DoTPS12	0.0000	0.0003	0.0003	0.0000
DoTPS13	1.2635	1.1821	2.8364	0.1297
DoTPS14	0.0000	0.4826	0.1544	0.0000
DoTPS15	0.0674	0.0873	0.3506	0.1410
DoTPS16	3.2468	0.0000	0.2592	0.0666
DoTPS17	1.6198	1.3183	1.8725	2.0104
DoTPS18	7.9091	1.2334	0.7234	0.2029
DoTPS19	0.0072	0.0000	0.0000	0.0000
DoTPS20	0.0000	0.0000	0.1637	0.0000
DoTPS21	1.0867	1.0439	1.0018	0.7717
DoTPS22	0.0994	0.0000	0.0000	0.0000
DoTPS23	2.0970	0.1539	0.0615	0.0000
DoTPS24	0.3125	0.2748	0.2256	0.0000
DoTPS25	2.4727	2.2591	1.7555	1.8702
DoTPS26	0.0036	0.0030	0.0000	0.0000
DoTPS27	0.1819	0.0567	0.0000	0.1400
DoTPS28	0.1157	0.1900	0.1350	0.1482
DoTPS29	0.0000	0.9553	4.0462	2.3205
DoTPS30	0.1083	0.1157	0.1886	0.1455
DoTPS31	0.3650	0.2843	0.0910	0.0937
DoTPS32	0.2708	0.0000	0.0000	0.0926
DoTPS33	0.0569	0.0637	0.0965	0.1391
DoTPS34	0.0000	1.9598	0.2137	2.7829

Table S9 The FPKM values of *DoTPS* genes exposed to cold treatment (0°C) for 20 h.

Gene ID	CK1	CK2	CK3	CA1	CA2	CA3
DoTPS1	0.2354	0.0000	0.0000	0.0000	0.0000	1.4929
DoTPS2	0.0000	0.0000	0.0000	0.1970	0.0655	0.0000
DoTPS3	0.0244	0.0000	0.0000	0.8976	0.1642	0.1211
DoTPS4	0.5900	0.3483	0.2700	0.0000	0.0494	0.0000
DoTPS5	0.0577	0.0000	0.0633	12.4024	0.0000	0.0648
DoTPS6	0.0000	0.0000	0.0000	0.0278	0.0000	0.0000
DoTPS7	2.6395	3.4961	2.5775	3.7131	1.8002	4.8149
DoTPS8	0.2326	0.1741	0.2829	0.1317	0.1332	0.0485
DoTPS9	0.0000	0.0000	0.0000	1.1013	0.8665	0.7629
DoTPS10	0.0572	0.0000	0.5597	0.0000	0.0000	5.2860
DoTPS11	1.6998	0.5873	0.9166	5.1072	2.2991	2.4442
DoTPS12	0.0000	0.0000	0.0000	0.0648	0.0633	0.0577
DoTPS13	0.1689	0.0000	0.0371	0.0402	0.0000	0.0000
DoTPS14	0.6177	0.4155	0.4545	1.6973	1.8028	1.0530
DoTPS15	0.0000	0.1376	0.3573	0.0000	0.2571	0.2266
DoTPS16	0.1834	0.0000	0.0965	15.0021	3.5563	4.0863
DoTPS17	0.0000	0.0000	0.0000	0.0268	0.0000	0.0100
DoTPS18	0.9556	0.6541	0.4678	1.2828	1.0808	1.0943
DoTPS19	0.1200	0.0229	0.1218	0.1151	0.1200	0.1753
DoTPS20	1.2828	0.6541	0.4678	0.9556	1.0808	1.0943
DoTPS21	1.2114	0.4076	1.3413	3.3646	2.1892	1.5833
DoTPS22	0.0000	0.0000	0.0000	0.0260	0.0260	0.0000
DoTPS23	0.8701	0.9705	0.7816	0.0611	0.5352	0.0100
DoTPS24	0.0000	0.0000	0.0000	0.1234	0.0274	0.0900
DoTPS25	0.2291	0.1046	0.0000	0.0645	0.2141	5.5846
DoTPS26	0.0258	0.0800	0.0500	0.0000	0.0000	0.0000
DoTPS27	0.0000	0.0000	0.0000	0.0220	0.0000	0.0204
DoTPS28	0.0257	0.0453	0.0239	0.5664	0.3466	0.3113
DoTPS29	0.2685	0.1499	0.2396	12.2277	1.1678	0.1228
DoTPS30	0.0000	0.0000	0.1712	0.5352	0.8701	0.7816
DoTPS31	8.4991	7.3799	7.5907	12.3982	14.3222	10.5718
DoTPS32	14.8624	10.7031	11.3577	11.9571	11.8444	13.2613
DoTPS33	0.0900	0.0374	1.1500	0.0000	0.0000	0.0000
DoTPS34	0.0000	0.0414	0.0000	0.2396	0.1499	0.2685

All FPKM values were retrieved from a reported transcriptome database [29]. Treatments was made up of cold acclimation (0°C for 20 h, CA) and non-acclimation (20°C for 20 h, CK).

Table S10 The expression values of *DoTPS* genes in response to 200 mM mannitol treatment for 48 h.

Gene ID	MeJA-0 h	MeJA-12 h	MeJA-24 h	MeJA-48 h
DoTPS1	0.1933	1.6602	1.1917	0.0750
DoTPS2	0.8195	13.8851	8.6327	2.4095
DoTPS3	6.1303	39.9956	16.9366	4.3490
DoTPS4	1.2011	21.9405	20.1309	0.4633
DoTPS5	0.4101	1.2579	1.2293	0.8729
DoTPS6	0.0361	0.3785	0.6501	0.0916
DoTPS7	0.1185	2.7562	1.8431	0.3405
DoTPS8	23.5785	67.9417	85.4984	33.8374
DoTPS9	0.8135	36.7045	21.6445	2.4708
DoTPS10	0.5484	13.8014	20.4691	0.5889
DoTPS11	0.1555	0.1789	0.1521	0.0499
DoTPS12	0.1481	0.1598	0.1419	0.0958
DoTPS13	26.8832	156.4808	140.3864	47.9339
DoTPS14	0.1290	0.8303	0.4973	0.1922
DoTPS15	0.1598	0.5396	0.6297	0.2608
DoTPS16	0.4317	3.5311	4.6591	0.3621
DoTPS17	2.5886	5.6565	4.4752	3.8197
DoTPS18	9.7912	35.5316	30.2294	5.3614
DoTPS19	0.5337	0.9874	0.4997	0.5743
DoTPS20	0.2734	0.3123	0.7531	0.1291
DoTPS21	0.7547	25.4705	34.7946	0.6974
DoTPS22	1.5664	10.0653	6.8606	0.2356
DoTPS23	5.7882	11.4549	9.8674	2.5557
DoTPS24	0.9002	0.9873	0.8631	0.7432
DoTPS25	0.0566	0.8189	0.7273	0.1050
DoTPS26	5.5219	10.1560	12.4607	23.2561
DoTPS27	4.5398	22.5801	9.6888	4.7090
DoTPS28	6.8520	4.5228	4.9056	5.8451
DoTPS29	9.5300	17.8871	15.5421	11.6043
DoTPS30	0.3827	1.0406	1.0023	0.6329
DoTPS31	1.1175	0.4629	0.6216	1.5712
DoTPS32	0.9362	0.4098	0.5825	0.8163
DoTPS33	1.2334	2.3022	3.4734	1.7218
DoTPS34	0.0345	0.7113	0.2138	0.0780

Table S11 The expression values of *DoTPS* genes in at three flowering stages of *D. officinale*.

	B1	B2	B3	S1	S2	S3	F1	F2	F3
DoTPS1	0.8354	0.8348	0.5920	5.5536	6.1455	5.1733	43.3164	37.8553	44.3107
DoTPS2	0.0771	0.0000	0.0000	0.2770	0.1674	0.6346	0.4768	0.7810	0.6779
DoTPS3	3.9852	4.8716	3.3371	17.1842	22.9828	20.7984	24.9471	29.9034	28.2281
DoTPS4	0.0000	0.0395	0.0000	0.1083	0.1239	0.1783	0.0389	0.0000	0.0000
DoTPS5	0.0000	0.0000	0.6293	0.3093	1.0851	0.0000	0.6199	0.0000	0.2888
DoTPS6	0.0057	0.0040	0.0044	0.0040	0.0044	0.0040	0.0044	0.0000	0.0000
DoTPS7	2.9934	2.2824	2.7800	0.9494	1.1183	1.5537	0.2612	0.5734	0.6133
DoTPS8	0.0000	0.0006	0.0000	0.4696	0.3430	0.2595	0.0000	0.3431	0.0299
DoTPS9	0.0120	0.0134	0.0112	0.0000	0.0040	0.0044	0.0000	0.0004	0.0000
DoTPS10	223.2744	229.3890	216.2026	3234.7494	3217.9300	3179.3628	891.4052	652.9398	630.1163
DoTPS11	0.0090	0.0047	0.0035	0.0040	0.0037	0.0040	0.0044	0.0000	0.0000
DoTPS12	0.0000	0.0003	0.0001	0.0000	0.0000	0.0000	0.1042	0.0000	0.0000
DoTPS13	0.0000	0.0000	0.0000	0.1851	0.2798	0.2012	0.8726	0.1745	0.2143
DoTPS14	0.8456	0.7260	1.1031	0.3094	0.3188	0.3702	4.7988	4.8901	4.5217
DoTPS15	0.0003	0.0000	0.0003	0.0287	0.0295	0.0000	0.0002	0.0004	0.0000
DoTPS16	2.3237	2.7277	2.4948	0.0000	0.2986	0.0324	0.5068	0.0328	0.3382
DoTPS17	0.0023	0.0013	0.0022	0.0040	0.0044	0.0000	0.0040	0.0070	0.0000
DoTPS18	0.0807	0.0640	0.1211	0.0000	0.0011	0.0000	0.0053	0.0000	0.0023
DoTPS19	5.4240	5.0955	6.1791	17.2144	19.5407	18.3601	11.1548	12.3520	10.0018
DoTPS20	14.2348	15.0919	15.4891	13.7248	13.6509	14.0405	4.4426	6.9192	5.0905
DoTPS21	84.8295	77.6592	88.9460	627.2865	656.8730	774.5678	139.1584	134.5610	130.4231
DoTPS22	0.1041	0.0000	0.0000	0.0000	0.0000	0.0000	0.0000	0.0000	0.0000
DoTPS23	15.3781	18.0934	14.8088	13.3106	20.8759	12.2737	3.9390	3.5389	3.4028
DoTPS24	7.3896	7.8489	9.5586	114.7701	102.1070	112.7620	160.4668	196.0226	135.9785
DoTPS25	0.0761	0.0177	0.0583	1.0937	0.8031	0.0239	0.0332	0.1064	0.0893
DoTPS26	0.3991	0.3511	0.9803	0.0000	0.0117	0.0641	0.0000	0.0001	0.0000
DoTPS27	0.0012	0.0000	0.0012	0.0067	0.0038	0.0000	33.3199	37.7800	35.2909
DoTPS28	249.8680	227.5599	232.3214	2.4423	2.9088	3.2606	0.6343	0.5608	0.4010
DoTPS29	0.0003	0.0007	0.0005	0.0000	0.0017	0.0270	0.0691	0.0514	0.1174
DoTPS30	0.0466	0.0000	0.0266	0.0367	0.0000	0.0000	0.0003	0.0001	0.0000
DoTPS31	16.6555	12.2840	15.7901	12.6694	6.0019	25.8597	2.7267	8.9457	7.8824
DoTPS32	10.8991	10.4409	10.5708	3.0542	5.2010	4.7502	5.1282	4.6255	5.3851
DoTPS33	1.1276	0.4167	0.9774	2.3825	4.2775	5.0569	24.3645	21.7366	24.4755
DoTPS34	0.0167	0.0709	0.1117	0.0000	0.0004	0.0003	0.0001	0.0002	0.0000

Three flowering stages of *D. officinale* include budding (B), semi-flowering (S) and full flowering (F).

Table S12 Functional annotation of DoTPS with Terzyme (http://www.nipgr.ac.in/terzyme.html).

Gene sequence ID	Score	E-value	No. of domains
Putative monoterpene synthase			
DoTPS28	657.1	$3.8\times e^{-200}$	1
DoTPS10	649.4	$8.4\times e^{-198}$	1
DoTPS21	643.2	$6.2\times e^{-196}$	1
DoTPS34	642.9	$7.5\times e^{-196}$	1
DoTPS25	637.0	$4.7\times e^{-194}$	1
DoTPS23	631.6	$2.0\times e^{-192}$	1
DoTPS18	622.9	$8.7\times e^{-190}$	1
DoTPS20	619.6	$9.0\times e^{-189}$	1
DoTPS27	604.7	$2.9\times e^{-184}$	1
DoTPS3	604.0	$4.6\times e^{-184}$	1
DoTPS2	598.2	$2.6\times e^{-182}$	1
DoTPS33	554.1	$5.9\times e^{-169}$	1
DoTPS30	534.5	$5.2\times e^{-163}$	1
DoTPS19	477.7	$8.0\times e^{-146}$	2
DoTPS24	394.2	$1.6\times e^{-120}$	1
DoTPS1	233.4	$7.4\times e^{-72}$	1
Putative sesquiterpene synthase			
DoTPS9	701.4	$1.6\times e^{-213}$	1
DoTPS5	673.8	$3.2\times e^{-205}$	1
DoTPS29	672.6	$8.1\times e^{-205}$	1
DoTPS7	670.8	$2.6\times e^{-204}$	1
DoTPS22	669.9	$4.8\times e^{-204}$	1
DoTPS17	668.3	$1.7\times e^{-203}$	1
DoTPS6	663.6	$4.3\times e^{-202}$	1
DoTPS26	661.8	$1.4\times e^{-201}$	1
DoTPS16	658.7	$1.3\times e^{-200}$	1
DoTPS15	637.5	$3.5\times e^{-194}$	1
DoTPS13	615.2	$2.1\times e^{-187}$	1
DoTPS11	309.7	$6.2\times e^{-95}$	1
DoTPS8	211.9	$2.7\times e^{-65}$	1
DoTPS12	156.5	$1.6\times e^{-48}$	2
Putative diterpene synthase			
DoTPS32	1029.5	0.0	1
DoTPS4	622.0	$2.4\times e^{-189}$	1
DoTPS14	592.3	$2.3\times e^{-180}$	1
DoTPS31	249.0	$1.5\times e^{-76}$	2

Table S13 CGTCA-motif, G-box and MYC motif in the promoter region of *DoTPS* genes.

Gene	CGTCA-motif		MYC motif		G-box	
	Number	Position	Number	Position	Number	Position
DoTPS1	1	+638	4	-327, +207, +474, +1329	4	-884, -1065, +636, +1140
DoTPS2	2	-1464, -1756	4	-469, -505, -710, +576	1	-209
DoTPS3	3	-331, -1745, +293	8	-18, -122, -1149, -1182, -1304, -1591, +1040, +1197	4	-998, -1835, +455, +1834
DoTPS4	0		1	-294	1	+684
DoTPS5	3	+832, +858, +1870	7	-77, -1187, -1412, +31, +69, +83, -730	2	+1868, +1916
DoTPS6	1	+95	4	-992, -1154, -1800, -1911	5	-341, -365, -724, +724, +1548
DoTPS7	1	+312	3	-212, -629, +70	3	+652, +698, +762
DoTPS8	3	-468, -789, +419	3	-1152, -1282, +924	0	
DoTPS9	0		7	-26, -1582, -1614, -1654, +128, +1628, +1978	2	-241, -1513
DoTPS10	2	-737, -782	7	-548, -687, -1049, -1129, -1470, +216, +519	3	-93, -1114, -1848
DoTPS11	0		1	-506	0	
DoTPS12	1	-1933	2	-1159, +845	3	-1548, +1229, +1545
DoTPS13	0		2	-781, +1090	1	-638
DoTPS14	1	-1150	7	-290, -359, -1805, -1913, +246, +673, +1320	2	-621, -1151
DoTPS15	0		5	-234, -1269, -1466, -1860, +159	2	+899, +900
DoTPS16	7	-53, -967, -981, -1024, +1045, -1218, +886	5	-49, -889, -1258, +902, +995	0	
DoTPS17	1	+1511	4	-427, -821, -1650, +414	2	-483, +612
DoTPS18	0		3	-1288, +633, +1501	1	-1910
DoTPS19	1	-1048	5	-276, -1252, +1399, +1835, +1927	0	
DoTPS20	2	-663, +1720	7	-222, -785, -833, +191, +690, +1079, -1112	0	
DoTPS21	1	-1093	6	-1154, -1465, -1646, -1692, +165, +518	1	-1223
DoTPS22	2	+741, +750	1	+175	1	+1916
DoTPS23	1	-1877	4	-584, +124, +771, +1346	2	-1052, -1878

DoTPS24	0		2	+797, +1950	0	
DoTPS25	7	-175, -1072, -1209, -1337, +583, +677, +1000	4	-384, +72, +1733, +1783	4	-1666, +613, +1066, +1225
DoTPS26	0		2	-669, +115	1	+662
DoTPS27	2	-1544, +1112	4	+436, +726, +1230, +1951	2	-1545, +976
DoTPS28	0		4	-630, -664, -1403, +1901	5	-156, -180, +183, +203, +489
DoTPS29	0		1	-431	0	
DoTPS30	1	+1443	6	-1000, -1448, +282, +495, +968, +1663	1	+231
DoTPS31	1	-774	1	+183	1	-1713
DoTPS32	2	+875, +906	1	-1212	3	-585, -1106, +587
DoTPS33	0		3	-1336, -1503, +1781	1	+1626
DoTPS34	1	-651	4	-468, -1502, -1715, +973	2	-955, +1451

Table S14 Primers used for RT-qPCR analysis, DoTPS10 cloning and vector construction.

Gene		Primer
DoTPS1	F	CCTCACTCTTCCATGAGGAAAT
	R	CGAAGAAGCTGGAAGCAAATAG
DoTPS2	F	AGCACAGCGCTCAGTATTT
	R	TCTTCGAAAGGAAGTGGAAGAG
DoTPS3	F	GTAATCTAGCTGGTGGTGAGTT
	R	GTTGAGAAACGAGGTTCCATTG
DoTPS4	F	ACATTGAAGGAGGAGGGATCTA
	R	GCAAGTTGGAGGAGGTCATTAT
DoTPS5	F	TGTTTATGGGACAGTCGAAGAG
	R	GGAACTCGTGTTGCATGTATTG
DoTPS6	F	ACACTTATTACTGCTGGCTACTC
	R	TGATCTCTGGGAAGCTTGTAAC
DoTPS7	F	GGGTAACTGGTATGCCTCTAATC
	R	TAGCATTGGACAGCTGAAGG
DoTPS8	F	GAAGTCATTGATCTGCAAGATGG
	R	CTTGAACCGATCAGGTGTTCTA
DoTPS9	F	ACACTTATTACTGCTGGCTACTC
	R	GGAAGCTTGTAACCCAATCAAAG
DoTPS10	F	GCTGCCTCTCAACAGCTAATA
	R	CTGGAAGCGATTGGATGTAGT
DoTPS11	F	CAGAGTGGTGGAAAGGATTAGG
	R	GTGAATAGCAGGGTTCAGAGTAA
DoTPS12	F	CTACTGAGGCAGCAACGATTTA
	R	TCAAAGTATGTGCCTGTCATCC
DoTPS13	F	CGATGAGCATGAAGAGAGAGAC
	R	CCATGCATCCTCCACCATTA
DoTPS14	F	AGTCATTGTGGTTCGAGGAAG
	R	CAGATACTGGTACGAAGGCATAC
DoTPS15	F	AGCATCTAGGAGTGGCCTAT
	R	AATCGTTGCTGCCTCAGTAG
DoTPS16	F	GATCTGCTAGCTGTTGCTCTAA
	R	TGCTCTTAAACCTCCCTTCATC
DoTPS17	F	TAGGCTTCTCCAAACACTTACC
	R	GGGCATCACCTTTGACATAATC
DoTPS18	F	TGATGCTTATGAGAGGCAAGAG
	R	TCATCGCTAGCAAGACCAATAC
DoTPS19	F	GCAAGAGATAGGTTGATGGAGAC
	R	TGAGGCAACCTAGTTTGGTAATC
DoTPS20	F	GCCTTTACGAGGCTTCTTATCT
	R	TTGTTCCACTAACGAGGGATG
DoTPS21	F	GCCAACCATTTGGGATGATAAC
	R	AAACACCTGACTTCCTCCTTC

DoTPS22	F	ACCTCAAAGAGGAGGAGAAGA
	R	AAGGCTGACGGCCAAATAA
DoTPS23	F	GCTTATGAGAGGCAAGAGAACA
	R	CCACCAACTAGACATCTCCTTAAC
DoTPS24	F	TTACAAGGTGGTGGGCAAA
	R	TGCTAAACCTACGGCTAAGAAG
DoTPS25	F	CTTGTATGAGGCTTCCTTCCTT
	R	GTAGCTCCAAGGCATGTTCTAT
DoTPS26	F	TGAGAGAGAGGGTGGAAGAA
	R	GCCTAGGCGTTGAAGAGAAT
DoTPS27	F	AAGTGGTGGGCAGAAGTAAG
	R	CAGCCATACCAACAGCTAGAA
DoTPS28	F	CCTCACCATCGCTTCCTATTC
	R	AGCAGAAGAAGATGTTGCAGTA
DoTPS29	F	TCATGCGTACCTTCAGGAATC
	R	GAGTACCCAGCTGTAATCAGTG
DoTPS30	F	ATAGATCACGCCTTGGAGTTAC
	R	AAAGTTCTCTCGTCTCCCATAAG
DoTPS31	F	CTGAAGCTGCCGATCCATATT
	R	GCTCAATCGACCGAACTAACA
DoTPS32	F	CAAACACTCAGCGCAAACAT
	R	ACATACTCCTCACCAACATTCC
DoTPS33	F	GCAACGACTTAGCAACATCATC
	R	GATATGTACAGGCGAGCTTCTT
DoTPS34	F	TGCTTATGGGAGACGAGAGA
	R	GTTCTTGTAGCTCCGCCTTAT
DoEF-1α	F	GCTTGAGAAGGAGCCCAAGT
	R	CCAACAGCCACAGTTTGTCG
DoTPS10-ORF	F	ATGGCTTTGTATTTTCGCCCT
	R	AAGTGGAATTGGTTCAATCATCA
DoTPS10-pET32a	F	TTCGAGCTCCGTCGACATGGCTTTGTATTTTCGCCCT
	R	GGTGGTGGTGCTCGAGAAGTGGAATTGGTTCAATCATCA
DoTPS10-YFP	F	CGAACGATAGCCATGGATGGCTTTGTATTTTCGCCCT
	R	TGAGTCCGGACCATGGAAGTGGAATTGGTTCAATCATCA

Gene-specific primers for real-time reverse transcription quantitative PCR (RT-qPCR) were designed by the PrimerQuest online tool (http://www.idtdna.com/Primerquest/Home/Index). The *D. officinale* actin gene (DoEF-1α) was obtained from NCBI (GenBank accession no. JF825419). F, forward; R, reverse.

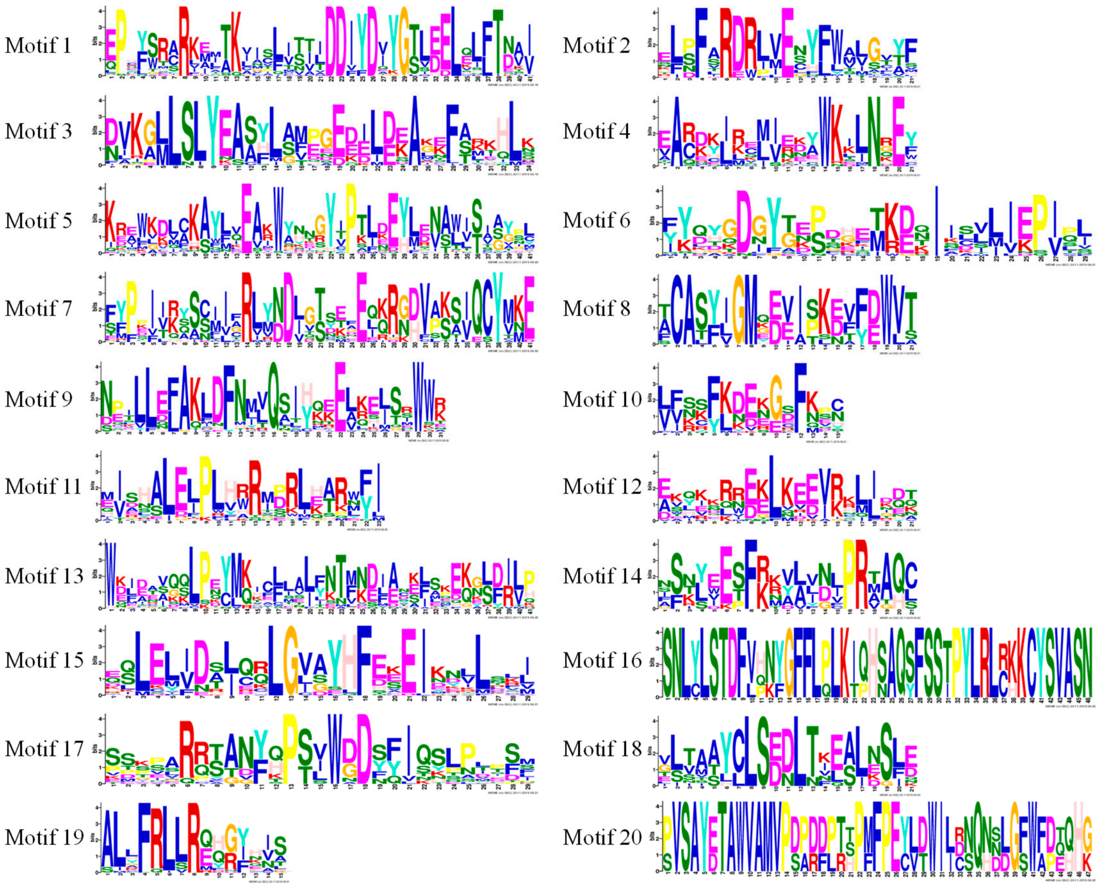

Figure S1 Distribution of conserved motifs in *D. officinale* TPS proteins based on the results of MEME analysis.

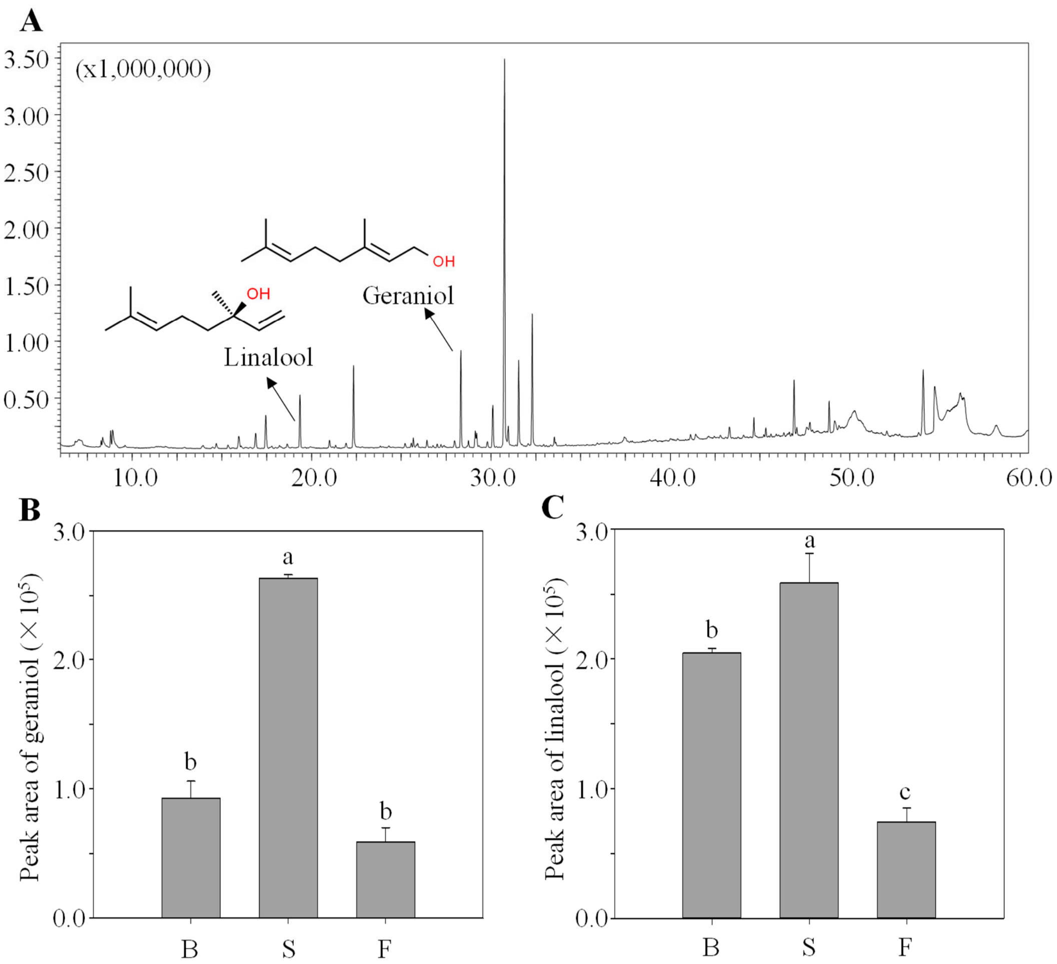

Figure S2 Content of geraniol and linalool in *D. officinale* at three flowering stages: budding (B), semi-flowering (S) and full flowering (F). Each bar represents the mean (± standard error, n = 3) of three independent biological replicates. Different letters above the bars indicate significant differences ($p < 0.05$, Duncan's multiple range test).

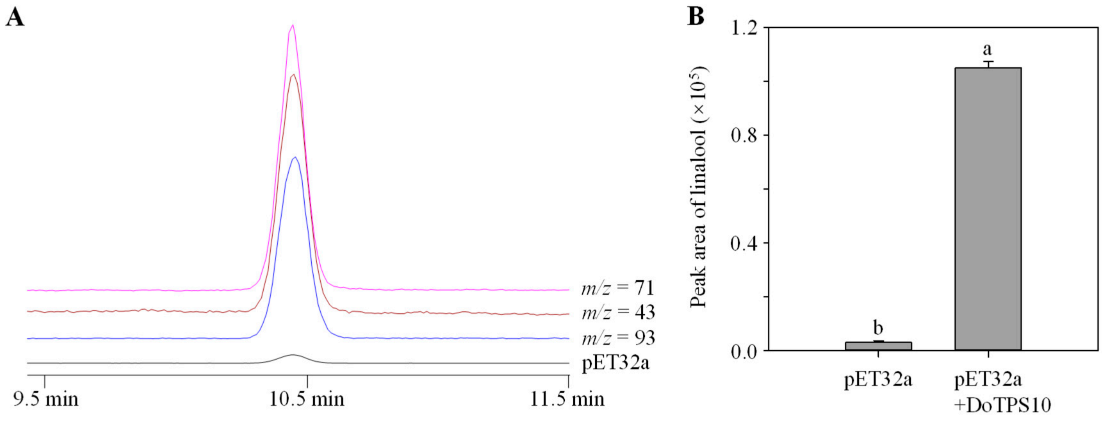

Figure S3 Gas chromatograms of products yielded by DoTPS10 using GPP as substrate. Each bar represents the mean (± standard error, n = 3) of three independent biological replicates. Different letters above the bars indicate significant differences ($p < 0.05$, Duncan's multiple range test).
